# A Chiral ^19^F NMR Reporter of Foldamer Conformation in Bilayers

**DOI:** 10.1021/jacs.2c09103

**Published:** 2022-11-15

**Authors:** Siyuan Wang, Flavio della Sala, Matthew J. Cliff, George F. S. Whitehead, Iñigo J. Vitórica-Yrezábal, Simon J. Webb

**Affiliations:** † Department of Chemistry, University of Manchester, Oxford Road, ManchesterM13 9PL, U.K.; ‡ Manchester Institute of Biotechnology, University of Manchester, 131 Princess Street, ManchesterM1 7DN, U.K.

## Abstract

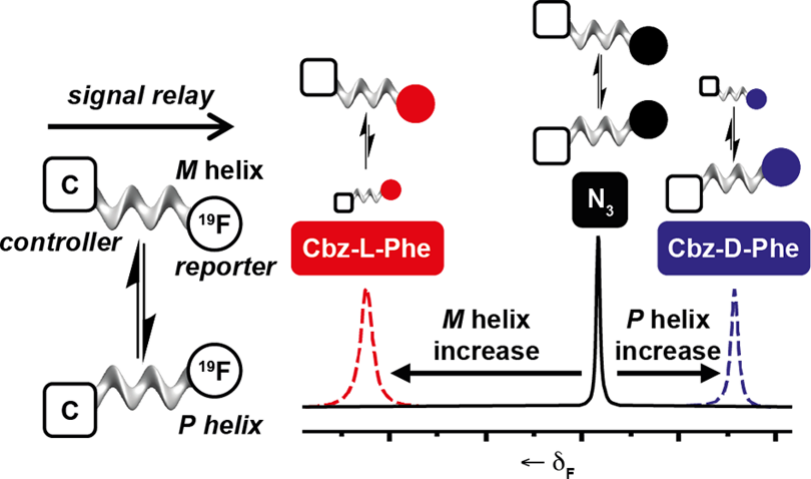

Understanding and controlling peptide foldamer conformation in phospholipid bilayers is a key step toward their use as molecular information relays in membranes. To this end, a new ^19^F “reporter” tag has been developed and attached to dynamic peptide foldamers. The (*R*)-1-(trifluoromethyl)ethylamido ((*R*)-TFEA) reporter was attached to the C-terminus of α-amino-*iso*-butyric acid (Aib) foldamers. Crystallography confirmed that the foldamers adopted 3_10_ helical conformations. Variable temperature (VT) NMR spectroscopy in organic solvents showed that the (*R*)-TFEA reporter had an intrinsic preference for *P* helicity, but the overall screw-sense was dominated by a chiral “controller” at the N-terminus. The ^19^F NMR chemical shift of the CF_3_ resonance was correlated with the ability of different N-terminal groups to induce either an *M* or a *P* helix in solution. In bilayers, a similar correlation was found. Solution ^19^F NMR spectroscopy on small unilamellar vesicle (SUV) suspensions containing the same family of (*R*)-TFEA-labeled foldamers showed broadened but resolvable ^19^F resonances, with each chemical shift mirroring their relative positions in organic solvents. These studies showed that foldamer conformational preferences are the same in phospholipid bilayers as in organic solvents and also revealed that phospholipid chirality has little influence on conformation.

## Introduction

The development of synthetic oligomers able to mimic the function of transmembrane proteins will provide insights into key cellular processes, such as the transit of molecules and information across membranes.^1,2^ The ability to transmit molecular information across multinanometer distances (i.e., the width of a membrane) could also provide technology for new fields like molecular robotics,^3^ for example, by transferring instructions to a catalytic centre.^4^ Synthetic folded oligomers, foldamers, have shown great promise for mimicking the behavior of biological folded oligomers, such as proteins and oligosaccharides. The folded conformations of these oligomers can be either static or dynamic at room temperature, in the latter case often rapidly interconverting between a few similarly stable conformations^5^ that can be made responsive external stimuli.^6^ α-Amino-*iso*-butyric acid (Aib) foldamers are a well-studied class of dynamic foldamer with two principal conformations: 3_10_ helices of either right-handed (*P*) or left-handed (*M*) screw sense. These rodlike hydrophobic peptides can embed into membranes; if long enough, they can span bilayers to produce ion channels and antibiotic activity that is reminiscent of the peptaibol antibiotics.^7^ Furthermore, using an external stimulus to switch between *P* and *M* helical sense along the entire length of a membrane-embedded Aib foldamers is proposed to mimic a key process in biological signal transduction, the relay of information across the multinanometer thickness of biological membranes.^6a,6b^

To design and operate such complex supramolecular systems, the conformational relay found in Aib foldamers needs to be understood and optimized in bilayers, which are complex anisotropic environments. We have recently shown that vibrational circular dichroism, Raman optical activity, fluorescence spectroscopy, and ^19^F solid-state NMR (ssNMR) spectroscopy can all provide complementary information on Aib foldamer conformation in lipid bilayers.^6a,6b,8−10^ In the latter case, the orthogonality and the high sensitivity of ^19^F nuclei facilitates NMR analysis in the ^1^H and ^13^C rich lipid matrix, where long acquisition times and line broadening are significant problems.^11−15^

^19^F ssNMR spectroscopy can provide detailed insights into the conformations of membrane-bound peptides,^11,12d,16^ but the conditions used for ssNMR are not suitable for intact phospholipid vesicles. Phospholipid vesicle suspensions are biomimetic systems that better reflect the compartmentalized nature of intact cells.^17^ Although the anisotropy and slow tumbling rates of phospholipid vesicles broadens the resonances of vesicle-bound molecules, solution NMR spectroscopy on small unilamellar vesicles (SUVs) can provide useful information. For example, SUV studies have revealed differences between the inner and outer leaflet environments^18^ and how peptide-membrane interactions depend on the bilayer curvature.^19^

**Figure 1:**
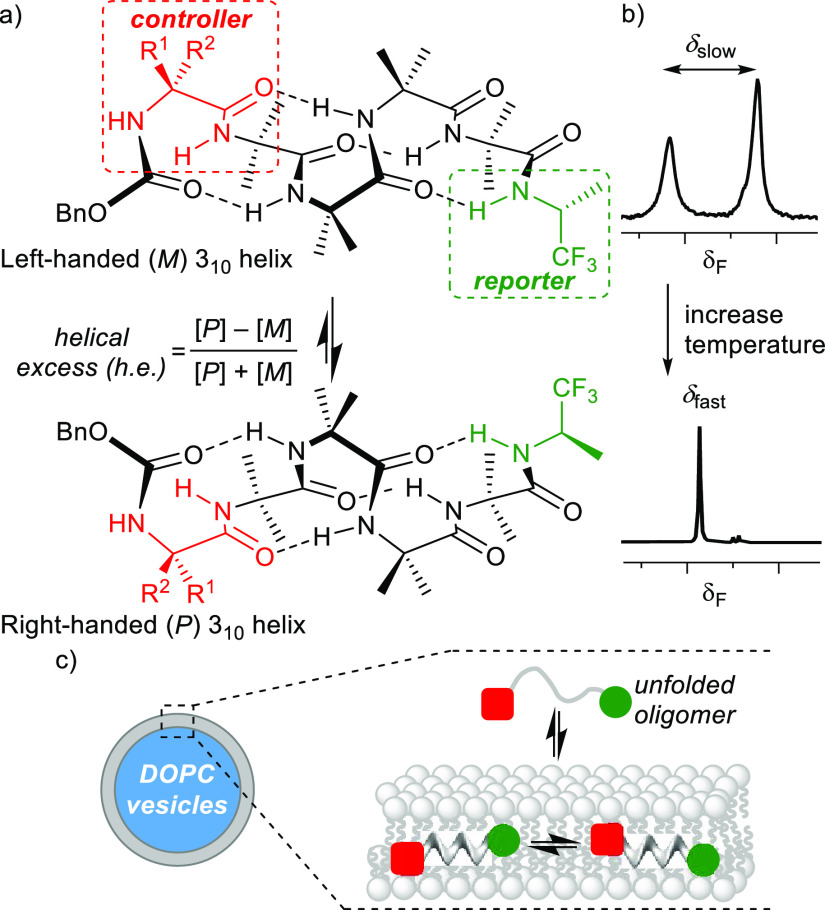
(a) Aib foldamer with N-terminal controller (in red) and C-terminal (*R*)-TFEA reporter (in green), showing interconversion of *M* and *P* 3_10_ helical conformations. (b) Expected ^19^F NMR signals from a foldamer with unequal populations of *M* and *P* 3_10_ helical conformations that are in slow exchange (top) and fast exchange (bottom); the latter gives a singlet at the weighted average chemical shift. (c) Schematic representation of an Aib foldamer partitioning between aqueous buffer and the membrane of a DOPC SUV.

^19^F NMR reporter tags on Aib foldamers have been used to report on the screw-sense ratio in organic solvents and phospholipid bilayers, using solution NMR and ssNMR spectroscopy, respectively. If the tag is achiral, such as the β,β′-difluoro-Aib (“Fib”) tag used by De Poli et al.,^6a^ the ^19^F-containing reporter group can provide the magnitude of the excess of one helicity over the other (the “helical excess”, h.e., Figure 1) but will not reveal which helical sense is favored; to do so, a chiral reporter group is required. Chiral NMR reporter groups permit the preferred helical sense to be determined because the helical conformations are diastereomeric and have different spectroscopic properties. Because the reporter group itself is chiral, it may also exert a helical preference, although this preference should be as low as possible.^20^

Our design of a new ^19^F NMR reporter involved the replacement of a methyl in an *iso*-propyl group with an isosteric trifluoromethyl (CF_3_). This was hoped to generate a chiral fluorinated reporter with two substituents of similar size, which might only generate a small helical preference. However, Bodero et al. reported that stereoselectively replacing a single methyl on Aib with CF_3_ provides a residue ((*S*)-TfmAla) that acts as an N-terminal controller for Aib foldamers, producing a significant h.e._0_ of +80.^21^ The (*R*)-1-(trifluoromethyl)ethylamido ((*R*)-TFEA) reporter (Figure 1a, in green) was designed with this isosteric relationship in mind; it was hoped any intrinsic helical preference would be diminished by attachment to the C-terminus.^22^ Both enantiomers of the amine are commercially available, which will allow the effect of phospholipid chirality to be probed (Figure 1c). The (*R*)-TFEA reporter should also be relatively stable to most externally added reagents. The report it provides will depend on foldamer dynamics. If exchange between *P* and *M* helical conformations is slow, then each diastereomeric conformation will provide a distinct resonance and the integral of each will give the *P*/*M* ratio. However, if conformational exchange is fast on the ^19^F NMR timescale, then a weighted average signal will result with a chemical shift that reflects the *P*/*M* ratio (Figure 1b).

Herein, we describe the synthesis and study of a family of Aib foldamers bearing this C-terminal (*R*)-TFEA reporter group. These studies revealed how the ^19^F NMR resonance of the CF_3_ group responded to the screw-sense preference of different N-terminal residues in organic solvents and showed for the first time how solution NMR spectroscopy can reveal these conformational preferences in the bilayers of small unilamellar vesicles (SUVs).

## Results and Discussion

**Scheme 1:**
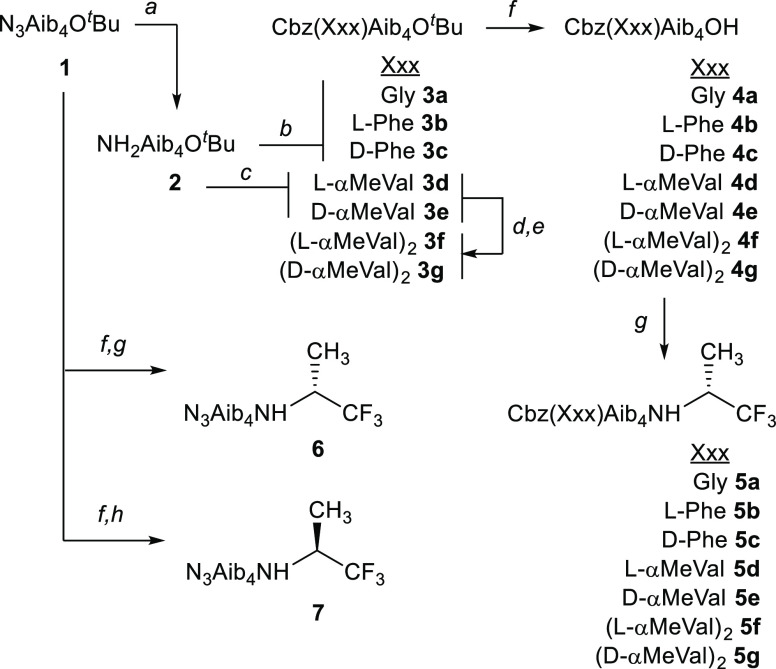
Synthetic Strategy for Aib Tetramers **5a–g**, **6**, and **7**^a^ (a) H_2_/Pd(C), EtOH, rt; (b) Cbz(Gly)OH or Cbz(d/l-Phe)OH, EDC^.^HCl, HOBt, DIPEA, CH_2_Cl_2_, rt; (c) (i) Cbz(d/l-αMeVal)OH, cyanuric fluoride, pyridine, CH_2_Cl_2_, rt; (ii) **2**, CH_2_Cl_2_, DIPEA rt; (d) **3d** or **3e**, H_2_/Pd(C), MeOH, rt; (e) (i) Cbz(d/l-αMeVal)OH, tetramethylfluoroformamidinium hexafluorophosphate, pyridine, CH_2_Cl_2_, rt; (ii) DIPEA, CH_2_Cl_2_, rt; (f) CF_3_CO_2_H, CH_2_Cl_2_, rt; (g) (*R*)-2-amino-1,1,1-trifluoropropane^.^HCl, DIPEA, HATU, rt; and (h) (*S*)-2-amino-1,1,1-trifluoropropane^.^HCl, DIPEA, HATU, rt.

The synthetic versatility of the (*R*)-TFEA reporter group allowed it to be installed in two steps from the readily accessible protected Aib tetramers **1** and **3a**–**3g** (Scheme 1).^6b,7,23^ This gave the family of Aib tetramers **5a**–**g** and **6**. Foldamers **5b**–**5g** can be grouped into three pairs of diastereomers that have either the l- or d-enantiomer of a chiral residue at the N-terminus. Each N-terminal residue has a different ability to induce an h.e. in the following Aib chain. An N-terminal azide or Cbz(Gly) residue will not induce a screw-sense preference, but Cbz-capped phenylalanine (Phe), α-methylvaline (αMeVal), and (αMeVal)_2_ are all reported to induce an h.e. in Aib tetramers. Phe has a moderate ability to induce an h.e. whereas αMeVal and (αMeVal)_2_ are much stronger inducers of h.e.^23a,24^ If the (*R*)-TFEA reporter at the C-terminus has a screw-sense preference, then one of the diastereomers in each pair will have the terminal groups inducing the same screw-sense preference (i.e., both *M* or both *P*, which gives a screw-sense “match”) with the other diastereoisomer having opposing screw-sense preferences (i.e., one is *M* and the other *P*, which gives a screw-sense “mismatch”).

The Fourier transform infrared (FT-IR) spectra of **5a** and **6** implied that 3_10_ helices were formed. The azide-capped tetramer **6** in the solid state shows a strong band at 1661 cm^–1^ in the amide I region, which is diagnostic of a 3_10_ helix (Figure S1, **6** also shows a strong azide band at 2110 cm^–1^).^8a^ The corresponding FT-IR band for the glycine-capped compound **5a** is found at 1655 cm^–1^ (Figure S2), which is between the regions typical for 3_10_-helix (1658–1666 cm^–1^) and α-helix (1650–1658 cm^–1^).^25^

The circular dichroism (CD) spectrum of foldamer **6** with azide at the N-terminus was weak in acetonitrile and in methanol when compared to those foldamers with chiral N-terminal residues (Figures S3 and S4). The CD spectra of diastereomeric pairs of foldamers (**5b**/**c**, **5d**/**e**, **5f**/**g**) are not equal and opposite, although some pairs in CH_3_CN, such as **5b/c**, **5d/e**, and **5f/g**, gave almost mirror image spectra. These observations suggested that the chiral (*R*)-TFEA reporter may exert relatively weak conformational control.

## Solid-State Structures

**Figure 2:**
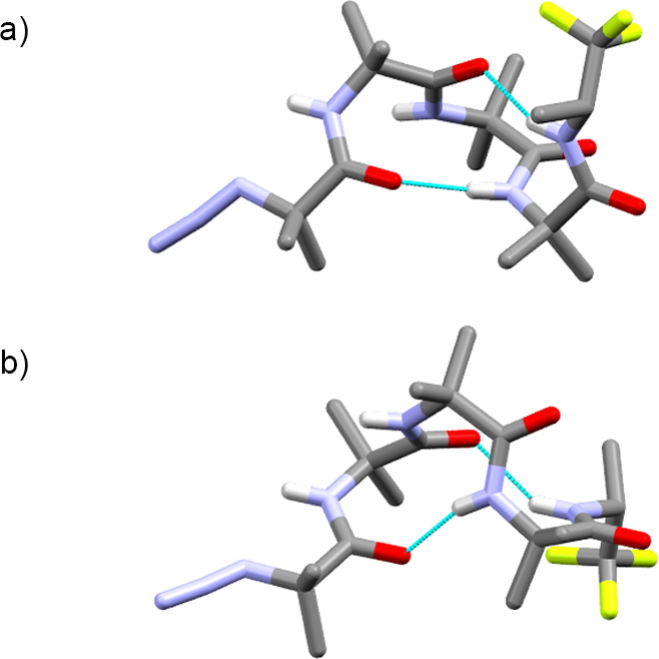
Solid-state structures showing (a) *M* and (b) *P* helical conformations adopted by **6** in the unit cell, with intramolecular hydrogen bonds shown. C atoms are shown in gray, N in light blue, O in red, and F in pale green. Some H atoms have been removed for clarity.

A weak chiral influence from the (*R*)-TFEA reporter was also implied by the crystal structure of **6** (Figure 2). The solid-state structure shows two molecules in the unit cell, one with a *P* 3_10_ helical structure (Figure 2b) and the other with a 3_10_ helical structure of the opposite, *M*, sense (Figure 2a). The respective distorted 3_10_ helices are maintained by an intramolecular *i* → *i* + 3 hydrogen bonding pattern: one hydrogen bond between the C=O of the first Aib and the NH of the fourth Aib and the other between the C=O of the second Aib and the NH of the (*R*)-TFEA. The C-N dihedral angle in the reporter group in both *M* and *P* structures places the C–H *anti* to the N–H, mirroring the calculated antiperiplanar geometry of acetylated (*R*)-TFEA, AcNHCH(CF_3_)CH_3_.^26^ The C=O of the third and fourth Aib of the *P* helical foldamer form intermolecular hydrogen bonds with the NH of the first and second Aib residues of the neighboring *M* helical foldamer.

**Figure 3:**
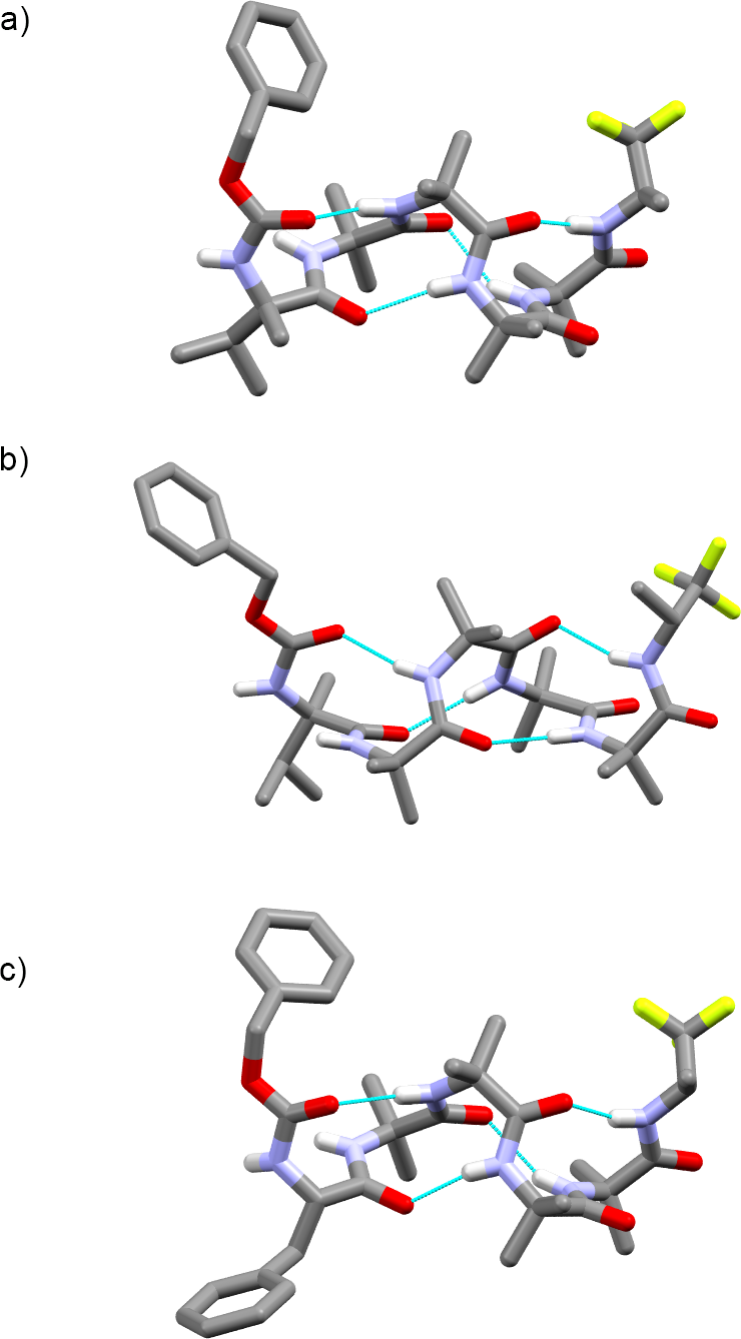
Solid-state structures of (a) l-αMeVal capped tetramer **5d** (*P* helix), (b) d-αMeVal capped tetramer **5e** (*M* helix), and (c) l-Phe capped tetramer **5b** (*P* helix). Intramolecular hydrogen bonds are shown. C atoms are shown in gray, N in light blue, O in red, and F in pale green. Some H atoms have been removed and molecules of solvation not shown for clarity.

The foldamer with an l-αMeVal cap, which favors a *P* helix,^23a,24^ also gave crystals suitable for X-ray crystallographic structure determination (**5d**, Figure 3a). Once again, a 3_10_ helix is adopted by the Aib foldamer body, with the expected *P* helical sense. The folding around the chiral residue shows that it has adopted a *P* type III β turn, with φ/ψ angles for the αMeVal and first Aib of −51°/–57° and −41°/–23°, respectively, similar to the ideal values of φ = −60° and ψ = −30°.^27^ Its diastereomeric counterpart, **5e**, bearing a d-αMeVal cap that favors an *M* helix, was also crystallized (Figure 3b). The 3_10_ helix adopted by the Aib foldamer body now has an *M* helical sense. An *M* type III’ β turn,^28^ with φ/ψ angles for the αMeVal and first Aib of +57°/+34° and +55°/+29° was observed at the N-terminus. The conformation in the (*R*)-TFEA reporter group in **5e** is the same as in **5d**, but the helix loop is now on the same side as the CF_3_ group.

Foldamer **5b** with an l-Phe cap also gave crystals suitable for structure determination (Figure 3c). The solid-state structure showed that the foldamer had in fact adopted a *P* 3_10_ helix in a structure analogous to that of **5d**. This is the opposite of the *M* helix preferred by N-terminal Phe residues in solution and is due to the Phe residue adopting a type III β turn rather than the expected type II turn.^29^ It is reported that crystal packing forces can compete against the helical preference of a terminal chiral group if that preference is relatively weak, illustrated by the solid-state structures of Aib tetramers with either a Cbz(l-Val) cap^30^ or a Cbz(d-Phe) cap.^9^

## Solution-Phase ^19^F NMR Spectroscopy of Foldamers in Organic Solvents

Given the reported dynamic behavior of Aib tetramers, it was anticipated that rapid exchange between *P* and *M* helical conformations in foldamers **5a**–**g** on the ^1^H NMR timescale would lead to the observation of weighted average spectra for each compound.^30^

^1^H NMR spectroscopy of foldamer **5a** may provide insight into the ability of the (*R*)-TFEA reporter to induce a screw-sense preference. Fast exchange and a large h.e. in the Aib foldamer could cause the methylene protons of Gly in the foldamer to appear diastereotopic.^30^ However the ^1^H NMR spectrum of **5a** in CD_3_CN and CD_3_OD at 298 K showed an unsplit but broadened resonance from these protons (Figures S5a and S6a). Although this observation could at first glance imply that the h.e. induced by a (*R*)-TFEA reporter is low, the relay of chirality from C-terminus to N-terminus is known to be less efficient than that for the reverse sense.^22^ In addition, the N-terminal Gly signal may not be especially sensitive to h.e. Indeed, comparison of the ^1^H NMR spectra of diastereomeric pairs of foldamers showed small differences that can only arise if the (*R*)-TFEA reporter is able to induce a local h.e., which is communicated down the helix. This is exemplified by l- and d-αMeVal-capped foldamers **5d** and **5e**, where the diastereotopic methyl groups of the *iso*-propyl in αMeVal have larger separation in **5e** compared with those of **5d** and the α-methyl chemical shifts are different by ca. 0.1 ppm (Figures S5b and S6b).

**Figure 4:**
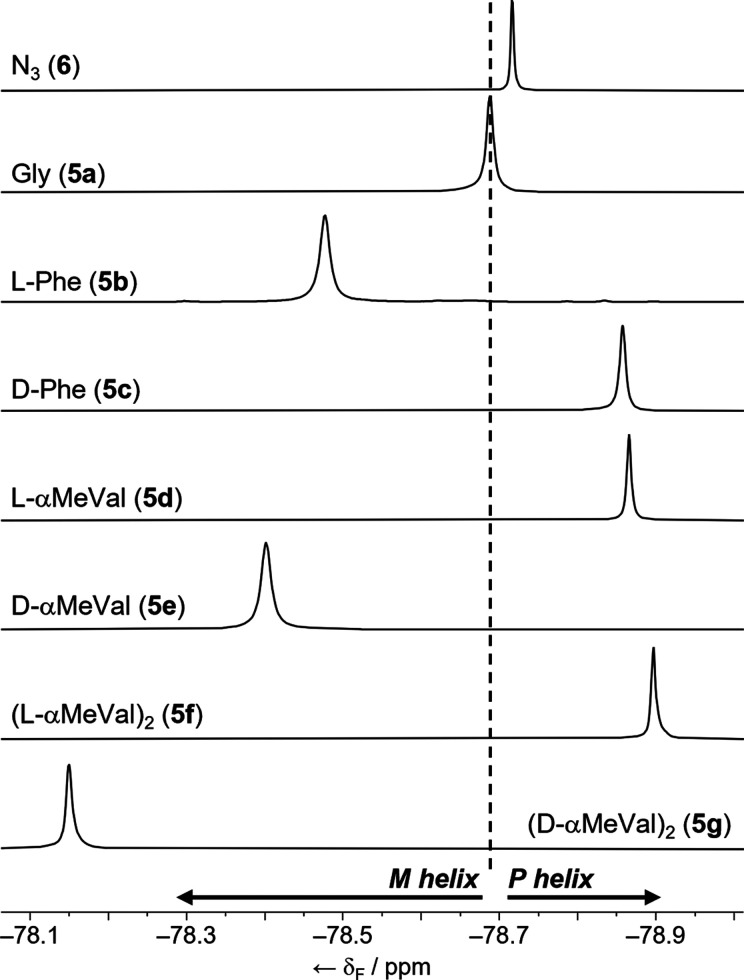
Partial ^19^F NMR spectra (CD_3_OD, all 376 MHz except **5b** 470 MHz, 298 K) of, from top to bottom, **6**, **5a**–**g**. Spectra referenced with C_6_F_6_ at −165.37 ppm (Figure S8).^31^

^19^F NMR spectroscopy of foldamers **5a–5g** and **6** at 298 K in CD_3_OD gave spectra consistent with fast exchange between conformations. Foldamers **5a** and **6**, with achiral N-terminal residues, each showed a single resonance at, respectively, −78.688 and −78.716 ppm (Figure 4).^31^ These resonances are the weighted average of the chemical shifts for each diastereomeric conformation, which contain either a *P* or an *M* helix. As a weighted average, the position of this resonance should be directly related to the h.e. for each foldamer.^32^ Because neither azide nor Cbz(Gly) are chiral, the *P*/*M* ratio is determined by the inductive power of the (*R*)-TFEA reporter and should be the same for each. Indeed, these chemical shift values are similar, which also suggests that the reporter is not directly interacting with the N-terminal group. The small −28 ppb difference between **5a** and **6** may be due to the greater hydrogen bonding ability of a Cbz(Gly) terminus stabilizing the 3_10_-helix to a greater extent than an azide terminus.^8a^

A chiral controller group at the Aib N-terminus will alter the *P*/*M* ratio and should produce changes in the chemical shift of the CF_3_ group (δ(CF_3_)) relative to **5a**, which has the same hydrogen bond ability as **5b**–**5e**. In CD_3_OD, the Cbz(l-Phe) controller in **5b** shifts the resonance by +0.211 ppm relative to that of **5a** (Figure 4, positive values indicate a shift downfield), whereas the N-terminal Cbz(d-Phe) controller in **5c** moves the resonance in the opposite direction (−0.169 ppm). An αMeVal group exerts stronger conformational control^30^ and has a correspondingly stronger effect on the position of the CF_3_ resonance. A Cbz(l-αMeVal) group shifts the resonance by −0.178 ppm, whereas an N-terminal Cbz(d-αMeVal) group shifts the resonance by +0.286 ppm. Adding another αMeVal group gives the strongest covalent controller reported to date^30^ and accordingly produces the strongest change in the location of the CF_3_ resonance (−0.209 ppm for Cbz(l-αMeVal)_2_ and +0.538 ppm for Cbz(d-αMeVal)_2_). Changing the solvent to CD_3_CN gave very similar observations, indicating that reporter function is largely solvent-independent (Table S1 and Figures S7 and S8).

The magnitude and direction of these changes in δ(CF_3_) broadly correlate with the reported abilities of each N-terminal residue to favor one screw sense over the other. The strength of the screw-sense preference of a given controller is often presented as an inferred helical excess (h.e._0_), which corresponds to the helical excess adjacent to the controller at the foldamer N-terminus.^21,30^ Phe induces a moderate h.e._0_ in the closest part of the helix (−52% for l, +52% for d), whereas αMeVal (+68% for l, −68% for d) and (αMeVal)_2_ (+95% for l, −95% for d) are stronger inducers of h.e._0_.^30^ These h.e._0_ values show that Cbz(l-Phe) and Cbz(d-αMeVal) both favor *M* helicity; they produce a downfield shift in the ^19^F NMR resonance of the reporter. On the other hand, Cbz(d-Phe) and Cbz(l-αMeVal) favor *P* helicity and produce an upfield shift in δ(CF_3_), albeit of smaller magnitude.

Smaller changes of δ(CF_3_) for foldamers with chiral controllers that favor *P* helicity compared to foldamers with the enantiomeric controllers that favor *M* helicity suggest that the (*R*)-TFEA reporter intrinsically produces an excess of *P* helix. This qualitative observation also suggests that it might be possible to devise a model that predicts δ(CF_3_) at 298 K by accounting for the combined screw-sense preferences of each controller and (*R*)-TFEA.

## VT-NMR and Modeling

**Figure 5:**
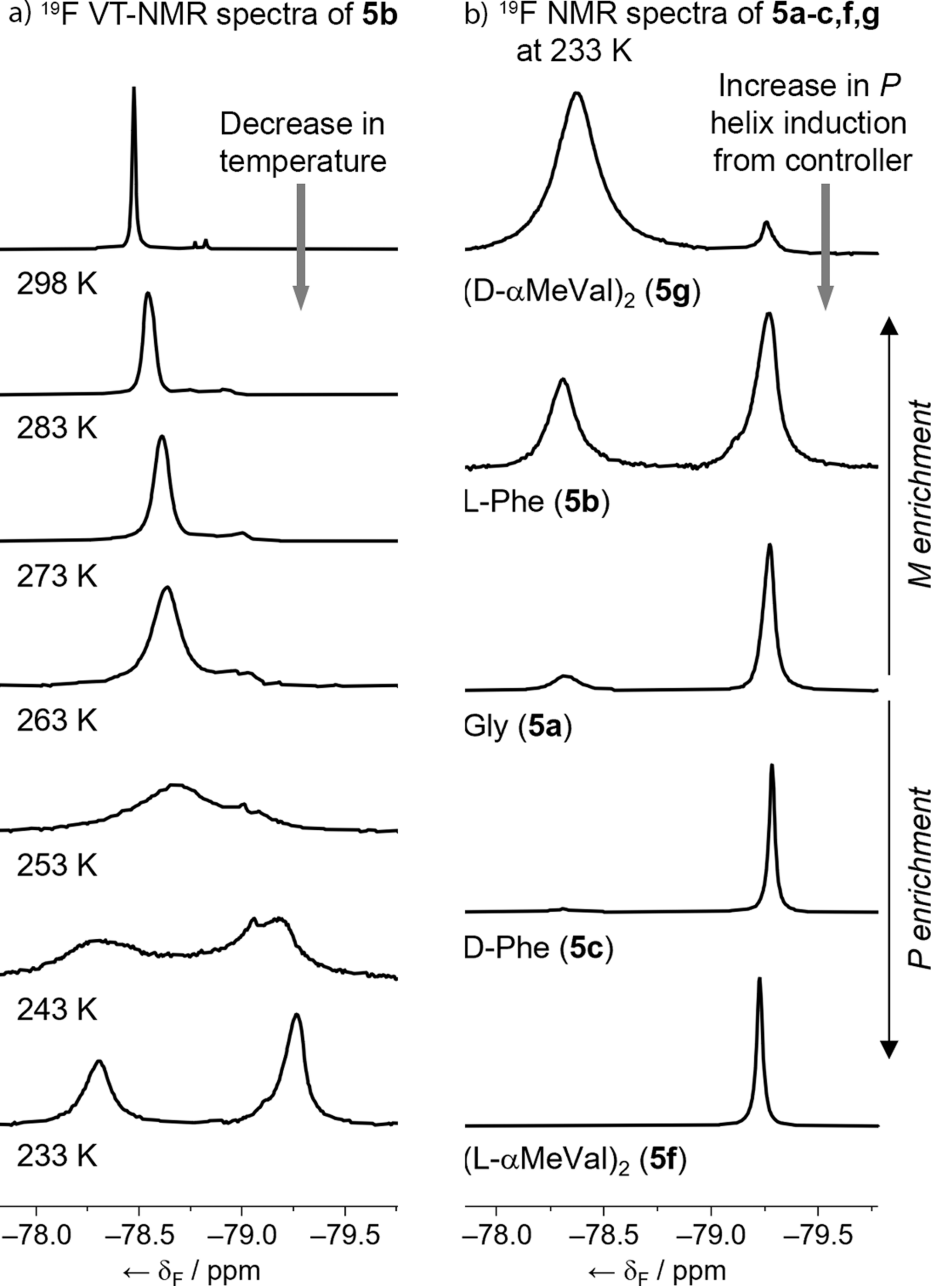
(a) ^19^F VT-NMR spectra (CD_3_OD, 470 MHz) for l-Phe capped tetramer **5b** showing the CF_3_ resonance. (b) Partial ^19^F NMR spectra (CD_3_OD, 470 MHz, 233 K) showing CF_3_ resonance of (d-αMeVal)_2_ (**5g**), l-Phe (**5b**), Gly (**5a**), d-Phe (**5c**), and (l-αMeVal)_2_ (**5f**). Spectra proton decoupled and referenced to C_6_F_6_ at −165.37 ppm.^31^

Variable temperature (VT) ^19^F NMR spectroscopy on **5b** in CD_3_OD confirmed that this foldamer was in fast exchange at room temperature, as the single ^19^F resonance shifted and decoalesced to give a pair of resonances at −78.31 and −79.27 ppm at 233 K. These peaks were of unequal height (Figures 5a and S9) and integrated in an approximately 0.75:1 ratio, respectively.

The diastereomeric foldamer **5c** presented the same resonances but in a 0.04:1 ratio. Extending these ^19^F VT-NMR studies to Gly-capped **5a** and (d-αMeVal)_2_-capped **5g** showed that each had the same two peaks, but in 0.27:1 and 20:1 ratios, respectively. Foldamer **5f** only showed one peak at −79.22 ppm, suggesting that the other peak at ca. −78.37 ppm is below the detection limit (i.e., <0.01:1 ratio).

These VT-NMR studies are consistent with interchange between *P* and *M* 3_10_ helices becoming slow at low temperatures. Furthermore, because (d-αMeVal)_2_ is reported to induce *M* helicity in Aib foldamers,^9,30^ the larger resonance at ca. −78.37 ppm in its ^19^F NMR spectrum at 233 K is likely to be from the *M* helical conformer (Figure 5b). Conversely (l-αMeVal)_2_ induces *P* helicity, and the largest resonance is the one at ca. −79.22 ppm. Based upon these assumptions, foldamer **5a** shows *P*/*M* ∼ 4, which confirms that the (*R*)-TFEA reporter group can induce a *P* helix. This ratio appears to be relatively solvent-independent; **5a** in CD_3_CN at 233 K shows a 0.27:1 ratio for ^19^F NMR resonances at −79.62 ppm and −78.67 ppm (Figure S10). The observation of slow exchange allows the direct calculation of the observed helical excess (h.e._obs_).^30^ For example, most powerful controllers, (d-αMeVal)_2_ and (l-αMeVal)_2_, produce h.e._obs_ values at 233 K of −0.91 and >0.99, respectively, which at 298 K correspond to ^19^F chemical shifts of −78.150 and −78.897 ppm.

**Figure 6:**
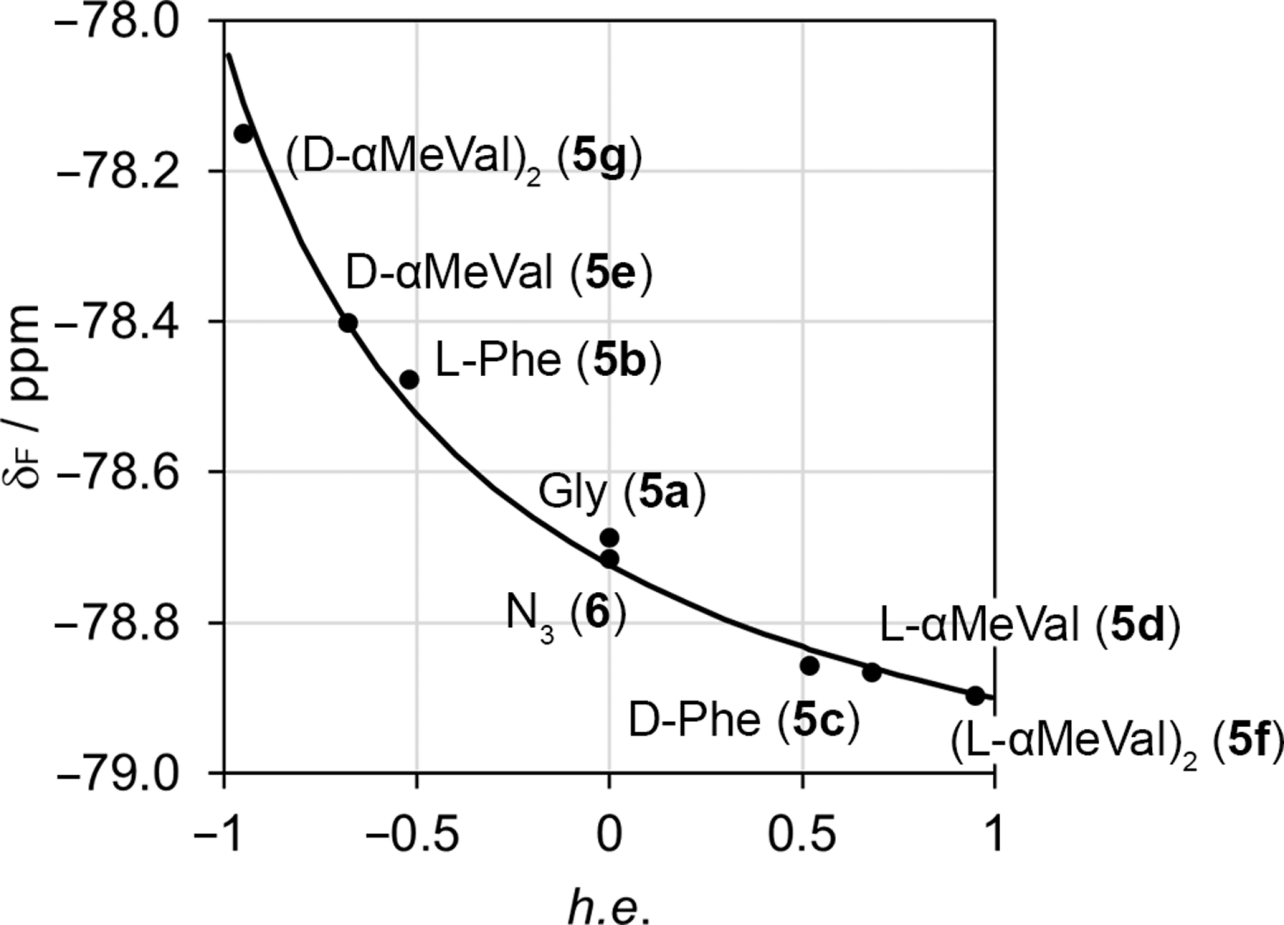
^19^F NMR chemical shifts in CD_3_OD at 298 K for Aib tetramers **5a**–**5g** and **6** correlated with the reported ability of each chiral N-terminal group to induce a local helical excess (h.e._0_).^30^ Curve fit assumes a preference of (*R*)-TFEA for right-handed helix (Δ*G*_R_) of −3.40 kJ mol^–1^. Chemical shifts calibrated to C_6_F_6_.^31^

To elaborate the relationship between δ(CF_3_) and the identity of each N-terminal residue, a model was developed that correlated the reported h.e._0_ for each N-terminal group^30^ with the measured δ(CF_3_) of **5a**–**g** and **6**. The VT-NMR spectrum of **5a** at low temperatures provides the *P*/*M* ratio induced by the reporter group (∼3.76 in CD_3_OD). A free energy change (−3.4 kJ mol^–1^) can be estimated from this preference of the reporter for right-handed helix (Δ*G*_R_). Then, the reported h.e._0_ induced by each controller residue can be converted into a Gibbs free energy (Δ*G_C_*) and then added to Δ*G*_R_. The resulting net free energy change (Δ*G_C_* + Δ*G*_R_) can be converted into a *K* value (the *P*/*M* ratio) for each foldamer (see the Supporting Information, Section 6). This calculation of *K* can also be represented in terms of h.e._0_ and Δ*G*_R_ (eq 1).
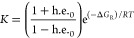
1The weighted average ^19^F NMR signal at 298 K for each foldamer is then calculated using estimated δ_M_ = −78.03 ppm (for *M* helix) and δ_P_ = −78.90 ppm (for *P* helix) (eq 2).
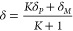
2This model gave good agreement with the measured chemical shifts at 298 K across the family **5a**–**5g** and **6** (Figure 6). It also replicated the nonlinear shape of the curve (not symmetrical about h.e._0_ = 0), which occurs because the reporter itself has a chiral influence that induces a *P* helical screw sense. Those controller groups that also induce a *P* helical screw sense have less scope to increase the proportion of *P* helix; conversely, controllers that induce *M* screw sense have greater scope to do so. The net effect is that the change in chemical shift for different controllers is greatest for those that counteract the screw-sense preference of the (*R*)-TFEA reporter.

This model also approximately replicated the *P*/*M* ratio derived from the two signals observed at 233 K in the VT-NMR data for compounds **5b** (calculated as 1.25:1)**, 5c** (calculated as 12.5:1)**, 5f** (calculated as 154:1), and **5g** (calculated as 0.01:1, Table S3). A similar treatment of the ^19^F VT-NMR data for foldamers in CD_3_CN (**5a** gave *P*/*M* of 3.70 and Δ*G*_R_ = −3.22 kJ mol^–1^) also gave a good fit to the measured chemical shifts at room temperature in this solvent (Figure S11).

## Solution-Phase ^19^F NMR Spectroscopy of Foldamers in Vesicle Membranes

**Figure 7:**
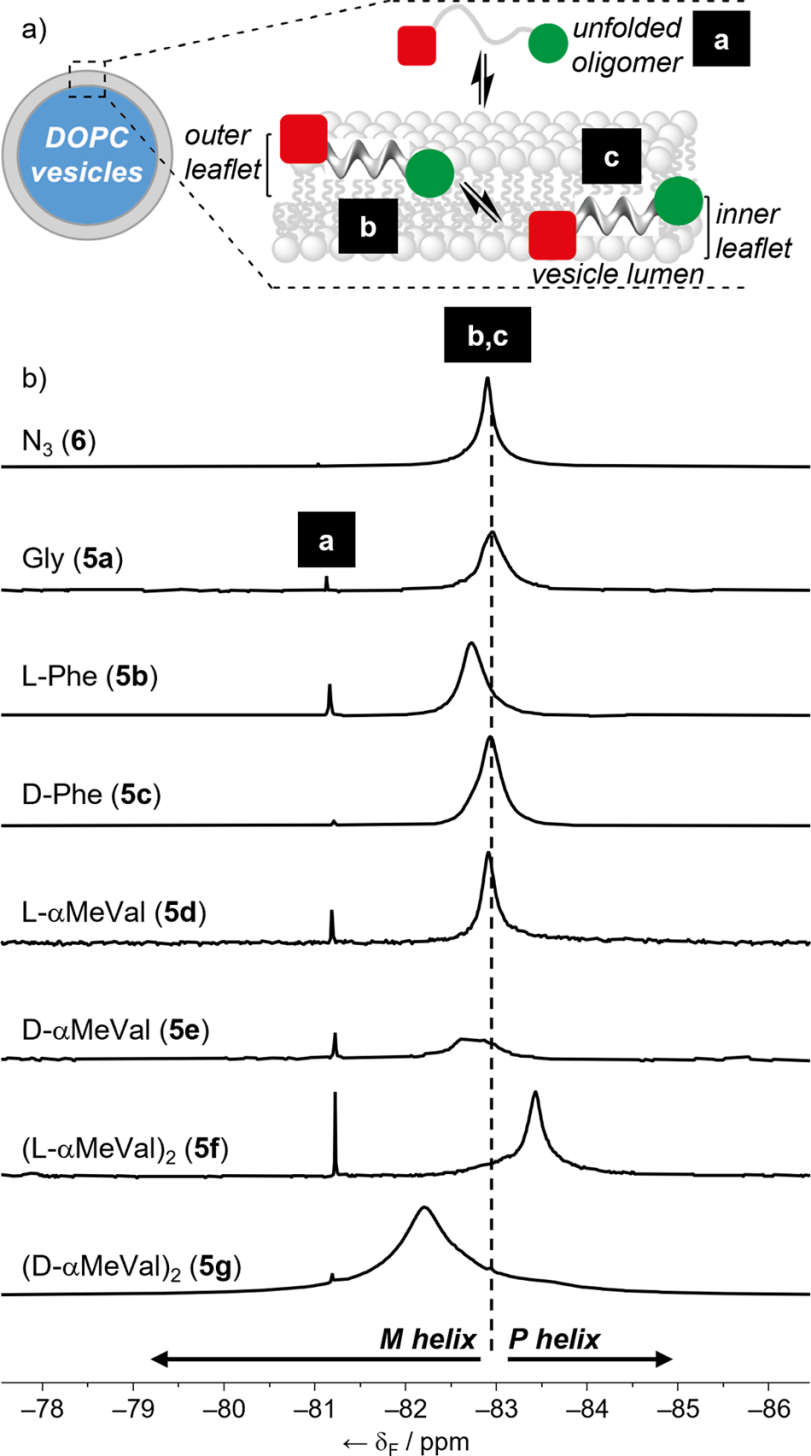
(a) Schematic representation of an Aib foldamer in buffer (“a”) partitioning into a vesicle bilayer and locating in either the inner (“b”) or the outer leaflet (“c”). (b) Partial ^19^F NMR spectra (1:9 D_2_O/H_2_O, 470 MHz, 298 K, [NaCl] = 100 mM, [MOPS] = 20 mM, pH 7.4; 376 MHz for **5b**) of foldamers **5a**-**5g** and **6** in DOPC SUVs. Unincorporated foldamer at −81.2 ppm (environment “a”) and incorporated foldamer at −82 to −84 ppm (environments “b” and “c”). Spectra referenced to KF at −125.300 ppm.

Previously, ssNMR ^19^F spectroscopy showed the achiral ^19^F bearing “Fib” residue at the C-terminus of Aib foldamers gave resolvable resonances that reported on changes in conformation (the full width at half maximum (FWHM) was ca. >0.6 ppm).^6a^ However the rapid spinning of ssNMR samples prevents the use of intact vesicles, so solution-phase NMR spectroscopy of intact SUVs was explored. It was hoped that loading (*R*)-TFEA labeled foldamers into SUVs composed of a lipid that gives a very fluid bilayer, 1,2-di-(9Z-octadecenoyl)-*sn*-glycero-3-phosphocholine (DOPC), would lead to suitably narrow resonances (Figure 7a).

Because of expected low signal strength caused by line broadening, a high concentration of lipids (50 mM) and a high membrane loading of 11 mol % foldamer were used. SUVs were formed by 4 h bath sonication at 25 °C of suspensions of DOPC mixed with Aib foldamers **5a–g**, **6**, or a racemic mixture of **5b** and **5c** in buffered 1:9 D_2_O:H_2_O ([NaCl] = 100 mM, [MOPS] = 20 mM, pH 7.4), according to reported methodologies.^33^ Dynamic light scattering (DLS) showed that this procedure produced uniform and reproducible samples of SUVs with a hydrodynamic diameter of 30 to 40 nm (Figure S13).

The ^19^F NMR spectrum of each of these SUV suspensions showed a sharp peak at ca. −81.2 ppm and a broad peak between −82 and −84 ppm. The position of the sharp peak was similar between SUVs containing different foldamers, but the chemical shift of the broad peak, which had a FWHM typically around 0.3 ppm, was significantly different depending upon the identity of the N-terminal residue (Figure 7b).

To understand the appearance of these spectra, SUVs containing l-Phe capped foldamer **5b** were analyzed further. The narrow linewidth of the sharp peak and its position at −81.162 ppm suggested that this peak arose from foldamer **5b** that was unfolded in buffer^34^ and not incorporated in the SUVs (Figure 7a, labeled as “a”). This assignment was supported by ^19^F DOSY spectroscopy (Figure S14), which gave a diffusion coefficient of 9.85 × 10^–6^ cm^2^s^–1^ for the sharp peak and 9.36 × 10^–7^ cm^2^s^–1^ for the broad peak; the latter value is close to that reported for SUVs.^35^ Furthermore, purification of the SUV suspension by size exclusion chromatography (SEC) removed the sharp peak from the spectrum, leaving the broad resonance that was ascribed to the foldamer in the membrane (Figure S15a). Mass spectrometric analysis of a SEC fraction containing the sharp peak confirmed that it contained unincorporated tetramer (Figure S15b). Some foldamers were more difficult to incorporate into SUV membranes than others because of their lower solubility in the chloroform used during SUV preparation; the maximum solubility of **5d** in CHCl_3_ was 288 mM, whereas the same value for its diastereomer **5e** was 50 mM (see the Supporting Information, Section 9). This difference in solubility may cause the greater broadness of the **5e** resonance, which was consistently observed in different preparations of **5e** in DOPC SUVs, although the position of the peak remained the same (δ_F_ = −82.71 ± 0.02 ppm). Indeed, the chemical shift of the broad peak for these foldamers was not sensitive to membrane loading, for example, **5c** gave the same chemical shift within the error over the range 2 to 11 mol % (see the Supporting Information, Section 8).

The position of the broad ^19^F NMR resonance for each embedded foldamer appears to reflect the ability of each controller to induce an h.e., which in turn confirms that the end-to-end helical relay is still present in DOPC bilayers. The foldamers capped with the best chiral controllers, (l-αMeVal)_2_ and (d-αMeVal)_2_, showed the broad peak either upfield (−83.426 ppm) or downfield (−82.211 ppm), respectively, compared to the foldamer with an achiral Gly cap (−82.915 ppm). The l-Phe and d-αMeVal capped foldamers, which have an excess of *M* helix, both show downfield shifted peaks, as observed in organic solvents. However, the d-Phe and l-αMeVal diastereomers show little difference in δ(CF_3_) compared to **5a** and **6**, suggesting that the reporter is even less responsive to increases in *P* helical conformation when in a bilayer. A similar effect was observed across a series of Fib-labeled Aib tetramers in membranes. ^19^F ssNMR showed that the response of the achiral Fib reporter to different chiral groups at the N-terminus was weaker in a membrane than in an organic solvent.^6a^ Although δ(CF_3_) values in DOPC SUVs are markedly different for foldamers **5b** and **5c** compared to their δ(CF_3_) values in organic solvents, the sense of the h.e. in each foldamer is the same in DOPC as it is in organic solvents. Similarly, the chemical shift difference between diastereomers **5b** and **5c** is comparable in each environment (−0.222 ppm in SUVs, −0.284 ppm in CD_3_CN).

**Figure 8:**
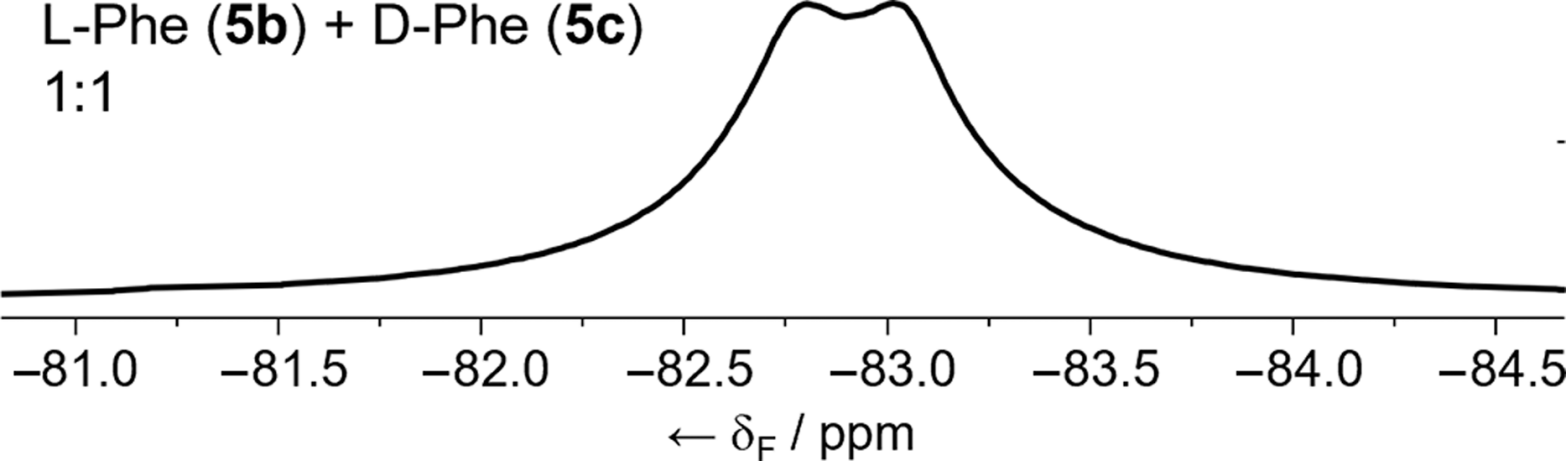
Partial ^19^F NMR spectrum (1:9 D_2_O/H_2_O, 470 MHz, 298 K, [NaCl] = 100 mM, [MOPS] = 20 mM, pH 7.4) of foldamers **5b** and **5c** (1:1) in DOPC vesicles. Spectrum referenced to KF at −125.300 ppm.

To confirm that these differences in δ(CF_3_) are not due to variations between SUV populations, a 1:1 mixture of the diastereomeric pair **5b** and **5c** was prepared and included in SUVs. The resulting ^19^F NMR spectrum (Figure 8) shows two overlapping broad peaks which are in approximately the same position as in the ^19^F NMR spectra of the individual foldamers in SUVs. Line fitting analysis of the two overlapping peaks gives a 1:1 ratio, confirming that these foldamers have similar propensities to be incorporated into SUVs (Figure S18).

The observation of a single broad signal for each of these tetrameric Aib foldamers in SUV membranes, despite the inner and outer leaflets forming distinct bilayer environments (Figure 7a), could have two possible explanations. If exchange of the ^19^F-labeled foldamers between leaflets is fast, then a single signal may be observed.^36^ Alternatively, if exchange is slow, then the chemical shifts may be coincident.^18^ To obtain more insights, the shift reagent Pr(III) was used. Addition of Pr(III) (2 mM) to blank DOPC SUVs shifted the outer leaflet phospholipid ^31^P NMR signals to produce two resonances, one each for inner and outer leaflets (Figure S17).^37^ A similar titration of Pr(III) (up to 5 mM) into DOPC SUVs containing **5b** showed that the CF_3_^19^F resonances from nonincorporated **5b** (at −81.2 ppm) and added fluoride ions broadened and disappeared, but the broad CF_3_ resonance from membrane-embedded **5b** did not split or move (Figure S16). This confirms that the foldamer has embedded in the membrane but the absence of any splitting or shifting prevents further conclusive analysis. Nonetheless, previous computational and linear dichroism studies on similar Aib tetramers suggest that **6** will be buried in the hydrophobic region of the bilayer with its helical axis oriented parallel to the bilayer surface.^8b^

Because the opposite chirality of the TFEA reporter is available, the effect of phospholipid chirality on foldamer conformation could be assessed. ssNMR studies on foldamers with the Fib reporter had suggested that the effect of bilayer chirality on h.e. is small,^6a^ but enantiomers of a fluorescently labeled foldamer had shown a clear spectroscopic difference, although the magnitude of induction by the bilayer could not be quantified.^9^ Azido-capped Aib tetramer **7** (Scheme 1), the enantiomer of **6**, was prepared and incorporated into DOPC SUVs. The respective NMR spectra showed broad peaks at −82.931 ppm for **6** in DOPC SUVs and –82.967 ppm for **7** in DOPC SUVs, a difference of only +36 ppb (Figure S19). This is much less than the difference between the Phe-capped pair **5b** and **5c** in DOPC SUVs (222 ppb) and is similar to the standard error (SE) for these δ(CF_3_) measurements (SE = 21 ppb, calculated from different preparations of **6** in DOPC SUVs). This suggests that the magnitude of *M* helix induction by natural DOPC on these foldamers is small, corresponding to an h.e._0_ between 0 and –0.08 and much less than the effect of a chiral controller at the N-terminus.

## Conclusions

The (*R*)-TFEA group can report on changes in the ratio of *P* helical to *M* helical conformations of Aib foldamers both in organic solvents and the membranes of phospholipid vesicles. The isosteric relationship between CF_3_ and methyl did not prevent this reporter from perturbing foldamer helical excess (h.e.), but (*R*)-TFEA still proved to be an effective reporter of h.e. in both environments. X-ray crystallography showed that helical induction from (*R*)-TFEA at the C-terminus did not overcome the preference of a strong *M*-helix inducer, d-αMeVal, although it could compete against the relatively weak controller l-Phe.

Variable temperature ^19^F NMR spectroscopy of Aib tetramers **5a-c**, **5g-f** allowed the measured chemical shifts of the CF_3_ resonances of **5a-g**, **6** in organic solvents to be fitted to a simple model that correlated chemical shift with the reported helical preference of each N-terminal residue. This model confirmed that the (*R*)-TFEA reporter has a relatively weak bias for *P* helix over *M* helix (*P*/*M* ∼ 4). Similar chiral NMR reporters should also be effective provided that their chiral induction is less or similar to that of (*R*)-TFEA; stronger preferences will result in a loss of response to increases in one or other helical conformer.

Because this reporter provided conformational information from within the bilayers of intact phospholipid vesicles, it allowed us to verify that conformational preference from solution is maintained in phospholipid bilayers.^6a,6b,8a,9^ Both helical sense and differences in magnitude were reflected in the position of the broadened CF_3_ resonance. The respective resonances in bilayers from enantiomeric foldamers **6** (with (*R*)-TFEA) and **7** (with (*S*)-TFEA) also revealed that the magnitude of helical induction by natural (*R*)-phospholipids was not significant.^9^ The resonances from the (*R*)-TFEA reporter were sufficiently narrow that mixtures of diastereomeric foldamers in DOPC SUVs gave resolvable signals, which establishes a pathway to real-time NMR studies of *P*/*M* switching of Aib foldamer conformation induced by external stimuli.

The observation of resolvable ^19^F NMR signals from intact SUVs has allowed insight into important processes, including membrane partitioning and interactions with added ions in solution, that are not easily observable by ssNMR spectroscopy. Intact SUVs are more relevant to the structure of intact cells as they have enclosed volumes with defined chemical environments and may permit better mimics of GPCR function to be developed. For example, the use of SUVs may permit membrane-spanning foldamers undergoing conformational change to have dynamic interactions with encapsulated soluble reagents, producing outcomes that can be studied by the full suite of multinuclear NMR spectroscopic techniques.

## Supporting Information Available

Experimental section and synthetic overview for compounds **5a–g**, **6**, and **7**; FT-IR measurements; CD measurements; data modeling; and crystal data and structure refinement (PDF)
