# Supplementary material for: A Chiral 19F NMR Reporter of Foldamer Conformation in Bilayers
Source: J Am Chem Soc. 2022 Nov 15;144(47):21648–57. doi: 10.1021/jacs.2c09103 (PMC9716558; doi:10.1021/jacs.2c09103)
Supplement: Supplementary file 1 — ja2c09103_si_001.pdf [file ja2c09103_si_001.pdf]

Supporting Information for

A Chiral ^{19}F NMR Reporter of Foldamer Conformation in Bilayers

Siyuan Wang,[1,2] Flavio della Sala,[1,2] Matthew J. Cliff,[2] George F. S. Whitehead,[1] Iñigo J. Vitórica-Yrezábal,[1] and Simon J. Webb[1,2]*

1. *Department of Chemistry, University of Manchester, Oxford Road, Manchester M13 9PL, United Kingdom. E-mail: S.Webb@manchester.ac.uk; Tel: +44(0)-161-306-4448*
2. *Manchester Institute of Biotechnology, University of Manchester, 131 Princess St, Manchester M1 7DN, United Kingdom.*

TABLE OF CONTENTS

1. General experimental procedures **S4**
1.1 Abbreviations S5
2. Synthetic procedures **S6**
2.1 General experimental procedures S6
2.2 Cbz(Gly)Aib$_4$(*R*-TFEA) (**5a**) S6
2.3 Cbz(L-Phe)Aib$_4$(*R*-TFEA) (**5b**) S7
2.4 Cbz(D-Phe)Aib$_4$(*R*-TFEA) (**5c**) S7
2.5 Cbz(L-αMeVal)Aib$_4$(*R*-TFEA) (**5d**) S8
2.6 Cbz(D-αMeVal)Aib$_4$(*R*-TFEA) (**5e**) S9
2.7 Cbz(L-αMeVal)$_2$Aib$_4$(*R*-TFEA) (**5f**) S9
2.8 Cbz(D-αMeVal)$_2$Aib$_4$(*R*-TFEA) (**5g**) S10
2.9 N$_3$Aib$_4$(*R*-TFEA) (**6**) S11
2.10 N$_3$Aib$_4$(*S*-TFEA) (**7**) S11
3. FT-IR spectroscopy **S12**
3.1 Foldamer **6** S12
3.2 Foldamer **5a** S12
4. Circular dichroism spectroscopy **S13**
4.1 Spectra in acetonitrile S13
4.2 Spectra in methanol S13
5. Analysis of foldamers by NMR spectroscopy in organic solvents **S14**
5.1 ^{1}H NMR spectroscopy in CD_3CN and CD_3OD S14
5.2 ^{19}F NMR spectroscopy in CD_3CN and CD_3OD S16
5.3 VT-NMR spectra S18
6. Modelling of ^{19}F chemical shift in organic solvents **S19**
7. Analysis of foldamers in membranes **S23**
7.1 Preparation of small unilamellar vesicles (SUVs) by sonication S23
7.2 Preparation of SUVs containing **5b** and **5c** (1:1 ratio) S23
7.3 Dynamic light scattering (DLS) analysis S23
7.4 ^{19}F NMR experiment for analysis of foldamers in membranes S23
7.5 ^{19}F DOSY NMR S24
7.6 Purification by size exclusion chromatography (SEC) S25
7.7 Titration with $PrCl_3$ S26
7.8 Line fitting analysis of 1:1 mixture of **5b** and **5c** incorporated in DOPC SUVs S28
7.9 ^{19}F NMR spectra of foldamers **6** and **7** incorporated in DOPC SUVs S29
8. Changing foldamer loading in SUVs **S30**
9. Quantification of foldamer solubility and self-association in $CHCl_3$ **S31**
9.1 Experimental procedure (dilution) S31
9.2 Theory S31
9.3 Fitting S31
10. Line shape analysis of VT NMR data **S32**
10.1 Compound **5a** (Gly) S33
10.2 Compound **5b** (L-Phe) S34
11. Eyring plots **S35**
12. Crystal data and structure refinement **S37**
12.1 Crystallographic information for compound **5b**, **5d** and **5e** S37
12.2 Crystallographic information for compound **6** S38
13. NMR spectra of new compounds **S39**
13.1 Cbz(Gly)Aib$_4$(*R*-TFEA) (**5a**) S39
13.2 Cbz(L-Phe)Aib$_4$(*R*-TFEA) (**5b**) S41
13.3 Cbz(D-Phe)Aib$_4$(*R*-TFEA) (**5c**) S43
13.4 Cbz(L-αMeVal)Aib$_4$(*R*-TFEA) (**5d**) S45
13.5 Cbz(D-αMeVal)Aib$_4$(*R*-TFEA) (**5e**) S47
13.6 Cbz(L-αMeVal)$_2$Aib$_4$(*R*-TFEA) (**5f**) S49
13.7 Cbz(D-αMeVal)$_2$Aib$_4$(*R*-TFEA) (**5g**) S51
13.8 N$_3$Aib$_4$(*R*-TFEA) (**6**) S53

13.9 N_3Aib_4(*S*-TFEA) (**7**) S55
14. References **S57**

1. General experimental procedures

Unless stated otherwise, chemicals were purchased from Sigma-Aldrich Co. Ltd., Dorset, UK. D- and L-αMeVal were purchased from Fluorochem, Derbyshire. Anhydrous THF was obtained by drying with activated 4 Å molecular sieves. Anhydrous acetonitrile and *N*,*N*-dimethylformamide (DMF) were purchased from Sigma-Aldrich. Petroleum ether refers to the fraction of light petroleum ether boiling between 40 and 60 °C. Dichloromethane and toluene were dried over molecular sieves 4 Å under a nitrogen atmosphere. All other solvents and commercially available reagents were used as received without further purification. Foldamer starting materials were synthesised following the procedures referenced in Section 2.1.

All reactions were carried out using oven-dried glassware under an atmosphere of nitrogen using standard anhydrous techniques. All synthesised products were dried first on a rotary evaporator followed by further drying under high vacuum to remove any residual solvent. Flash chromatography was performed on silica gel (Merck 60H, 40-60 nm, 230–300 mesh). Analytical thin layer chromatography (TLC) was performed on Macherey Nagel alugram SIL G/UV254 TLC sheets and TLC plates were visualised by UV irradiation (254 nm), a ninhydrin stain or a potassium permanganate stain where appropriate.

NMR spectra were recorded in deuterated solvents using either Brüker AVANCE 400 MHz or Brüker AVANCE 500 MHz spectrometers. Chemical shifts are quoted in parts per million (ppm) and coupling constants (*J*) are quoted in Hz to the nearest 0.5 Hz. ^{1}H NMR spectra were referenced to the residual deuterated solvent peak ($CDCl_3$: 7.27; CD_3OD: 3.31; CD_3CN: 1.94 ppm).[S1] ^{13}C NMR spectra were referenced to the resonance of the solvent ($CDCl_3$: 77.2, CD_3OD: 49.0, CD_3CN: 118.3 ppm).[S1] ^{19}F spectra were referenced to an added standard that was either in the sample or in an coaxial tube (C_6F_6 at –164.38 in CD_3CN or –165.37 ppm in CD_3OD;[S2] KF at –125.3 ppm).[S3] Multiplicities are reported as s (singlet), d (doublet), t (triplet), q (quartet), m (multiplet), broad peak (b) or some combination of these, where appropriate. Assignments of the peaks were performed by analysis of chemical shifts, coupling constants, ^{1}H-^{1}H COSY data, and ^{1}H-^{13}C HSQC data.

Reversed-phase HPLC purification of foldamers (when required) was performed on an Agilent 1100 series system with a semi-preparative C18 column Agilent Eclipse XDB-C18, 5 µm, 9.4 mm × 250 mm. Sonication of phospholipid suspensions was performed with a bath sonicator.

Low- and high-resolution mass spectra were recorded by staff at the University of Manchester. Electrospray (ES) spectra were recorded on an Agilent 6530 LC/MS. High resolution mass spectra (HRMS) were recorded on a Thermo Q-Exactive and are accurate to ±0.001 Da.

Size exclusion chromatography (SEC; gel permeation chromatography (GPC)) was performed on PD-10 SEC columns (Sephadex™ G-25).

CD spectra were recorded on an Applied Photophysics Chirascan-plus circular dichroism spectrometer. A cuvette with 0.1 mm path length was used for all samples. The wavelength range was from 190 to 280 nm. The spectra were recorded with 0.1 nm wavelength increments with 0.5 s per scan.

FT-IR spectra were recorded as neat samples on a Brüker Alpha-P ATR FTIR (Diamond-ATR) instrument.

Dynamic light scattering (DLS) analysis was performed on a Malvern Zetasizer Nano-S instrument.

1.1 Abbreviations

Aib: α-amino-*iso*-butyric acid;

αMeVal: α-methylvaline;

Boc: *tert*-butyloxycarbonyl;

Cbz: carboxybenzyl;

CD: circular dichroism;

DCM: dichloromethane;

DLS: dynamic light scattering;

DOPC: 1,2-dioleoyl-*sn*-glycero-3-phosphocholine;

DOSY: diffusion-ordered spectroscopy;

DIPEA: *N*,*N*-diisopropylethylamine;

EDC·HCl: *N*-Ethyl-*N′*-(3-dimethylaminopropyl)-carbodiimide hydrochloride;

e.e.: enantiomeric excess;

Gly: Glycine;

HATU: hexafluorophosphate azabenzotriazole tetramethyl uronium;

h.e.: helical excess;

HPLC: high-performance liquid chromatography;

FT-IR: Fourier-transform infrared spectroscopy

IS: internal standard

MOPS: 3-(*N*-morpholino)propanesulfonic acid;

NMR: nuclear magnetic resonance;

Phe: phenylalanine;

ppm: parts per million;

ss-NMR: solid-state nuclear magnetic resonance;

(*R* or *S*)-TFEA: (*R* or *S*)-1-(trifluoromethyl)ethylamine;

TFFH: tetramethylfluoroformamidinium hexafluorophosphate;

VT-NMR: variable temperature nuclear magnetic resonance.

2. Synthetic procedures

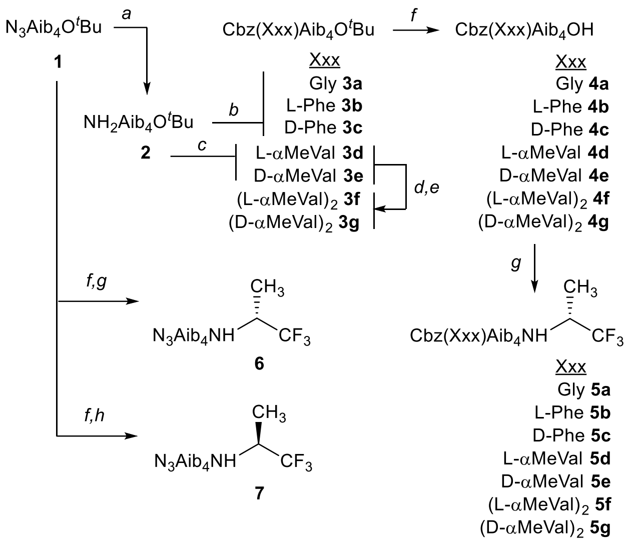

Scheme S1 (a): H_2/Pd(C), EtOH, rt. (b): Cbz(Gly)OH or Cbz(D/L-Phe)OH, EDC·HCl, HOBt, DIPEA, CH_2Cl_2, rt; (c): *i.* Cbz(D/L-αMeVal)OH, cyanuric fluoride, pyridine, CH_2Cl_2, rt; *ii.* 2, CH_2Cl_2, DIPEA rt. (d): **3d**, **3e**, H_2/Pd(C), MeOH, rt. (e) *i.* Cbz(D/L-αMeVal)OH, tetramethylfluoroformamidinium hexafluorophosphate, pyridine, CH_2Cl_2, rt; *ii.* DIPEA, CH_2Cl_2, rt. (f): CF_3CO_2H, CH_2Cl_2, rt. (g): (*R*)-2-Amino-1,1,1-trifluoropropane·HCl, DIPEA, HATU, rt. (h): (*S*)-2-Amino-1,1,1-trifluoropropane·HCl, DIPEA, HATU, rt.

2.1 General experimental procedures

$N_3Aib_4O^tBu$ (**1**),[S4] $NH_2Aib_4O^tBu$ (**2**),[S4] N_3Aib_4OH (**S1**),[S4] Cbz(Xxx)Aib_4O^tBu (**3a**–**g**)[S5–8] and Cbz(Xxx)Aib_4OH (**4a**–**g**)[S5–8] were synthesized according to reported procedures.

2.2 Cbz(Gly)Aib_4(*R*-TFEA) (5a)

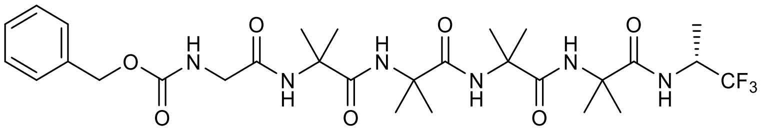

Cbz(Gly)Aib_4OH (**4a**, 17.7 mg, 0.03 mmol), (*R*)-2-amino-1,1,1-trifluoropropane hydrochloride (10 mg, 0.07 mmol), DIPEA (20 μL, 0.11 mmol), and HATU (12 mg, 0.03 mmol), were dissolved in dry CH_2Cl_2 (3 mL). The reaction mixture was stirred at room temperature for 3 days. The reaction mixture was diluted with CH_2Cl_2 (10 mL) and washed with saturated $NaHCO_3$ (10 mL). The aqueous phase was extracted with CH_2Cl_2 (2 × 10 mL). The organic layers were combined, dried over $MgSO_4$ then filtered. The solvent was removed from the filtrate under reduced pressure. The residue, containing the crude product, was purified by flash chromatography (SiO_2, EtOH/hexane, 2:1) to afford foldamer **5a** as white solid (13 mg, 61%).

^1H NMR (400 MHz, CD_3OD): δ 7.81 (s, 2H, 2 × NH), 7.37–7.29 (m, 5H, 5 × H_{Ar}), 5.15–5.07 (m, 2H, CH_2Ph), 4.61–4.52 (m, 1H, C*H*CH_3CF_3), 3.80–3.66 (m, 2H, CH_2), 1.49 (s, 3H, CH_3), 1.48 (s, 3H, CH_3), 1.42 (s, 3H, CH_3), 1.41 (s, 3H, CH_3), 1.40 (s, 9H, 3 × CH_3), 1.38 (s, 6H, 2 × CH_3).

^{19}F NMR (376 MHz, CD_3OD): δ –78.688 (s, 3F, CF_3).

^{13}C NMR (101 MHz, CD_3OD): δ 177.8, 177.34, 177.26, 176.9, 176.5, 159.4 (CO), 138.2, 129.5, 129.1, 128.7 (C_{Ar}), 127.1 (q, *J* = 281 Hz, CF_3), 67.8 (CH_2), 58.2, 57.9, 57.7, 57.6 (C), 47.9 (q, *J* = 32 Hz, $CHCF_3$), 45.2 (CH_2), 27.3, 26.6, 26.2, 25.9, 24.4, 24.3, 24.2, 24.0 (CH_3).

HRMS (ES^+, MeOH): m/z calculated for $C_{29}H_{43}O_7N_6F_3Na$ $[M+Na]^+$ 667.3019, found 667.3038

2.3 Cbz(L-Phe)Aib$_4$(*R*-TFEA) (5b)

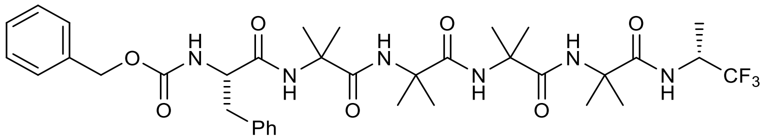

Cbz(L-Phe)Aib$_4$OH (**4b**, 85 mg, 0.13 mmol), (*R*)-2-amino-1,1,1-trifluoropropane hydrochloride (40 mg, 0.27 mmol), DIPEA (70 μL, 0.40 mmol), and HATU (51 mg, 0.13 mmol), were dissolved in dry CH_2Cl_2 (3 mL). The reaction mixture was stirred at room temperature for 3 days. The reaction mixture was diluted with CH_2Cl_2 (10 mL) and washed with saturated $NaHCO_3$ (10 mL). The aqueous phase was extracted with CH_2Cl_2 (2 × 10 mL). The organic layers were combined, dried over $MgSO_4$ then filtered. The solvent was removed from the filtrate under reduced pressure. The residue, containing the crude product, was purified by flash chromatography (SiO_2, EtOH/hexane, 2:1) to afford foldamer **5b** as white solid (40 mg, 42%).

^{1}H NMR (400 MHz, $CDCl_3$): δ 7.56 (d, 1H, *J* = 3.3 Hz, NH), 7.44 (s, 1H, NH), 7.41 (s, 1H, NH), 7.38–7.30 (m, 8H, H_{Ar}), 7.20–7.17 (m, 2H, H_{Ar}), 7.00 (bs, 1H, NH), 6.20 (bs, 1H, NH), 6.06 (bs, 1H, NH), 5.23 (d, *J* = 3.3 Hz, 1H, NH), 5.15 (d, *J* = 12 Hz, 1H, H_A, $CH_AH_BPh_{Cbz}$), 5.04 (d, *J* = 12 Hz, 1H, H_B, $CH_AH_BPh_{Cbz}$), 4.71–4.59 (m, 1H, $CHCF_3$), 4.20–4.13 (m, 1H, CH_2Ph), 3.17 (dd, *J* = 14, 5.7 Hz, 1H, H_A, CH_AH_BPh), 2.97 (dd, *J* = 14.1, 8.6 Hz, 1H, H_A, CH_AH_BPh),1.55 (s, 3H, CH_3), 1.49 (s, 3H, CH_3), 1.48 (s, 3H, CH_3), 1.46 (s, 3H, CH_3), 1.43 (s, 3H, CH_3), 1.40 (s, 3H, CH_3), 1.38 (s, 3H, CH_3), 1.37 (s, 3H, CH_3), 1.17 (s, 3H, CH_3).

^{19}F NMR (470 MHz, CD_3OD): δ –78.477 (s, 3F, CF_3).

^{13}C NMR (101 MHz, $CDCl_3$): δ 176.1, 175.0, 174.3, 173.7, 171.7 (CO), 157.1, 136.0, 135.8, 129.23, 129.16, 128.8, 128.7, 128.3, 127.7 (C_{Ar}), 125.9 (q, *J* = 282 Hz, CF_3), 67.7, 57.9, 57.0, 56.98, 56.9, 56.8 (C), 46.6 (q, *J* = 31 Hz, $\underline{C}HCF_3$), 36.8, 27.1, 26.4, 26.1, 24.2, 23.9, 23.7, 13.5 (CH_3).

HRMS (ES$^+$, MeOH): m/z calculated for $C_{36}H_{49}F_3N_6O_7Na$, $[M+Na]^+$ 757.3513, found 757.3507.

2.4 Cbz(D-Phe)Aib$_4$(*R*-TFEA) (5c)

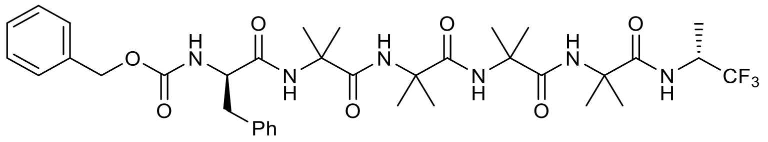

Cbz(D-Phe)Aib$_4$OH (**4c**, 101 mg, 0.145 mmol), (*R*)-2-amino-1,1,1-trifluoropropane hydrochloride (60 mg, 0.40 mmol), DIPEA (80 μL, 0.45 mmol), and HATU (60 mg, 0.15 mmol), were dissolved in dry CH_2Cl_2 (3 mL). The reaction mixture was stirred at room temperature for 3 days. The reaction mixture was diluted with CH_2Cl_2 (10 mL) and washed with saturated $NaHCO_3$ (10 mL). The aqueous phase was extracted with CH_2Cl_2 (2 × 10 mL). The organic layers were combined, dried over $MgSO_4$ then filtered. The solvent was removed from the filtrate under reduced pressure. The residue, containing the crude product, was purified by flash chromatography (SiO_2, EtOH/hexane, 2:1) to afford foldamer **5c** as white solid (52 mg, 55%).

^{1}H NMR (400 MHz, $CDCl_3$): δ 7.54 (d, 1H, NH), 7.49 (s, 1H, NH), 7.42 (s, 1H, NH), 7.39–7.26 (m, 8H, H_{Ar}), 7.20–7.15 (m, 2H, H_{Ar}), 7.04 (bs, 1H, NH), 6.90 (bs, 1H, NH), 6.32 (bs, 1H, NH), 5.52 (d, *J* = 5.0 Hz, 1H, NH), 5.16 (d, *J* = 12 Hz, 1H, H_A, $CH_AH_BPh_{Cbz}$), 5.08 (d, *J* = 12 Hz, 1H, H_B, $CH_AH_BPh_{Cbz}$), 4.70–4.54 (m, 1H, *J* = 7.6 Hz, $CHCF_3$), 3.19 (dd, *J* = 14, 6.4 Hz, 1H, H_A, $CH_AH_BPh_{Cbz}$), 3.05 (dd, *J* = 14, 8.6 Hz, 1H, H_B, $CH_AH_BPh_{Cbz}$), 1.54 (s, 3H, CH_3), 1.49 (s, 3H, CH_3), 1.46 (s, 3H, CH_3), 1.44 (s, 6H, 2 × CH_3), 1.38 (s, 3H, CH_3), 1.37 (s, 3H, CH_3), 1.30 (s, 3H, CH_3), 1.28 (s, 3H, CH_3).

^{19}F NMR (376 MHz, CD_3OD): δ –78.857 (s, 3F, CF_3).

^{13}C NMR (101 MHz, $CDCl_3$): δ 176.1, 175.2, 174.4, 174.0, 171.7 (CO), 156.7, 136.5, 136.1, 129.4, 129.0, 128.8, 128.6, 128.1, 127.4 (C_{Ar}), 125.9 (q, *J* = 283 Hz, CF_3) 67.4, 57.6, 57.0, 56.95, 56.92, 56.89 (C), 46.6 (q, *J* = 32 Hz, *C*HCF_3), 36.4, 27.4, 26.7, 26.5, 23.8, 23.6, 23.4, 23.3, 13.5 (CH_3).

HRMS (ES$^+$, MeOH): m/z calculated for $C_{36}H_{49}F_3N_6O_7Na$, $[M+Na]^+$ 757.3513, found 757.3507.

2.5 Cbz(L-αMeVal)Aib$_4$(*R*-TFEA) (5d)

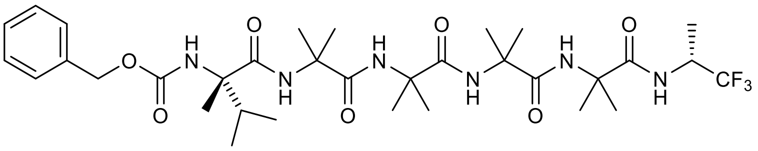

Cbz(L-αMeVal)Aib$_4$OH (**4d**, 21 mg, 0.035 mmol), (*R*)-2-amino-1,1,1-trifluoropropane hydrochloride (11 mg, 0.07 mmol), DIPEA (60 μL, 0.11 mmol) and HATU (15 mg, 0.04 mmol) were dissolved in dry CH_2Cl_2 (3 mL). The reaction mixture was stirred at room temperature for 3 days. The reaction mixture was diluted with CH_2Cl_2 (5 mL) and washed with saturated $NaHCO_3$ (5 mL). The aqueous phase was extracted with CH_2Cl_2 (2 × 5 mL). The organic layers were combined, dried over $MgSO_4$ then filtered. The solvent was removed from the filtrate under reduced pressure. The residue, containing the crude product, was purified by semi-preparative HPLC on an Agilent 1100 series HPLC equipped with a semi-preparative Agilent Eclipse XDB-C18 column, (C18, 5 µm, 9.4 mm × 250 mm), a flow rate of 1 mL/min and the eluent mixture below. The product containing fractions were combined and the organic solvent was removed under reduced pressure. The remaining aqueous solution was freeze-dried to give foldamer **5d** as white solid (12 mg, 50%).

Time/min	%MeCN	%Water
0	30	70
10	70	30
20	80	20
30	95	5
50	95	5

^{1}H NMR (400 MHz, $CDCl_3$): δ 7.68 (s, 1H, NH), 7.57 (d, *J* = 9.0 Hz, 1H, NH), 7.50 (s, 1H, NH), 7.46 (s, 1H, NH), 7.41–7.30 (m, 5H, H_{Ar}), 6.38 (s, 1H, NH), 5.47 (d, *J* = 14.8 Hz, 1H, NH), 5.13 (d, *J* = 12 Hz, 1H, H_A, $CH_AH_BPh_{Cbz}$), 4.96 (d, *J* = 12 Hz, 1H, H_B, $CH_AH_BPh_{Cbz}$). 4.64 (m, 1H, C*H*CH_3CF_3), 1.96–1.86 (m, 1H, C*H*$(CH_3)_2$), 1.55 (s, 3H, CH_3), 1.47 (s, 6H, 2 × CH_3), 1.46–1.42 (m, 9H, 3 × CH_3), 1.42–1.38 (m, 9H, 3 × CH_3), 1.17 (s, 3H, CH_3), 0.98 (d, *J* = 6.5 Hz, 3H, CH(C*H*$_3$)), 0.94 (d, *J* = 6.6 Hz, 3H, CH(C*H*$_3$).

^{19}F NMR (376 MHz, CD_3OD): δ –78.866 (s, 3F, CF_3).

^{13}C NMR (101 MHz, $CDCl_3$): δ 176.0, 175.4, 174.4, 173.9, 172.8, 156.3, 136.1, 128.9, 128.8, 128.7, 128.4, 128.3, 126.0 (q, *J* = 281 Hz, CF_3), 67.6, 63.3, 57.0, 56.93, 56.91, 56.7, 46.6 (q, *J* = 31 Hz, *C*HCF_3), 35.8, 28.0, 27.3, 27.2, 27.1, 23.1, 23.0, 22.9, 22.7, 17.6, 17.4, 17.3, 13.5.

HRMS (ES$^+$, MeOH): m/z calculated for $C_{33}H_{51}F_3N_6O_7Na$, $[M+Na]^+$ 723.3669, found 723.3687.

2.6 Cbz(D-αMeVal)Aib$_4$(*R*-TFEA) (5e)

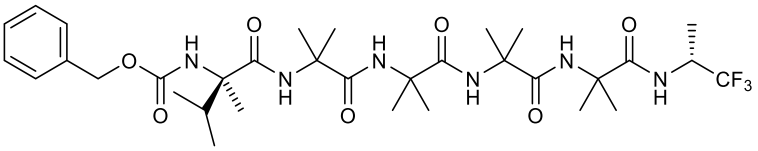

Cbz(D-αMeVal)Aib$_4$OH (**4e**, 340 mg, 0.56 mmol), (*R*)-2-amino-1,1,1-trifluoropropane hydrochloride (170 mg, 1.14 mmol), DIPEA (1.68 mL, 1.68 mmol) and HATU (220 mg, 0.58 mmol), were dissolved in dry CH_2Cl_2 (10 mL). The reaction mixture was stirred at room temperature for 3 days. The reaction mixture was diluted with CH_2Cl_2 (5 mL) and washed with saturated $NaHCO_3$ (5 mL). The aqueous phase was extracted with CH_2Cl_2 (2 × 5 mL). The organic layers were combined, dried over $MgSO_4$ then filtered. The solvent was removed from the filtrate under reduced pressure. The residue, containing the crude product, was purified by semi-preparative HPLC on an Agilent 1100 series HPLC equipped with a semi-preparative Agilent Eclipse XDB-C18 column, (C18, 5 µm, 9.4 mm × 250 mm), a flow rate of 1 mL/min and the eluent mixture below. The product containing fractions were combined, and the organic solvent was removed under reduced pressure. The remaining aqueous solution was freeze-dried to give foldamer **5e** as white solid (274 mg, 70%).

Time/min	%MeCN	%Water
0	30	70
10	70	30
20	80	20
30	95	5
50	95	5

^{1}H NMR (500 MHz, $CDCl_3$) δ 7.69 (d, *J* = 9.2 Hz, 1H, NH), 7.61 (s, 1H, NH), 7.44 (s, 1H, NH), 7.35 (m, 6H, 5 x H_{Ar}, NH), 6.38 (s, 1H, NH), 5.43 (s, 1H, NH), 5.13 (d, *J* = 12 Hz, 1H, H_A, $CH_AH_BPh_{Cbz}$), 5.06 (d, *J* = 12 Hz, 1H, H_B, $CH_AH_BPh_{Cbz}$), 4.66–4.55 (m, 1H, C*H̲*CH_3CF_3), 2.05 (m, 1H, C*H̲*$(CH_3)_2$), 1.54 (s, 3H, CH_3), 1.50 (s, 3H, CH_3), 1.46 (s, 3H, CH_3), 1.45– 1.42 (m, 9H, 3 × CH_3), 1.41 (m, 6H, 2 × CH_3), 1.37 (d, *J* = 7.1 Hz, 3H, CHC*H̲*$_3CF_3$), 1.26 (s, 3H, CH_3), 0.97 (d, *J* = 6.7 Hz, 3H, CH(C*H̲*$_3$)), 0.95 (d, *J* = 6.7 Hz, 3H, CH(C*H̲*$_3$)).

^{19}F NMR (376 MHz, CD_3OD): δ –78.402 (s, 3F, CF_3).

^{13}C NMR (126 MHz, $CDCl_3$): δ 175.9, 175.4, 174.2, 174.0, 173.3, 156.2, 136.1, 128.9, 128.8, 128.3, 125.9 (q, *J* = 282 Hz, CF_3), 67.6, 62.9, 57.1, 57.0, 56.9, 56.8, 46.6 (q, *J* = 31 Hz, *C̲*HCF_3), 35.2, 26.1, 25.8, 25.7, 25.6, 25.3, 24.7, 24.4, 24.3, 17.9, 17.5, 17.4, 13.6.

HRMS (ES$^+$, MeOH): m/z calculated for $C_{33}H_{51}F_3N_6O_7Na$, $[M+Na]^+$ 723.3669, found 723.3632.

2.7 Cbz(L-αMeVal)$_2$Aib$_4$(*R*-TFEA) (5f)

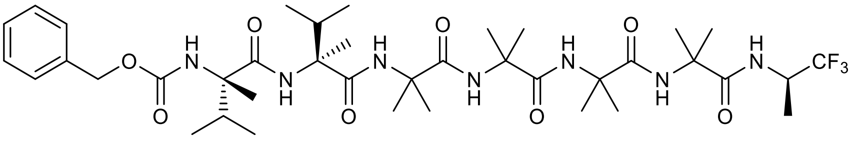

Cbz(L-αMeVal)$_2$Aib$_4$OH (**4f**, 150 mg, 0.21 mmol), (*R*)-2-amino-1,1,1-trifluoropropane hydrochloride (63 mg, 0.43 mmol), DIPEA (0.1 mL, 0.57 mmol) and HATU (80 mg, 0.21 mmol), were dissolved in dry CH_2Cl_2 (10 mL). The reaction mixture was stirred at room temperature for 3 days. The reaction mixture was diluted with CH_2Cl_2 (10 mL) and washed with saturated $NaHCO_3$ (10 mL). The aqueous phase was extracted with CH_2Cl_2 (2 × 10 mL). The organic layers were combined, dried over $MgSO_4$ then filtered. The solvent was removed

from the filtrate under reduced pressure. The residue, containing the crude product, was purified by flash chromatography (SiO_2, EtOAc/petroleum ether, 3:1) to afford foldamer **5f** as white solid (125 mg, 73%).

^{1}H NMR (400 MHz, $CDCl_3$): δ 7.75 (s, 1H, NH), 7.70 (s, 1H, NH), 7.57–7.53 (m, 3H, 3 × NH), 7.39–7.35 (m, 5H, 5 × H_{Ar}), 6.35 (s, 1H, NH), 5.27 (s, 1H, NH), 5.19 (d, *J* = 12.1 Hz, 1H, H_A, $CH_AH_BPh_{Cbz}$), 5.02 (d, *J* = 12.2 Hz, 1H, H_B, $CH_AH_BPh_{Cbz}$), 4.73–4.60 (m, 1H, C*H*CH_3CF_3), 1.90–1.81 (m, 1H, C*H*$(CH_3)_2$), 1.51–1.55 (m, 1H, C*H*$(CH_3)_2$), 1.58 (s, 3H, CH_3), 1.50 (s, 3H, CH_3), 1.48 (s, 3H, CH_3), 1.47 (s, 6H, 2 × CH_3), 1.45 (s, 3H, CH_3), 1.44 (s, 6H, 2 × CH_3), 1.43 (s, 3H, CH_3), 1.41 (d, *J* = 7.0 Hz, 3H, CHC*H*$_3CF_3$) , 1.40 (s, 3H, CH_3), 0.99 (d, *J* = 7.0 Hz, 3H, CH(C*H*$_3$)), 0.96 (d, *J* = 7.1 Hz, 3H, CH(C*H*$_3$), 0.78 (m, *J* = 6.4 Hz, 6H, 2 × CH(C*H*$_3$).

^{19}F NMR (376 MHz, CD_3OD): δ –78.944 (s, 3F, CF_3).

^{13}C NMR (101 MHz, $CDCl_3$): 176.1, 175.8, 175.0, 174.6, 172.41, 172.37, (CO), 156.3, 135.8, 128.91, 128.87, 128.7 (C_{Ar}), 126.1 (q, *J* = 285 Hz, CF_3), 67.9 (CH_2Ph), 63.6, 62.5, 57.0, 56.9 (C), 46.6 (q, *J* = 31 Hz, *C*HCF_3), 36.2, 35.9 (CH), 28.2, 27.5, 27.34, 27.30, 22.9, 22.8, 22.6, 18.20 (CH_3), 18.18 (CH), 17.4, 17.3, 17.2, 17.1, 13.6 (CH_3).

HRMS (ES^+, MeOH): m/z calculated for $C_{39}H_{62}O_8N_7F_3Na$ $[M+Na]^+$ 836.4510, found 836.4504.

2.8 Cbz(D-αMeVal)$_2$Aib$_4$(*R*-TFEA) (5g)

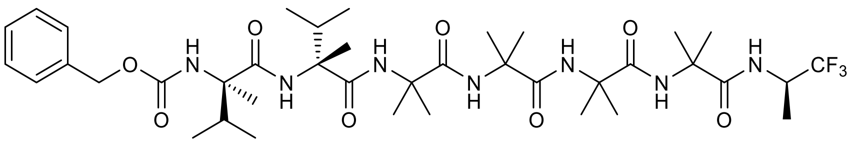

Cbz(L-αMeVal)$_2$Aib$_4$OH (**4g**, 90 mg, 0.13 mmol), (*R*)-2-amino-1,1,1-trifluoropropane hydrochloride (40 mg, 0.27 mmol), DIPEA (68 μL, 0.39 mmol) and HATU (50 mg, 0.13 mmol), were dissolved in dry CH_2Cl_2 (5 mL). The reaction mixture was stirred at room temperature for 3 days. The reaction mixture was diluted with CH_2Cl_2 (10 mL) and washed with saturated $NaHCO_3$ (10 mL). The aqueous phase was extracted with CH_2Cl_2 (2 × 10 mL). The organic layers were combined, dried over $MgSO_4$ then filtered. The solvent was removed from the filtrate under reduced pressure. The residue, containing the crude product, was purified by flash chromatography (SiO_2, EtOAc/petroleum ether, 3:1) to afford foldamer **5g** as white solid (64 mg, 60%).

^{1}H NMR (400 MHz, $CDCl_3$): δ 7.79 (d, *J* = 9.2 Hz, 1H, NH), 7.57 (s, 1H, NH), 7.38 (s, 1H, NH), 7.37–7.30 (m, 5H, H_{Ar}), 6.41 (s, 1H, NH), 5.67 (s, 1H, NH), 5.17 (d, *J* = 12 Hz, 1H, H_A, $CH_AH_BPh_{Cbz}$), 5.01 (d, *J* = 12 Hz, 1H, H_B, $CH_AH_BPh_{Cbz}$), 4.66–4.50 (m, 1H, C*H*CH_3CF_3), 1.94–1.82 (m, 1H, C*H*$(CH_3)_2$), 1.59 (s, 3H, CH_3), 1.56 -1.53 (m, 1H, C*H*$(CH_3)_2$), 1.50 (s, 3H, CH_3), 1.47 (s, 3H, CH_3), 1.46 (s, 9H, 3 × CH_3), 1.44 (s, 3H, CH_3), 1.42 (s, 3H, CH_3), 1.35 (d, *J* = 7.1 Hz, 3H, CH_3), 0.96 (m, 6H, 2 × CH(C*H*$_3$), 0.77 (m, 6H, 2 × CH(C*H*$_3$)).

^{19}F NMR (376 MHz, CD_3OD): δ –78.226 (s, 3F, CF_3).

^{13}C NMR (101 MHz, $CDCl_3$): δ 176.0, 175.9, 175.3, 174.4, 172.7, 172.5 (CO), 156.5, 136.0, 128.8, 128.7, 128.6 (C_{Ar}), 125.7 (q, *J* = 282 Hz, CF_3), 67.7 (CH_2Ph), 63.6, 62.4, 57.2, 57.0, 56.9, 56.8 (C), 46.5 (q, *J* = 32 Hz, *C*HCF_3), 35.9 (CH), 35.8, 27.7, 27.6, 27.2, 27.05, 26.99, 26.9, 24.0, 23.9, 23.0, 22.9, 18.2, 18.1 (CH_3), 17.6 (CH), 17.5, 17.3, 17.14, 17.07, 13.6 (CH_3).

HRMS (ES^+, MeOH): m/z calculated for $C_{39}H_{62}O_8N_7F_3Na$ $[M+Na]^+$ 836.4504, found 836.4517.

2.9 N_3Aib_4(*R*-TFEA) (6)

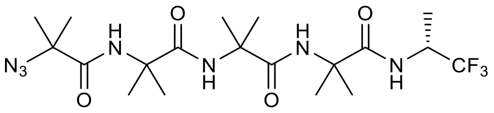

N_3Aib_4OH (**S1**, 42.3 mg, 0.11 mmol), (*R*)-2-amino-1,1,1-trifluoropropane hydrochloride (33.3 mg, 0.22 mmol), DIPEA (0.06 mL, 0.34 mmol), and HATU (41.8 mg, 0.11 mmol), were dissolved in dry CH_2Cl_2 (1.5 mL). The reaction mixture was stirred at room temperature for 3 days. The reaction mixture was diluted with CH_2Cl_2 (10 mL) and washed with saturated $NaHCO_3$ (10 mL). The aqueous phase was extracted with CH_2Cl_2 (2 × 10 mL). The organic layers were combined and dried over $MgSO_4$. The solvent was removed under reduced pressure. The crude product was purified by flash chromatography (SiO_2, hexane/ethyl acetate, 1:3) to afford foldamer **6** as white solid (25 mg, 48%).

^1H NMR (400 MHz, $CDCl_3$): δ 7.37 (d, 1H, *J* = 9.1, NH), 7.22 (bs, 1H, NH), 6.83 (bs, 1H, NH), 6.08 (bs, 1H, NH), 4.70–4.58 (m, 1H, CH), 1.54 (s, 3H, CH_3), 1.53 (s, 3H, CH_3), 1.49 (s, 3H, CH_3), 1.48 (s, 3H, CH_3), 1.48 (s, 3H, CH_3), 1.47 (s, 3H, CH_3), 1.45 (s, 3H, CH_3), 1.37 (s, 3H, CH_3), 1.35 (s, 3H, CH_3).

^{19}F NMR (376 MHz, CD_3OD): δ –78.716 (s, 3F, CF_3).

^{13}C NMR (101 MHz, $CDCl_3$): δ 175.2, 173.1, 173.0, 172.5 (C_{Ar}), 125.8 (q, *J* = 281 Hz, CF_3), 64.0, 57.1, 57.0, 56.8 (C), 46.5 (q, *J* = 31 Hz, $\underline{C}HCF_3$), 27.0, 26.8, 25.7, 24.4, 24.1, 23.8, 23.74, 23.71 (CH_3).

HRMS (ES^+, MeOH): m/z calculated for $C_{19}H_{32}F_3N_7O_4Na$ $[M+Na]^+$ 502.2366, found 502.2337.

2.10 N_3Aib_4(*S*-TFEA) (7)

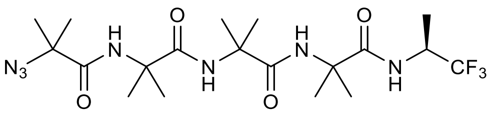

N_3Aib_4OH (**S1**, 107.7 mg, 0.28 mmol), (*S*)-2-amino-1,1,1-trifluoropropane hydrochloride (83 mg, 0.56 mmol), DIPEA (0.14 mL, 0.84 mmol), and HATU (106 mg, 0.28 mmol), were dissolved in dry CH_2Cl_2 (5 mL). The reaction mixture was stirred at room temperature for 3 days. The reaction mixture was diluted with CH_2Cl_2 (10 mL) and washed with saturated $NaHCO_3$ (10 mL). The aqueous phase was extracted with CH_2Cl_2 (2 × 10 mL). The organic layers were combined and dried over $MgSO_4$. The solvent was removed under reduced pressure. The crude product was purified by flash chromatography (SiO_2, hexane/ethyl acetate, 1:3) to afford foldamer **7** as white solid (52 mg, 40%).

^1H NMR (500 MHz, $CDCl_3$): δ 7.37 (d, 1H, *J* = 9.1, NH), 7.21 (bs, 1H, NH), 6.83 (bs, 1H, NH), 6.08 (bs, 1H, NH), 4.70–4.59 (m, 1H, CH), 1.55 (s, 3H, CH_3), 1.54 (s, 3H, CH_3), 1.53 (s, 3H, CH_3), 1.49 (s, 3H, CH_3), 1.48 (s, 3H, CH_3), 1.47 (s, 3H, CH_3), 1.45 (s, 3H, CH_3), 1.37 (s, 3H, CH_3), 1.36 (d, 3H, CH_3).

^{19}F NMR (470 MHz, $CDCl_3$): δ –77.4 (s, 3F, CF_3; no internal standard added).

^{13}C NMR (126 MHz, $CDCl_3$): δ 175.3, 173.1, 173.0, 172.5 (C_{Ar}), 125.5 (q, *J* = 305 Hz, CF_3), 64.2, 57.2, 57.1, 56.9 (C), 46.6 (q, *J* = 34 Hz, $\underline{C}HCF_3$), 27.2, 27.0, 26.9, 25.9, 24.5, 24.3, 23.91, 23.85 (CH_3).

HRMS (ES^+, MeOH): m/z calculated for $C_{19}H_{32}F_3N_7O_4Na$ $[M+Na]^+$ 502.2366, found 502.2360.

3. FT-IR spectroscopy

3.1 Foldamer 6

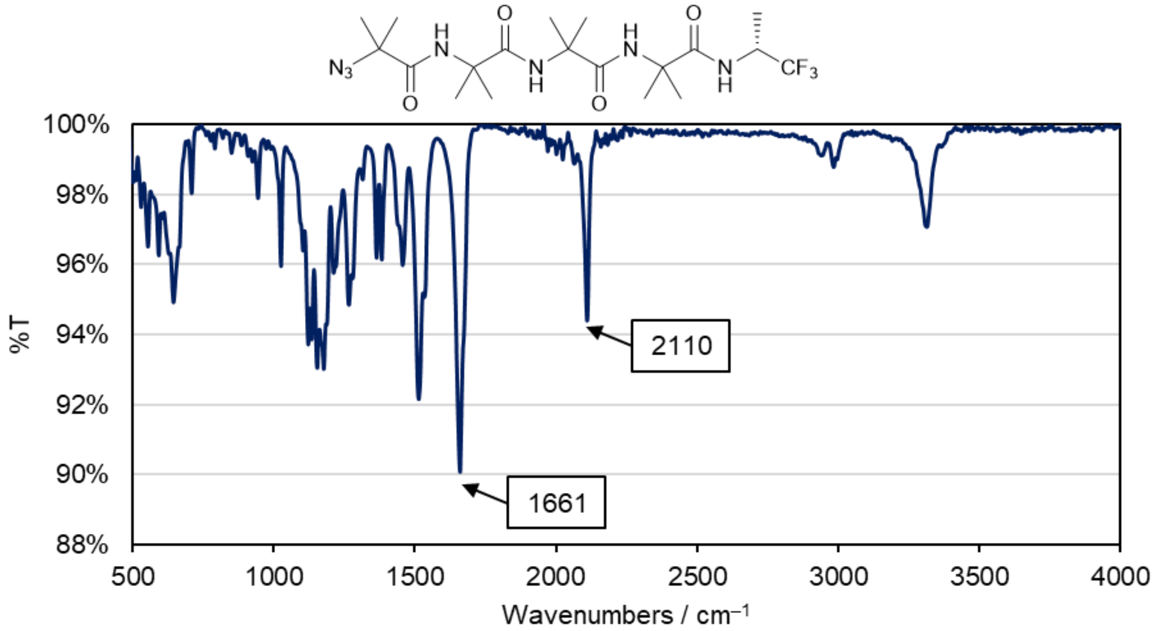

Figure S1 FT-IR spectra (neat sample) of foldamer **6**.

3.2 Foldamer 5a

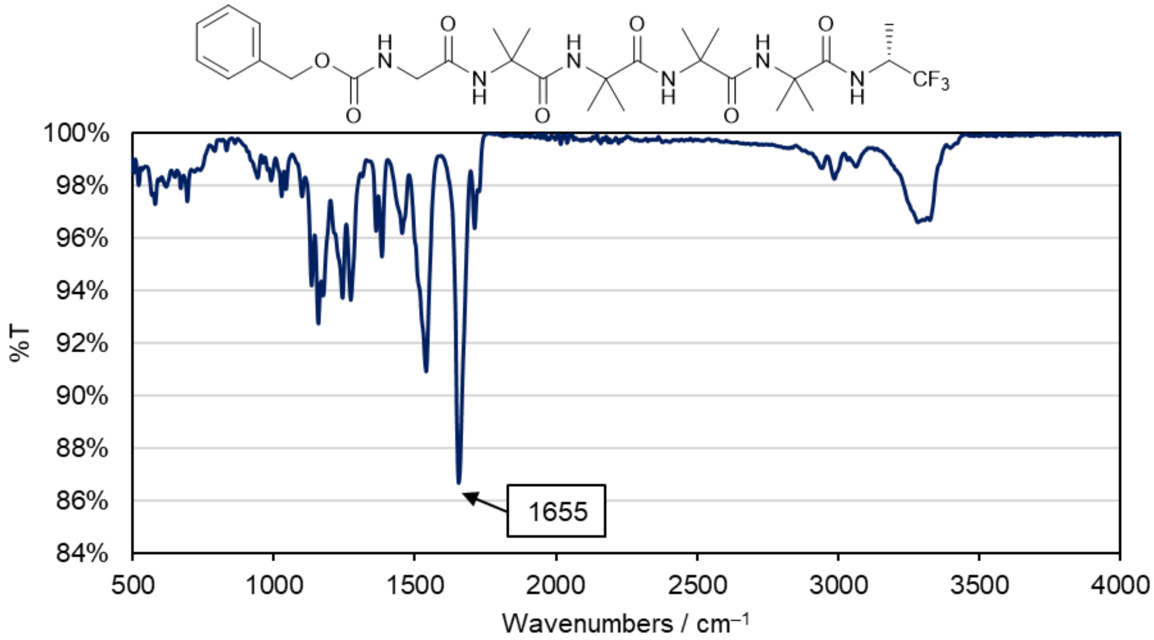

Figure S2 FT-IR spectra (neat sample) of foldamer **5a**.

4. Circular dichroism spectroscopy

Samples were prepared by dissolving each compound in either acetonitrile or methanol ([foldamer] = 1.36 mmol mL^{-1}). The solution was then loaded into a cuvette of 0.1 mm pathlength for measurement.

4.1 Spectra in acetonitrile

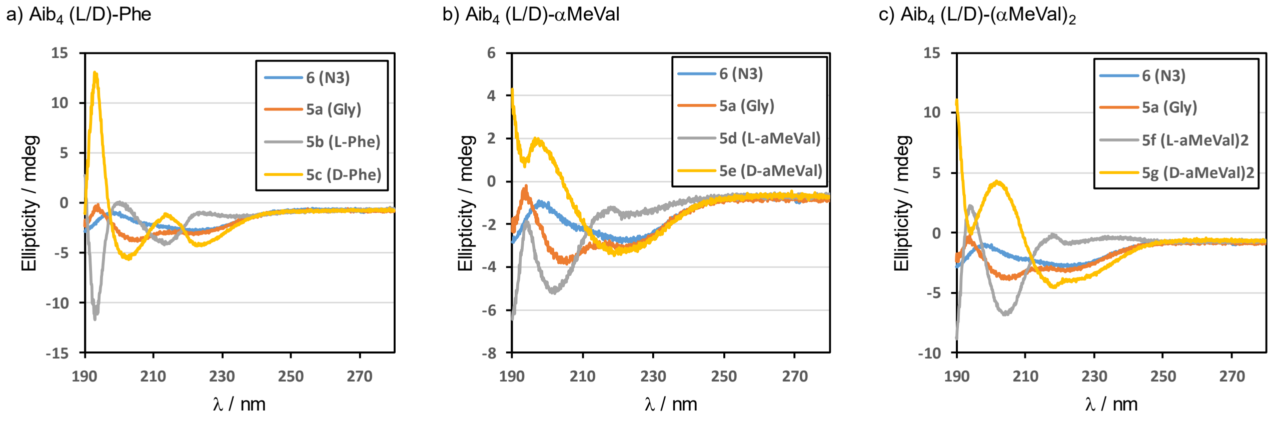

Figure S3 CD spectra of foldamers a) **5a-c**, **6** b) **5a**, **5d,e**, **6** and c) **5a**, **5f,g**, **6** in acetonitrile.

4.2 Spectra in methanol

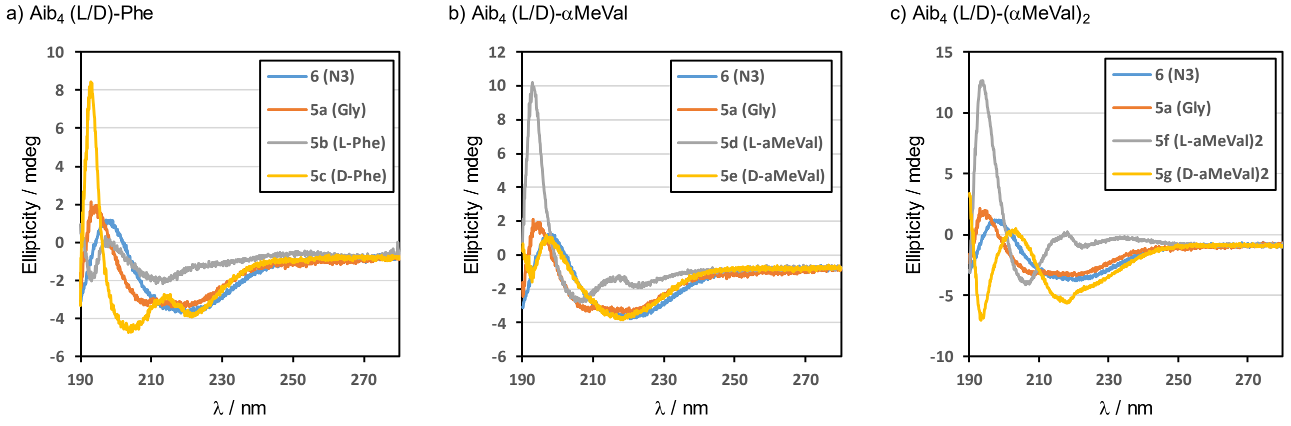

Figure S4 CD spectra of foldamers a) **5a-c**, **6** b) **5a**, **5d,e**, **6** and c) **5a**, **5f,g**, **6** in methanol.

5. Analysis of foldamers by NMR spectroscopy in organic solvents

Unless stated otherwise, NMR spectra were recorded at 298 K.

5.1 ^{1}H NMR spectroscopy in CD_3CN and CD_3OD

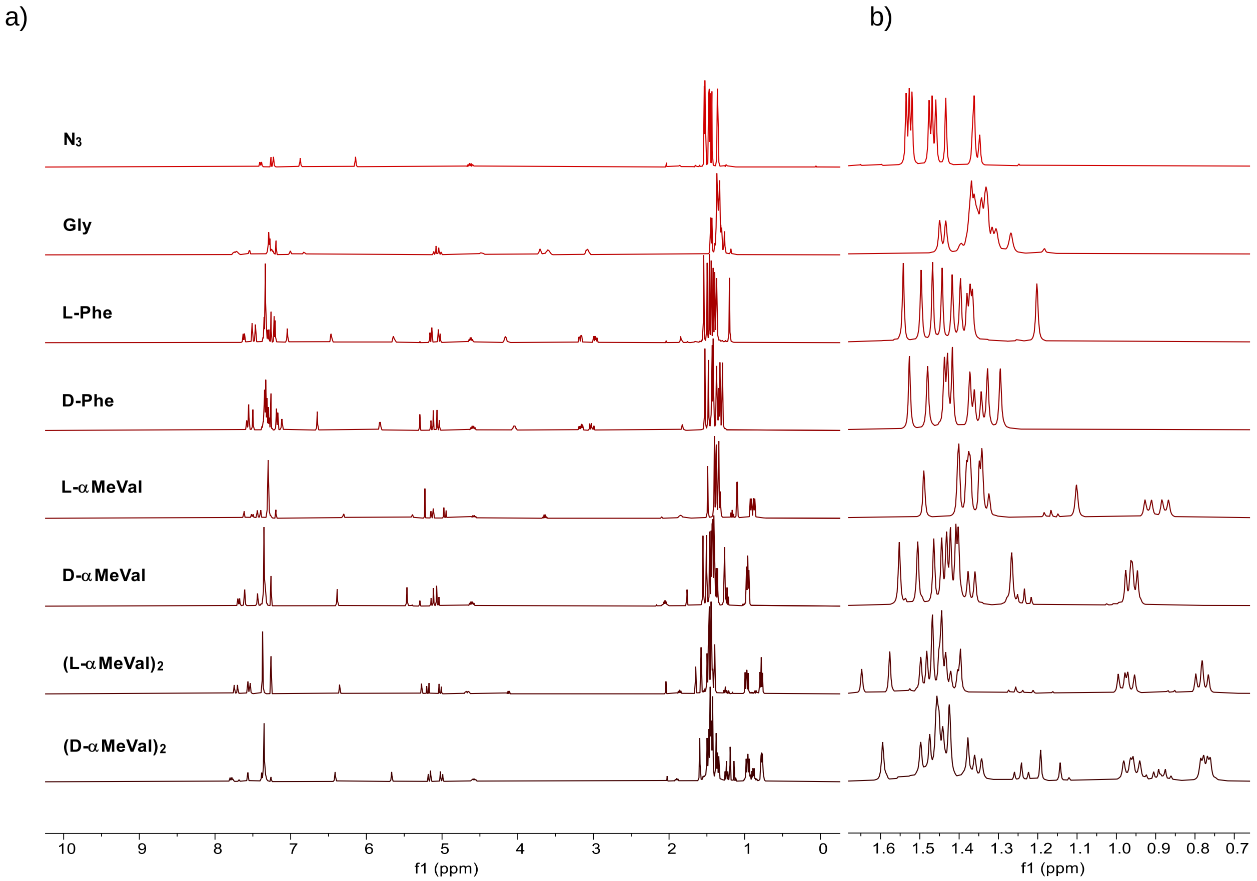

Figure S5 (a) Full sweep width and (b) expanded ^{1}H NMR spectra showing the methyl region (400 MHz, CD_3CN, 298 K). From top to bottom: N_3 (**6**), Gly (**5a**, methylene of Gly appears as a broad signal from 3.80–3.66 ppm), L-Phe (**5b**), D-Phe (**5c**), L-αMeVal (**5d**), D-αMeVal (**5e**), (L-αMeVal)$_2$ (**5f**). (D-αMeVal)$_2$ (**5g**).

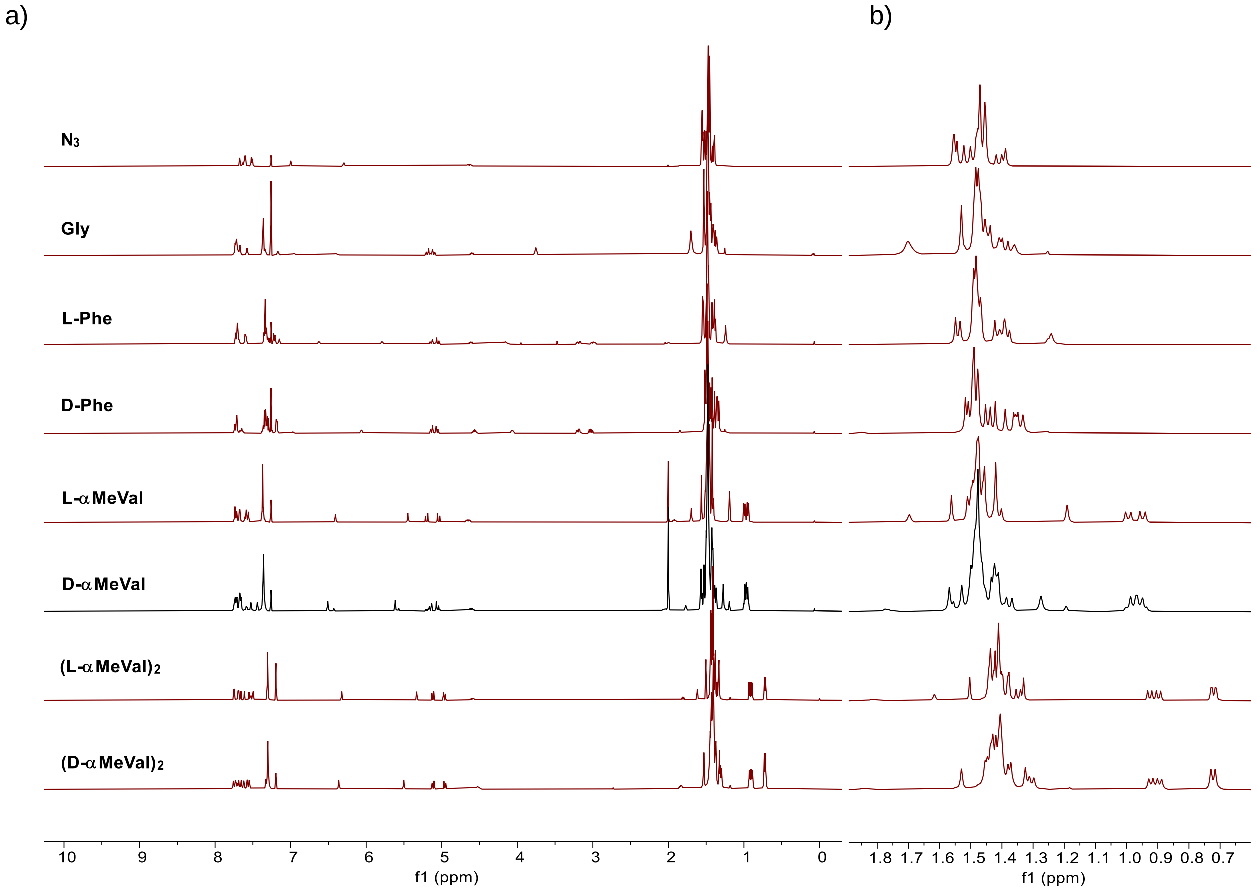

Figure S6 (a) Full sweep width and (b) expanded ^{1}H NMR spectra showing the methyl region (400 MHz, CD_3OD, 298 K). From top to bottom: N_3 (**6**), Gly (**5a**, methylene of Gly appears as a broad signal from ~3.80 ppm), L-Phe (**5b**), D-Phe (**5c**), L-αMeVal (**5d**), D-αMeVal (**5e**), (L-αMeVal)$_2$ (**5f**). (D-αMeVal)$_2$ (**5g**).

5.2 ^{19}F NMR spectroscopy in CD_3CN and CD_3OD

Each sample was dissolved in the appropriate solvent (CD_3OD or CD_3CN) with hexafluorobenzene (0.136 mM) added as an internal standard (at –165.37 ppm or –164.38 ppm respectively).

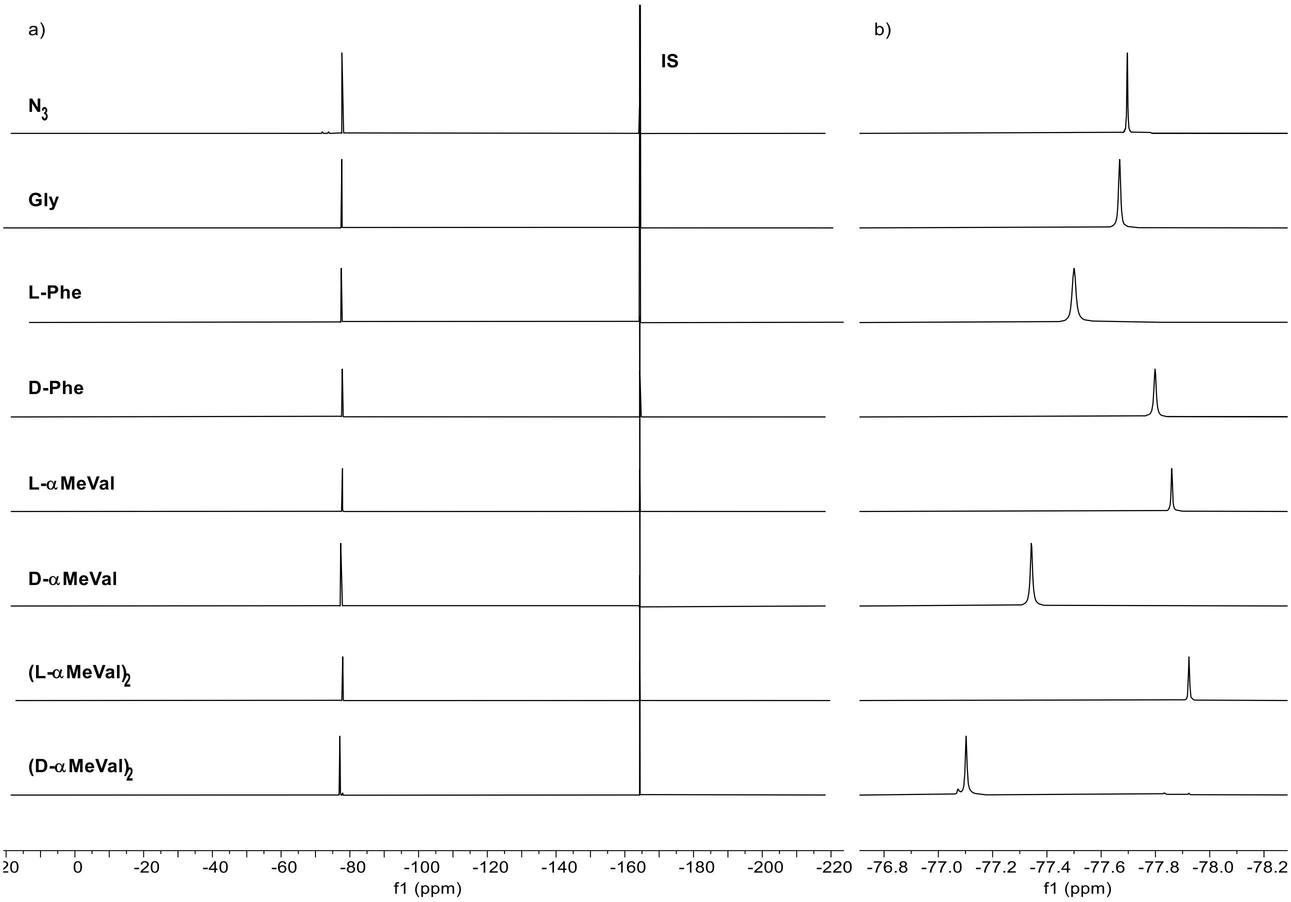

Figure S7 (a) Full sweep width and (b) expanded ^{19}F NMR spectra (all 376 MHz except **5a** 470 MHz, CD_3CN, 298 K). From top to bottom: N_3 (**6**), Gly (**5a**), L-Phe (**5b**), D-Phe (**5c**), L-αMeVal (**5d**), D-αMeVal (**5e**), L-(αMeVal)$_2$ (**5f**). D-(αMeVal)$_2$ (**5g**). IS: C_6F_6 at –164.38 ppm.[S2]

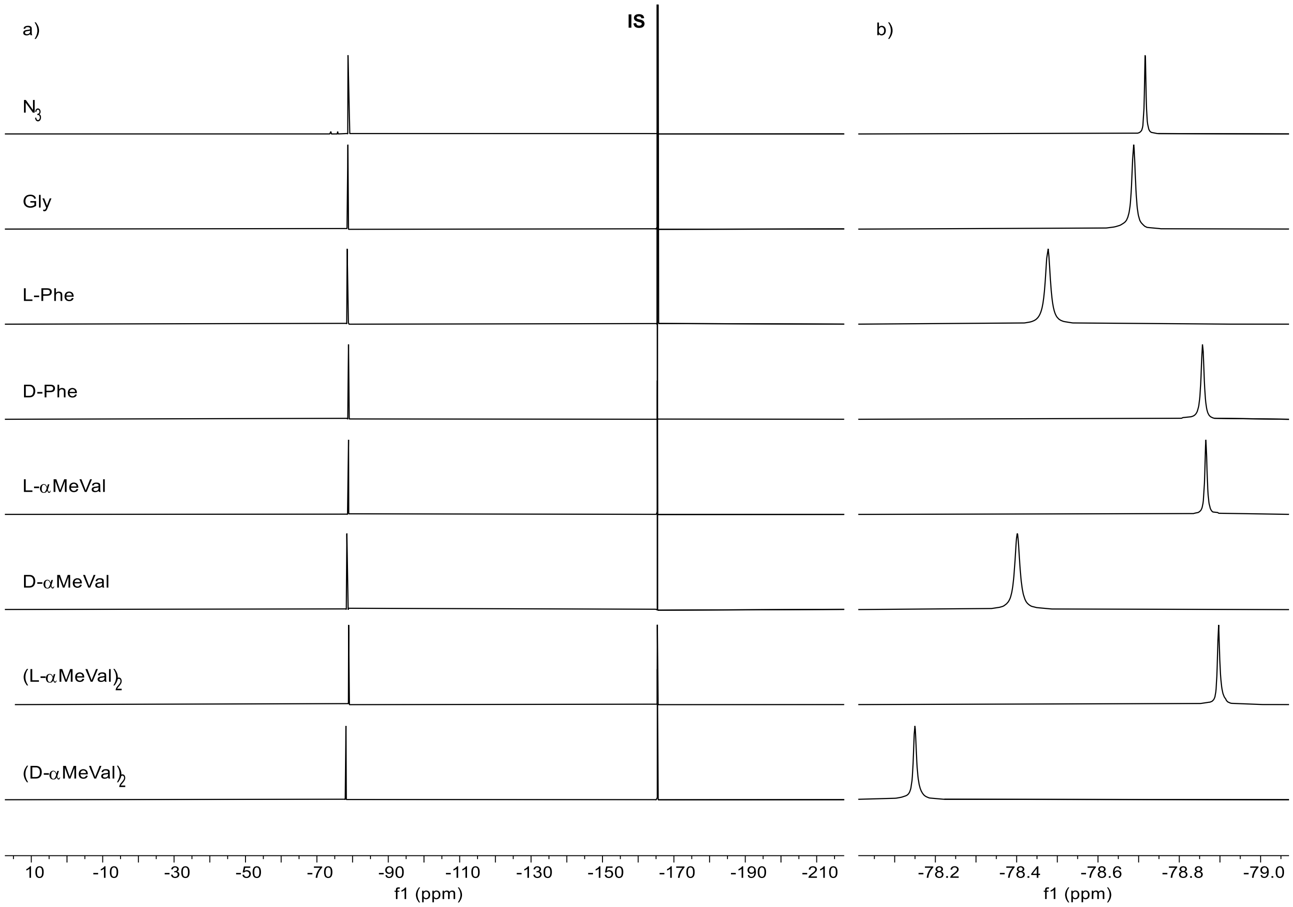

Figure S8 (a) Full sweep width and (b) expanded ^{19}F NMR spectra (all 376 MHz except **5b** 470 MHz, CD_3OD, 298 K). From top to bottom: N_3 (**6**), Gly (**5a**), L-Phe (**5b**), D-Phe (**5c**), L-αMeVal (**5d**), D-αMeVal (**5e**), (L-αMeVal)$_2$ (**5f**). (D-αMeVal)$_2$ (**5g**). IS: C_6F_6 at –165.37 ppm.[S2]

Table S1 Differences in the ^{19}F NMR chemical shift (in ppb) for Aib tetramers **6**, **5b**-**5g** compared to foldamer **5a**, both in CD_3OD and in CD_3CN.

Tetramers (Aib$_4$)	$\Delta\delta_{CD_3CN}$[a,d]	$\Delta\delta_{CD_3OD}$[b,d]	*h.e.*$_0$[c]
Gly (**5a**)	0	0	0
N_3 (**6**)	−28	−28	0
L-Phe (**5b**)	+168	+211	−52
D-Phe (**5c**)	−131	−169	+52
L-αMeVal (**5d**)	−192	−178	+68
D-αMeVal (**5e**)	+325	+286	−68
(L-αMeVal)$_2$ (**5f**)	–256	−209	+95
(D-αMeVal)$_2$ (**5g**)	+566	+538	−95

(a) from −77.696 ppm; (b) from −78.716 ppm; (c) at chiral residue[S9] (d) values in parts per billion (ppb).

5.3 VT-NMR spectra

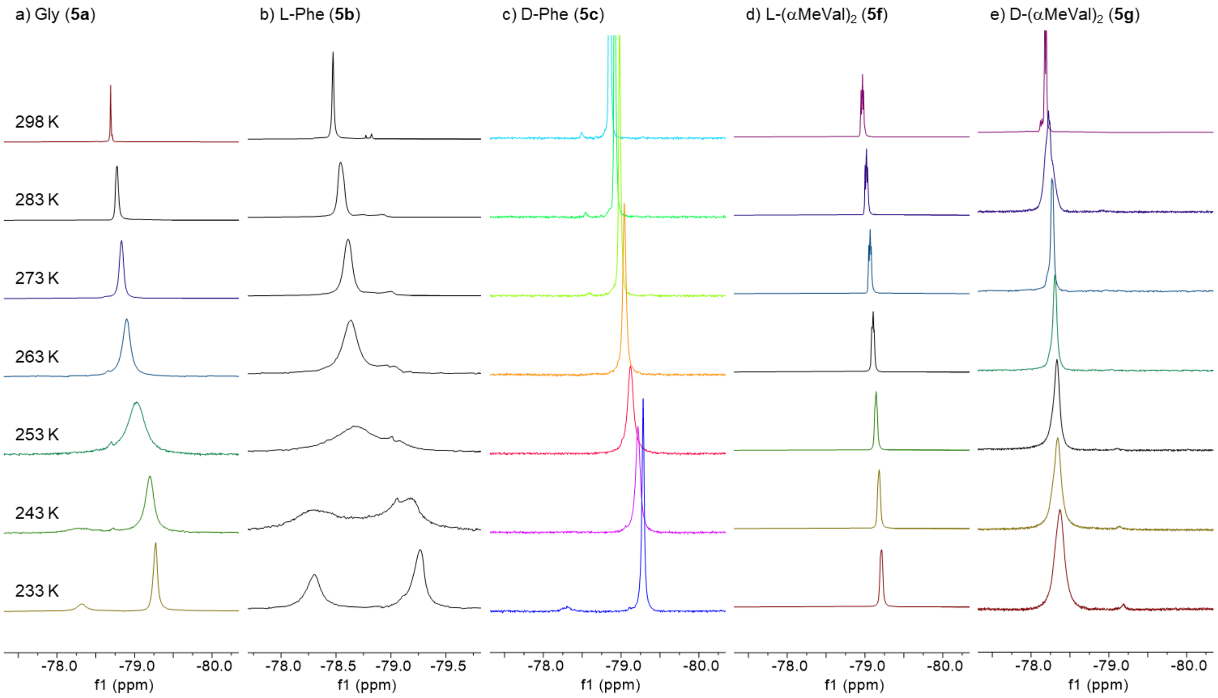

Figure S9 a)-e) Partial stacked ^{19}F VT-NMR spectra (CD_3OD, 470 MHz) of foldamers **5a-c** (all proton decoupled) and **5f-g** (both proton coupled). IS: C_6F_6 at –165.37 ppm (not shown).[S2]

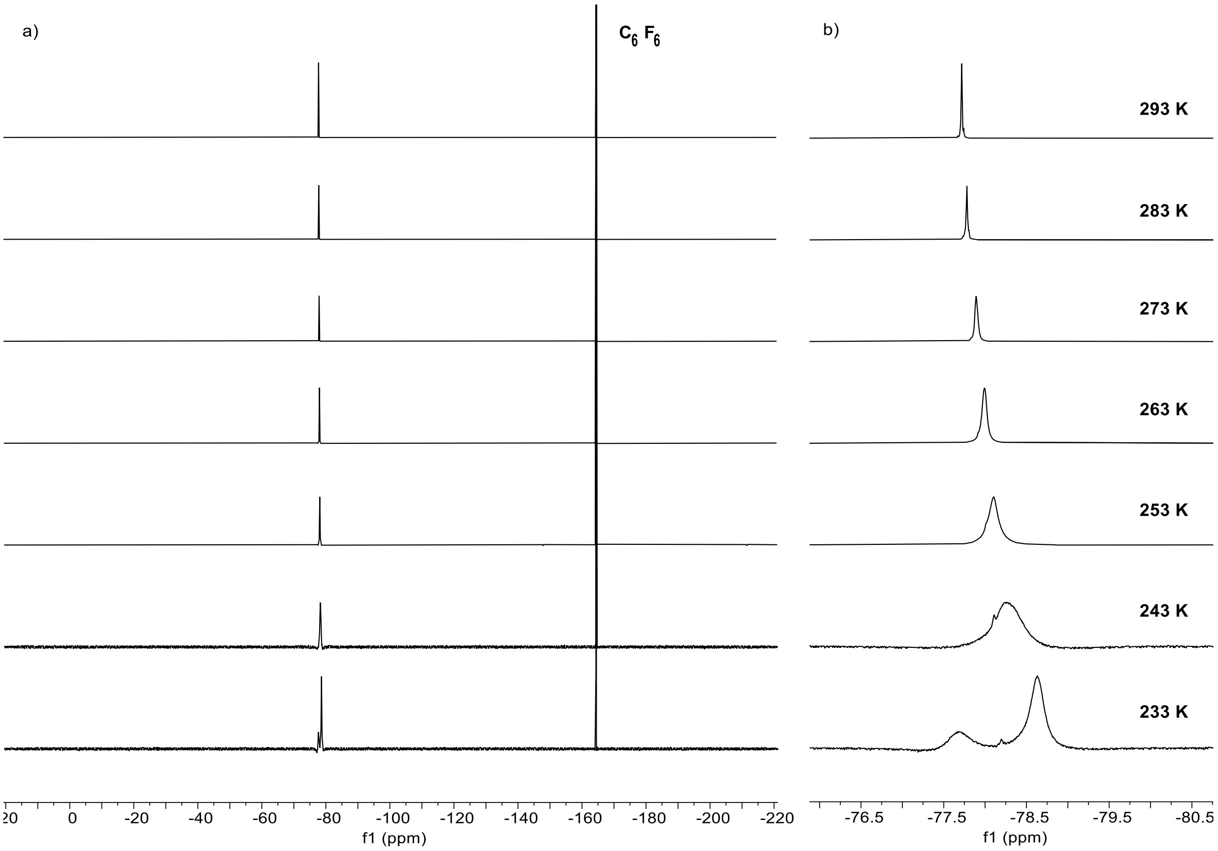

Figure S10 a) Full sweep width and b) expanded ^{19}F VT-NMR spectra (470 MHz, CD_3CN) of tetramer **5a**. IS: C_6F_6 at –164.38 ppm.[S2]

6. Modelling of ^{19}F chemical shift in organic solvents

The simple model was proposed to explain how the net helical excess (*h.e.*) depended upon the screw-sense preferences of the chiral groups at each terminus.

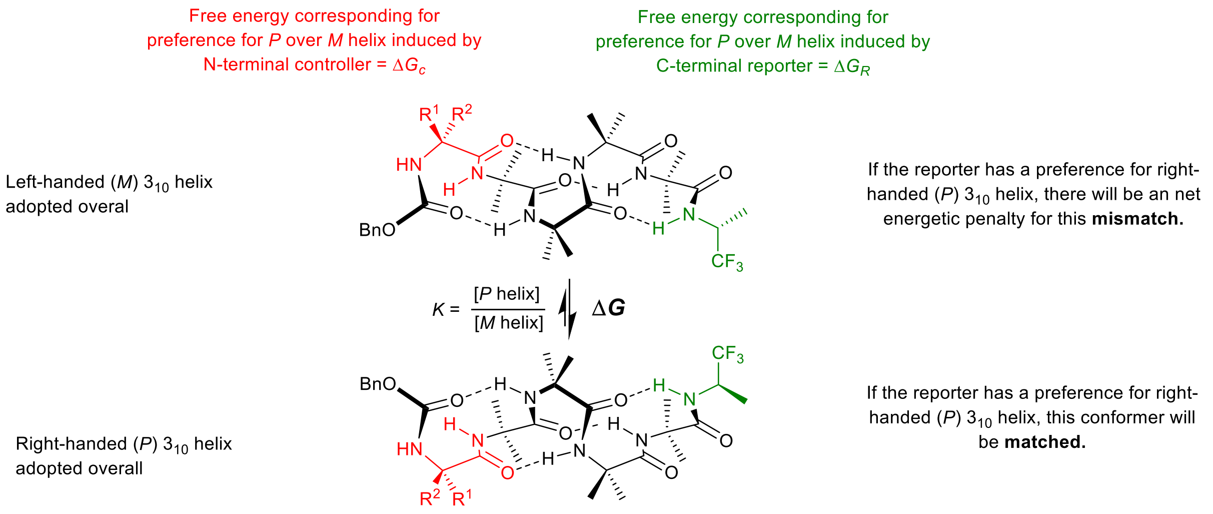

In this model, the net free energy change for adoption of a right-handed *P* helix from a left handed *M* helix is the sum of free energy terms corresponding to the screw-sense preferences of the chiral groups at each terminus, i.e. ΔG_C for the screw-sense preference of the N-terminal controller and ΔG_R for the screw-sense preference of the C-terminal reporter.

$$\Delta G = \Delta G_C + \Delta G_R$$

We could derive the term ΔG_C from published data for $h.e._0$ induced by Aib tetramers with different chiral residues at the N-terminus.[S9]

The corresponding free energy term ΔG_R from the novel C-terminal reporter group is unknown, but can be estimated from the VT-NMR data for compounds without a chiral group at the N-terminus. For example, this allows the estimation of ΔG_R from the slow exchange spectra for **5a** at 233 K in different solvents. Integration of the two signals observed at 233 K provided the ratio of conformers, the value of *K* for these compounds and thereby the corresponding free energy for induction of right-handed helix by the C-terminal reporter group.

In this way, integration of the ^{19}F NMR resonances for left-handed helix (smaller peak, found at −78.31 ppm in CD_3OD) and right-handed (larger peak, found at −79.27 ppm in CD_3OD) for **5a** gave:

K = 3.76 in CD_3OD at 233 K ($\Delta G_R = -3.28$ kJmol^{-1}; −3.4 kJmol^{-1} gave best fit to data).

K = 3.70 in CD_3CN at 233 K ($\Delta G_R = -3.24$ kJmol^{-1})

A range (from −1 to +1) of $h.e._0$ values induced directly adjacent to a chiral residue at the N-terminus of Aib tetramers have been measured. Reported values exist for L/D-Phe, L/D-αMeVal and (L/D-αMeVal)$_2$. Each of these $h.e._0$ were then converted into an equilibrium constant and thereby into a free energy change, ΔG_C.

For each chiral controller, the respective ΔG_C value was added to the appropriate ΔG_R value (in CD_3OD at 233 K, ΔG_R estimated as −3.40 kJmol^{-1}; in CD_3CN at 233 K, ΔG_R estimated as −3.24 kJmol^{-1}) to give a series of net free energy changes (ΔG) for the *P* helix/*M* helix equilibrium in each solvent.

Once calculated, each value (ΔG) was converted back into K, allowing the proportion of M helix and P helix to be calculated for each different N-terminal group.

Finally to replicate the position of the trifluoromethyl resonance in the ^{19}F NMR spectrum ($\delta(CF_3)$) of each foldamer, which is the fast exchange averaged resonance of M and P helices, the weighted average signal was calculated using estimates of the chemical shifts for both M helix and P helix in each solvent.

Exemplar spreadsheets and plots are shown for foldamers **5a-g** and **6** in both CD_3OD and CD_3CN. The solid lines are the calculated fits, whereas the points correlate the reported *h.e*. values in Aib tetramers for azido, Gly, L-Phe, L-αMeVal, (L-αMeVal)$_2$, D-Phe, D-αMeVal and (D-αMeVal)$_2$ controllers[S9] with the measured δ_{CF3} values for the corresponding foldamers **5a-5g**, **6** studied in this work.

Furthermore, the sequence of calculations described above can be summarised by two equations (1) and (2). *The K* value (the *P*/*M* ratio) for each foldamer (Equation 1) can be calculated by:

$$K = \left(\frac{1 + h.e._0}{1 - h.e._0}\right) e^{\frac{(-\Delta G_{\mathrm{R}})}{RT}} \qquad (1)$$

The weighted average ^{19}F signal at 298 K for each foldamer is then calculated (Equation 2, using estimated δ_M (for M helix) = −78.03 ppm and δ_P (for P helix) = −78.90 ppm at 298 K).

$$\delta = \frac{K\delta_P + \delta_M}{K + 1} \qquad (2)$$

Table S2 Modelled δ(CF_3) values for foldamers **5a**-**g** and **6** in CD_3CN, calculated using Microsoft® Excel.

Hypothetical *h.e.*₀ at N terminus	*K* from *h.e.*₀	Δ*G*C / kJ/mol	ΔGC with ΔGR / kJ/mol	*K* from Δ*G*C + Δ*G*R	average δ(CF3) / ppm	actual δ(CF3) in CD3CN / ppm
-0.99	0.005025126	13.11454382	9.894543821	0.0184324	-77.04592694	
-0.95	0.025641026	9.076737755	5.856737755	0.0940525	-77.10565103	-77.153
-0.9	0.052631579	7.29505957	4.07505957	0.193055132	-77.17239788	
-0.8	0.111111111	5.443782091	2.223782091	0.407560834	-77.284805	
-0.75	0.142857143	4.8211325	1.6011325	0.524006786	-77.33257475	
-0.7	0.176470588	4.297599006	1.077599006	0.647302501	-77.37579332	
-0.68	0.19047619	4.108379452	0.888379452	0.698675715	-77.39194938	-77.343
-0.6	0.25	3.434644093	0.214644093	0.917011876	-77.45095224	
-0.52	0.315789474	2.855846479	-0.364153521	1.158330791	-77.50227751	-77.532
-0.5	0.333333333	2.721891045	-0.498108955	1.222682501	-77.514082	
-0.4	0.428571429	2.099241455	-1.120758545	1.572020359	-77.56785652	
-0.3	0.538461538	1.53371421	-1.68628579	1.975102502	-77.61421187	
-0.2	0.666666667	1.004568999	-2.215431001	2.445365003	-77.65458439	
-0.1	0.818181818	0.497176096	-2.722823904	3.001129776	-77.69006212	
0	1	0	-3.22	3.668047504	-77.72148435	-77.717
0	1	0	-3.22	3.668047504	-77.72148435	-77.696
0.1	1.222222222	-0.4971761	-3.717176096	4.483169172	-77.74950887	
0.2	1.5	-1.004569	-4.224568999	5.502071257	-77.77465851	
0.3	1.857142857	-1.53371421	-4.75371421	6.812088223	-77.79735406	
0.4	2.333333333	-2.09924145	-5.319241455	8.55877751	-77.81793802	
0.5	3	-2.72189105	-5.941891045	11.00414251	-77.83669197	
0.52	3.166666667	-2.85584648	-6.075846479	11.61548376	-77.84024445	-77.816
0.6	4	-3.43464409	-6.654644093	14.67219002	-77.85384958	
0.68	5.25	-4.10837945	-7.328379452	19.2572494	-77.86655876	-77.860
0.7	5.666666667	-4.29759901	-7.517599006	20.78560253	-77.86960635	
0.75	7	-4.8211325	-8.0411325	25.67633253	-77.87701196	
0.8	9	-5.44378209	-8.663782091	33.01242754	-77.8841271	
0.9	19	-7.29505957	-10.51505957	69.69290258	-77.89755179	
0.95	39	-9.07673775	-12.29673775	143.0538527	-77.90389117	-77.924
0.99	199	-13.1145438	-16.33454382	729.9414534	-77.90879607	

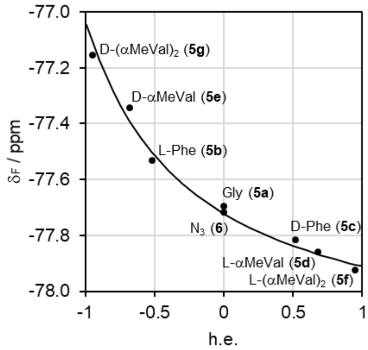

Figure S11 ^{19}F NMR chemical shifts in CD_3CN (ppm) for Aib tetramers **5a**-**5g** and **6** correlated with reported ability of each chiral N-terminal group to induce a local helical excess. Curve fits assume $\Delta G_R = -3.22$ kJ mol^{-1}.

Table S3 Modelled δ(CF_3) values for foldamers **5a**-**g** and **6** in CD_3OD, calculated using MIcrosfot® Excel.

Hypothetical *h.e.*₀ at N terminus	*K* from *h.e.*₀	ΔG_C / kJ/mol	ΔG_C with ΔG_R / kJ/mol	*K* from ΔG_C + ΔG_R	average δ(CF_3) / ppm	actual δ(CF_3) in CD_3OD / ppm
-0.99	0.005025126	13.11454382	9.714543821	0.019821391	-78.04690944	
-0.95	0.025641026	9.076737755	5.676737755	0.101139921	-78.10990967	-78.15
-0.9	0.052631579	7.29505957	3.89505957	0.207602995	-78.17956456	
-0.8	0.111111111	5.443782091	2.043782091	0.43827299	-78.29510788	
-0.75	0.142857143	4.8211325	1.4211325	0.563493844	-78.34355393	
-0.7	0.176470588	4.297599006	0.897599006	0.696080631	-78.38705269	
-0.68	0.19047619	4.108379452	0.708379452	0.751325125	-78.4032333	-78.402
-0.6	0.25	3.434644093	0.034644093	0.986114227	-78.46195873	
-0.52	0.315789474	2.855846479	-0.544153521	1.245617971	-78.5125788	-78.477
-0.5	0.333333333	2.721891045	-0.678108955	1.314818969	-78.52416067	
-0.4	0.428571429	2.099241455	-1.300758545	1.690481532	-78.57663781	
-0.3	0.538461538	1.53371421	-1.86628579	2.123938335	-78.62150539	
-0.2	0.666666667	1.004568999	-2.395431001	2.629637939	-78.66030667	
-0.1	0.818181818	0.497176096	-2.902823904	3.227282925	-78.69419404	
0	1	0	-3.4	3.944456908	-78.72404539	-78.688
0	1	0	-3.4	3.944456908	-78.72404539	-78.716
0.1	1.222222222	-0.4971761	-3.897176096	4.821002888	-78.75054122	
0.2	1.5	-1.004569	-4.404568999	5.916685362	-78.77421721	
0.3	1.857142857	-1.53371421	-4.93371421	7.325419972	-78.79550077	
0.4	2.333333333	-2.09924145	-5.499241455	9.203732786	-78.81473709	
0.5	3	-2.72189105	-6.121891045	11.83337072	-78.83220799	
0.52	3.166666667	-2.85584648	-6.255846479	12.49078021	-78.83551151	-78.857
0.6	4	-3.43464409	-6.834644093	15.77782763	-78.84814585	
0.68	5.25	-4.10837945	-7.508379452	20.70839877	-78.85992335	-78.866
0.7	5.666666667	-4.29759901	-7.697599006	22.35192248	-78.86274397	
0.75	7	-4.8211325	-8.2211325	27.61119836	-78.86959233	
0.8	9	-5.44378209	-8.843782091	35.50011217	-78.87616446	
0.9	19	-7.29505957	-10.69505957	74.94468125	-78.88854429	
0.95	39	-9.07673775	-12.47673775	153.8338194	-78.89438107	-78.897
0.99	199	-13.1145438	-16.51454382	784.9469247	-78.89889306	

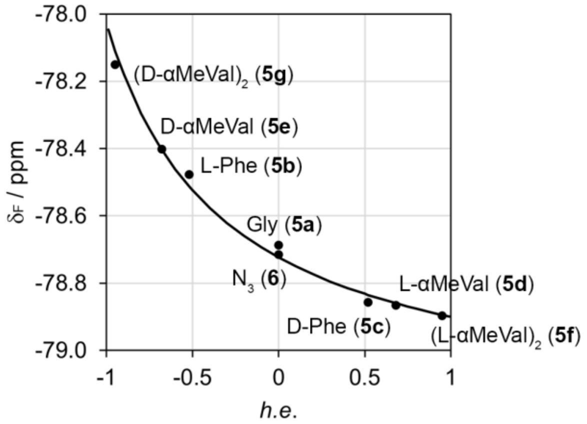

Figure S12 ^{19}F NMR chemical shifts in CD_3OD (ppm) for Aib tetramers **5a**-**5g** and **6** correlated with reported ability of each chiral N-terminal group to induce a local helical excess. Curve fits assume ΔG_R = −3.40 kJ mol^{-1}.

7. Analysis of foldamers in membranes

7.1 Preparation of small unilamellar vesicles (SUVs) by sonication

The procedure used was a slight modification of reported protocols.[S10,11] The appropriate foldamer (0.003 mmol) was dissolved in $CHCl_3$ (0.5 mL) and sonicated in a bath water for 15 min at room temperature. The solution was then transferred to a rounded-bottomed flask containing 1,2-dioleoyl-*sn*-glycero-3-phosphocholine (DOPC, 20 mg, 0.025 mmol) and sonicated for further 15 min at room temperature. The solvent was then removed using a rotary evaporator. The lipid film was dried under reduced pressure for at least 6 h. The film was then re-suspended in buffer (0.5 mL, NaCl 100 mM, MOPS 20 mM, pH 7.4, with KF 0.25 mg/mL, 10% D_2O) and sonicated in a bath sonicator for 3.5~4 h at approx. 25 °C. The resulting lipid suspension (11 mol % foldamer, 50 mM DOPC lipid) was then transferred into a NMR tube for analysis.

7.2 Preparation of SUVs containing 5b and 5c (1:1 ratio)

Compounds **5b** and **5c** (2.0 mg each, 0.0027 mmol each) were dissolved in $CHCl_3$ (0.5 mL) and sonicated for 15 min at room temperature. The solution was then transferred to a flask containing DOPC (20 mg, 0.025 mmol) and sonicated for further 15 min at room temperature. The solvent was then removed using a rotary evaporator. The lipid film was dried under reduced pressure for at least 6 h. The lipid film was then re-suspended in buffer (0.5 mL, NaCl 100 mM, MOPS 20 mM, pH 7.4, with KF 0.25 mg/mL, 10% D_2O) and sonicated in the bath sonicator for 3.5–4 h at room temperature. The resulting suspension was then transferred into a NMR tube for analysis.

7.3 Dynamic light scattering (DLS) analysis

Samples for DLS analysis were prepared as described in Section 7.1 (bath sonication for ~4 h, buffer was pre-filtered through a 0.2 mm pore size Acrodisk before addition to the vesicle film). Averaged hydrodynamic diameter values are calculated from 5 independent measurements from different batches.

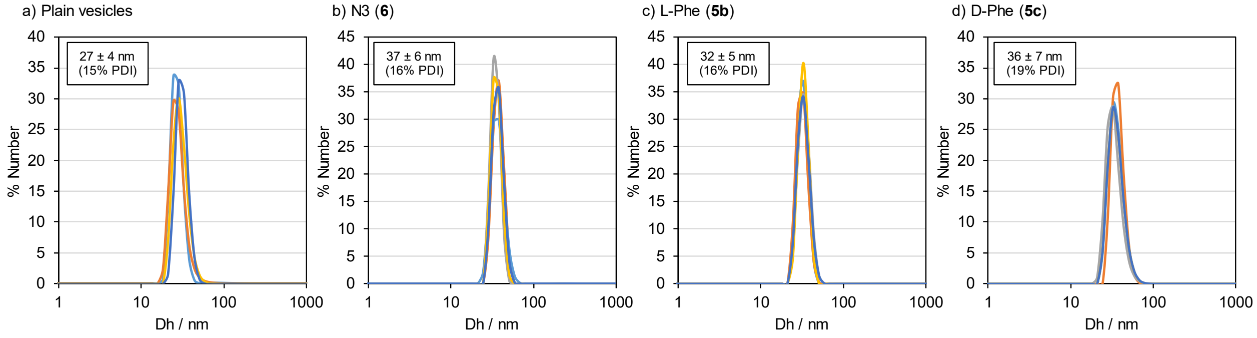

Figure S13 Dynamic light scattering (DLS) analysis from five independent measurements of plain DOPC SUVs (a) and SUVs prepared with foldamers b) **6**, c) **5b** and d) **5c**.

7.4 ^{19}F NMR experiment for analysis of foldamers in membranes

Parameters:

Solvent: H_2O+D_2O; Temperature: 298.0 K; Pulse Sequence: zgig; Experiment: 1D; Probe: Z130709_0001 (CP QCI 500S2 H/F-C/N-D-05 Z LT); Number of Scans: 512; Receiver Gain: 2050.0; Relaxation Delay: 1.0000; Pulse Width: 12.0000; Acquisition Time: 0.5767; Spectrometer Frequency: 470.49; Spectral Width: 113636.4;

Lowest Frequency: -103871.8; Nucleus: 19F; Acquired Size: 65536; Spectral Size: 131072; Digital Resolution: 0.87; Total experiment time: 22 min.

Data processing:

The data was processed with MestReNova. For phase correction, usually, 'automatic phase correction' was used. 'Manual correction' was used when automatic correction could not give a good spectrum. For baseline correction, the 'Whittaker Smoother' method was used. For ^{19}F NMR spectra of SUVs, the apodization, exponential is used and set to 10 Hz (0.3 Hz for ^{1}H solution NMR)

7.5 ^{19}F DOSY NMR

DOSY NMR can be used to analyse the diffusion coefficients of vesicles, with the diffusion coefficients of vesicles typically two orders of magnitude smaller than those of small molecules in solution.[S12,13] As shown by ^{19}F DOSY NMR, SUV samples prepared with foldamer **5a** show a broad and a sharp peak at, respectively, −83 and –81 ppm. The diffusion coefficient calculated for the sharp peak (unincorporated foldamer **5a**) is ~10-fold higher than that calculated for the broad peak (incorporated foldamer). The calculated diffusion coefficients are in accordance with those reported in the literature.[S12]

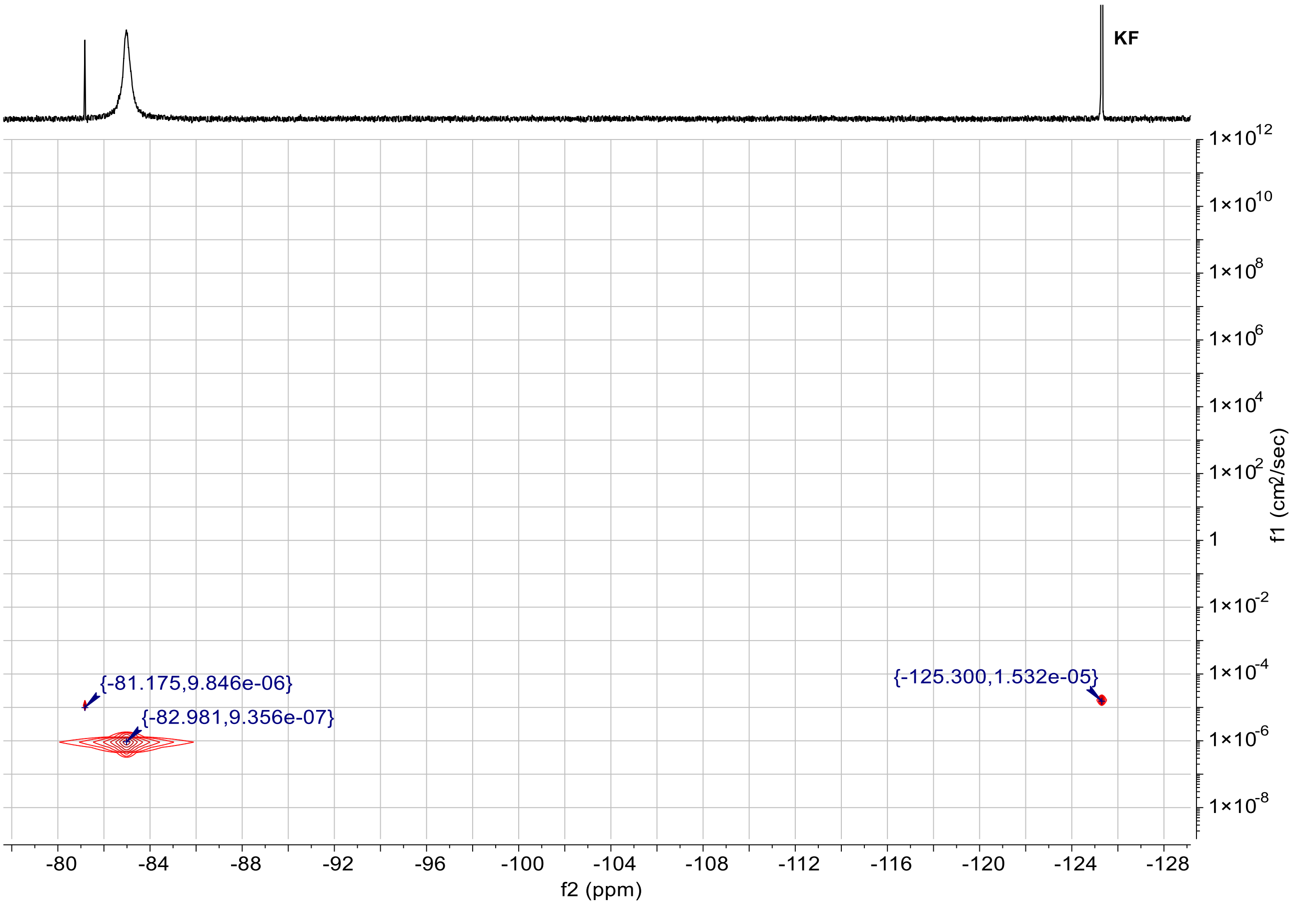

Figure S14 ^{19}F DOSY NMR data (1:9 D_2O/H_2O, 470 MHz, 298 K, [NaCl] = 100 mM, [MOPS] = 20 mM, pH 7.4) of foldamer **5a** incorporated in DOPC vesicles.

7.6 Purification by size exclusion chromatography (SEC)

SUVs prepared with foldamer **5b** were separated by size exclusion chromatography (SEC, gel permeation chromatography (GPC)) to demonstrate that the sharp peak observed in the ^{19}F NMR spectrum could be assigned to unincorporated foldamer.

General procedure: Buffer solution (25 mL, NaCl 100 mM, MOPS 20 mM, pH 7.4, with KF 0.25 mg/mL) was used to equilibrate a PD-10 SEC column (Sephadex G-25 resin) before the addition of the sample. The sample was added to the SEC column directly from the NMR tube (0.5 mL). After all the suspension was loaded into the column, another portion of buffer solution (2 mL) was added and loaded into the column. Then further buffer solution (4 mL) was added to the column. Aliquots of eluent were collected separately (1 mL in each collection). All four fractions were analyzed by ^{19}F NMR spectroscopy.

(a) (b)

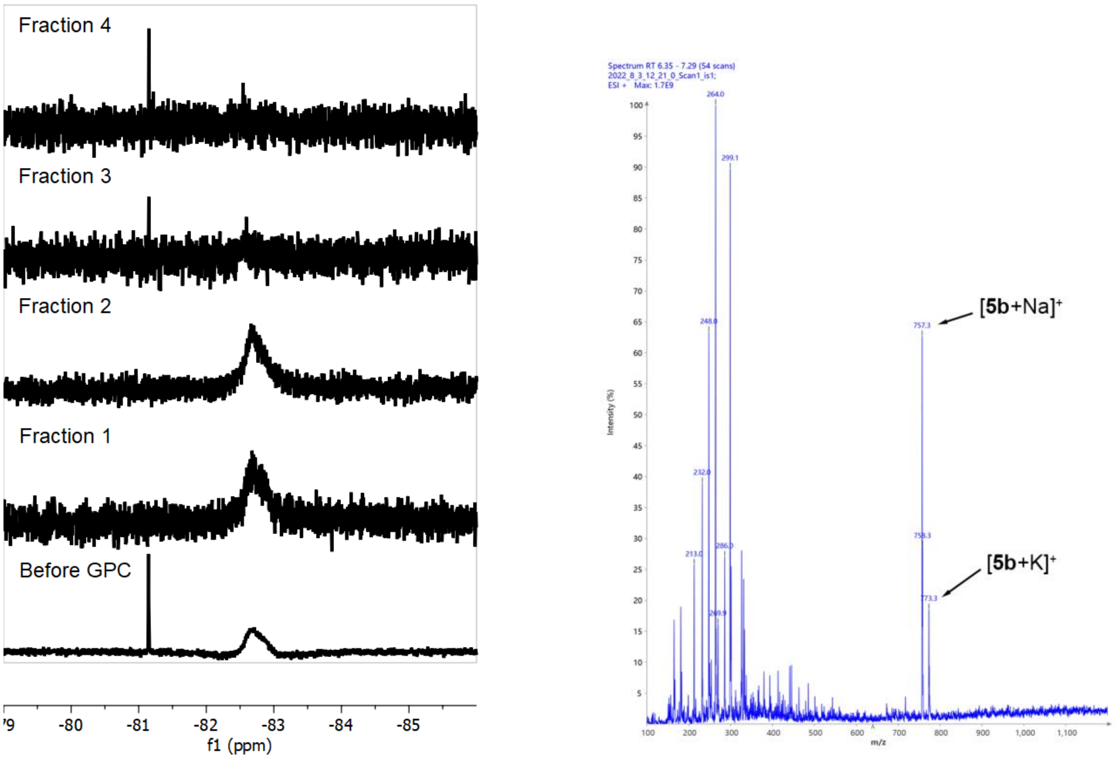

Figure S15 (a) Representative partial ^{19}F NMR spectra (1:9 D_2O/H_2O, 470 MHz, 298 K, [NaCl] = 100 mM, [MOPS] = 20 mM, pH 7.4) of **5b** incorporated in DOPC vesicles before and after purification (fractions 1 and 2) by size exclusion chromatography. IS: KF at –125.3 pm (not shown). (b) Positive electrospray MS spectrum of the SEC fraction containing the sharp peak (from SUVs containing **5b**).

7.7 Titration with $PrCl_3$

A stock solution of praseodymium(III) chloride (30 mM) was prepared in buffer (NaCl 100 mM, MOPS 20 mM, pH 7.4, 10% D_2O). Suspensions of SUVs loaded with foldamer **5b** and added fluoride (internal F^-, Figure S16) were prepared as described in Section 7.1. The sample was then transferred into an NMR tube with a coaxial-insert tube containing reference solution (2.5 M KF in 10 mL D_2O, external F^-, Figure S16).

A suspension of SUVs loaded with **5b** was titrated with $PrCl_3$ (0–5.0 mM). The NMR tube was shaken after each addition.

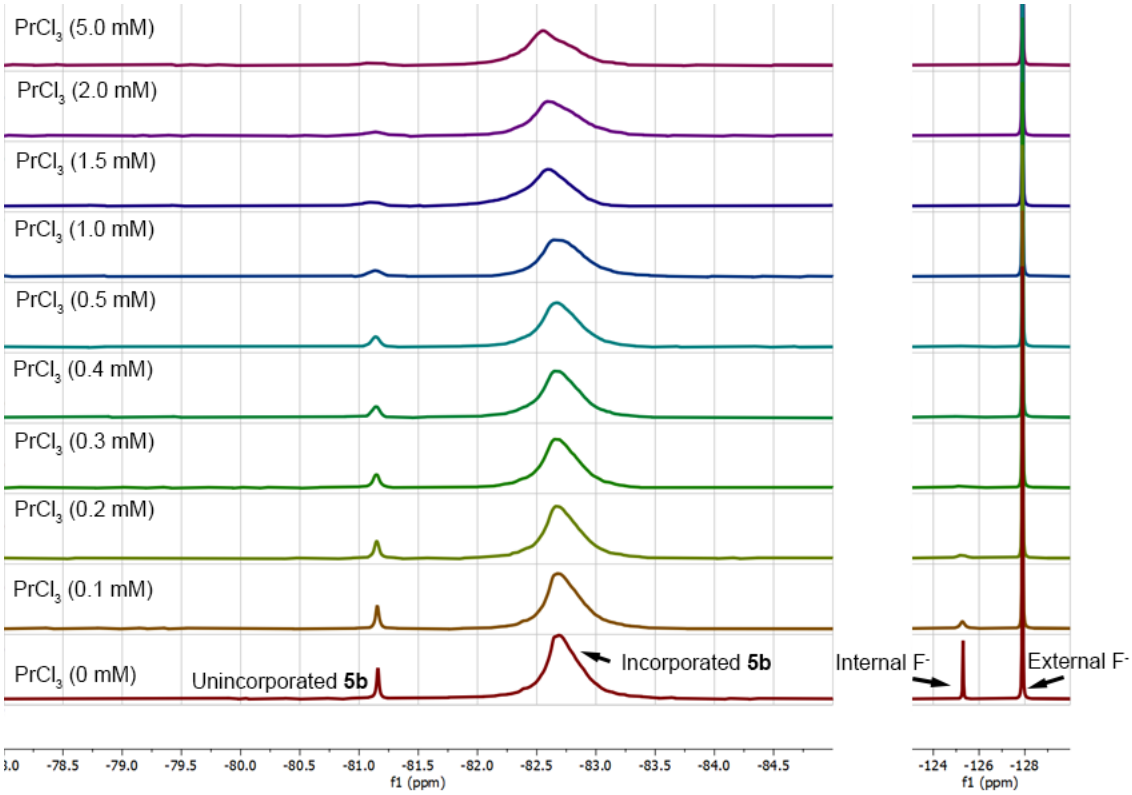

Figure S16 Partial ^{19}F NMR spectra (1:9 D_2O/H_2O, 470 MHz, 298 K, [NaCl] = 100 mM, [MOPS] = 20 mM, pH 7.4) of foldamer **5b** in DOPC vesicles titrated with $PrCl_3$ (0–5.0 mM). Spectra referenced to internal KF at −125.300 ppm and external KF at -127.9 ppm.

Although no splitting was observed in the ^{19}F NMR spectrum of the SUV-embedded foldamer **5b** after addition of $PrCl_3$, splitting was observed in the phospholipid ^{31}P NMR resonance of DOPC SUVs with $PrCl_3$ (2.0 mM), as described in the literature.[S11] When $PrCl_3$ is added to DOPC SUVs, the paramagnetic species can shift resonances arising from lipids in the outer leaflet of the bilayer, resulting in the appearance of a downfield broad peak.

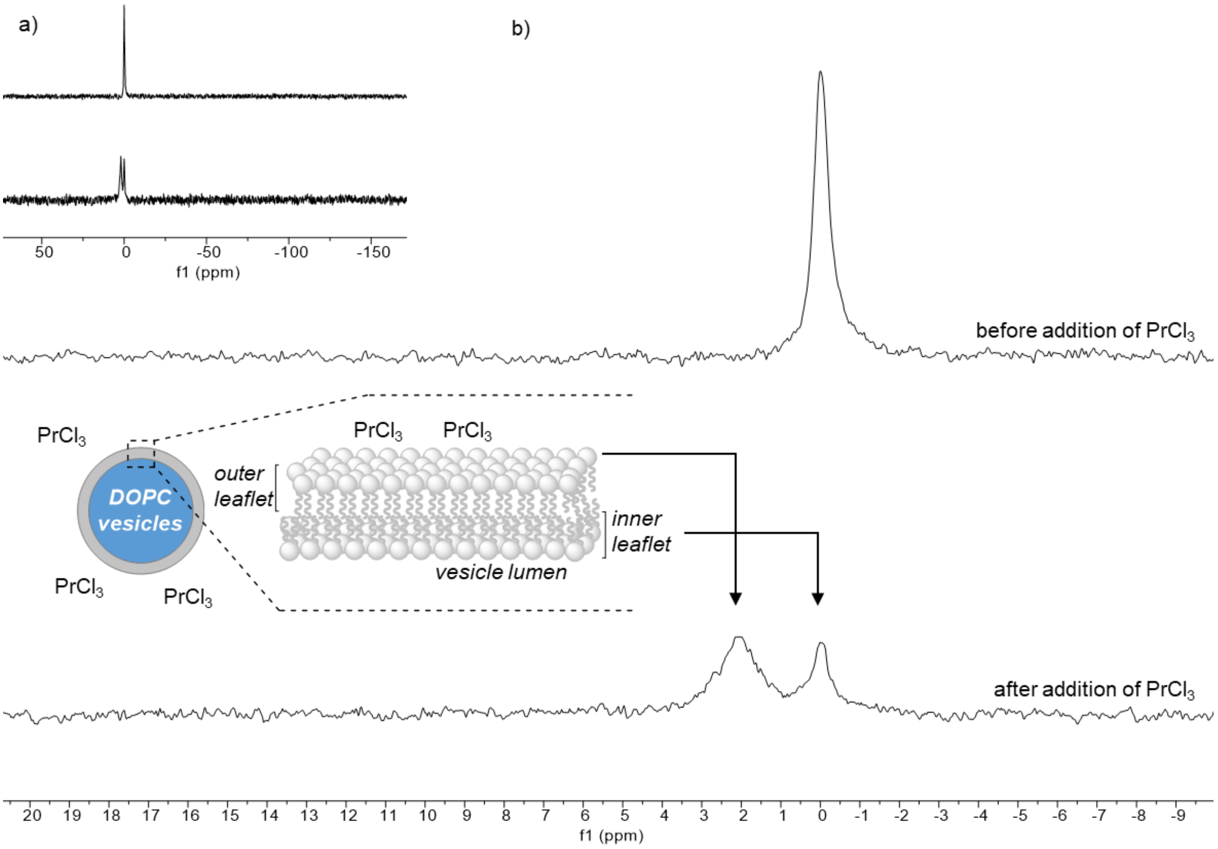

Figure S17 (a) Full sweep width and (b) partial $^{31}P\{^1H\}$ NMR spectra (1:9 D_2O/H_2O, 162 MHz, 298 K, [NaCl] = 100 mM, [MOPS] = 20 mM, pH 7.4) of plain DOPC vesicles before and after addition of $PrCl_3$ (2 mM).

7.8 Line fitting analysis of 1:1 mixture of 5b and 5c incorporated in DOPC SUVs

Line fitting analysis was performed with MNova software. Fitting options. Width Constraint: Lower: 0.10 Hz, Upper: 100.00 Hz; Position Constraint: Within ± 5.00%; Share Type: Generalized Lorentzian; Default Locks: None; Maximum Number of Fine Iterations: 100; Local Minima Filter: 5.

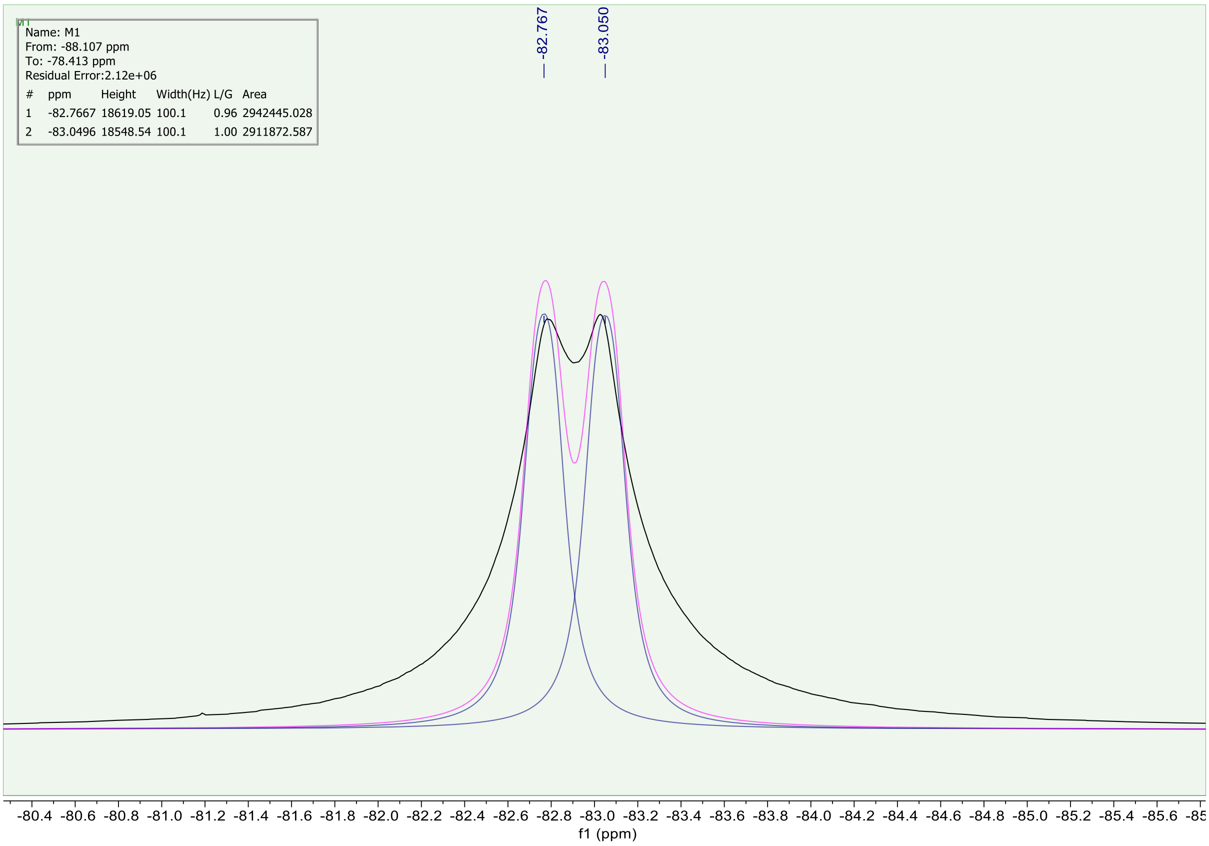

Figure S18 Partial ^{19}F NMR spectrum (1:9 D_2O/H_2O, 470 MHz, 298 K, [NaCl] = 100 mM, [MOPS] = 20 mM, pH 7.4) of 1:1 mixture of **5b** and **5c** inserted in DOPC vesicles. Spectrum referenced with KF at –125.3 ppm (not shown).

7.9 ¹⁹F NMR spectra of foldamers 6 and 7 incorporated in DOPC SUVs

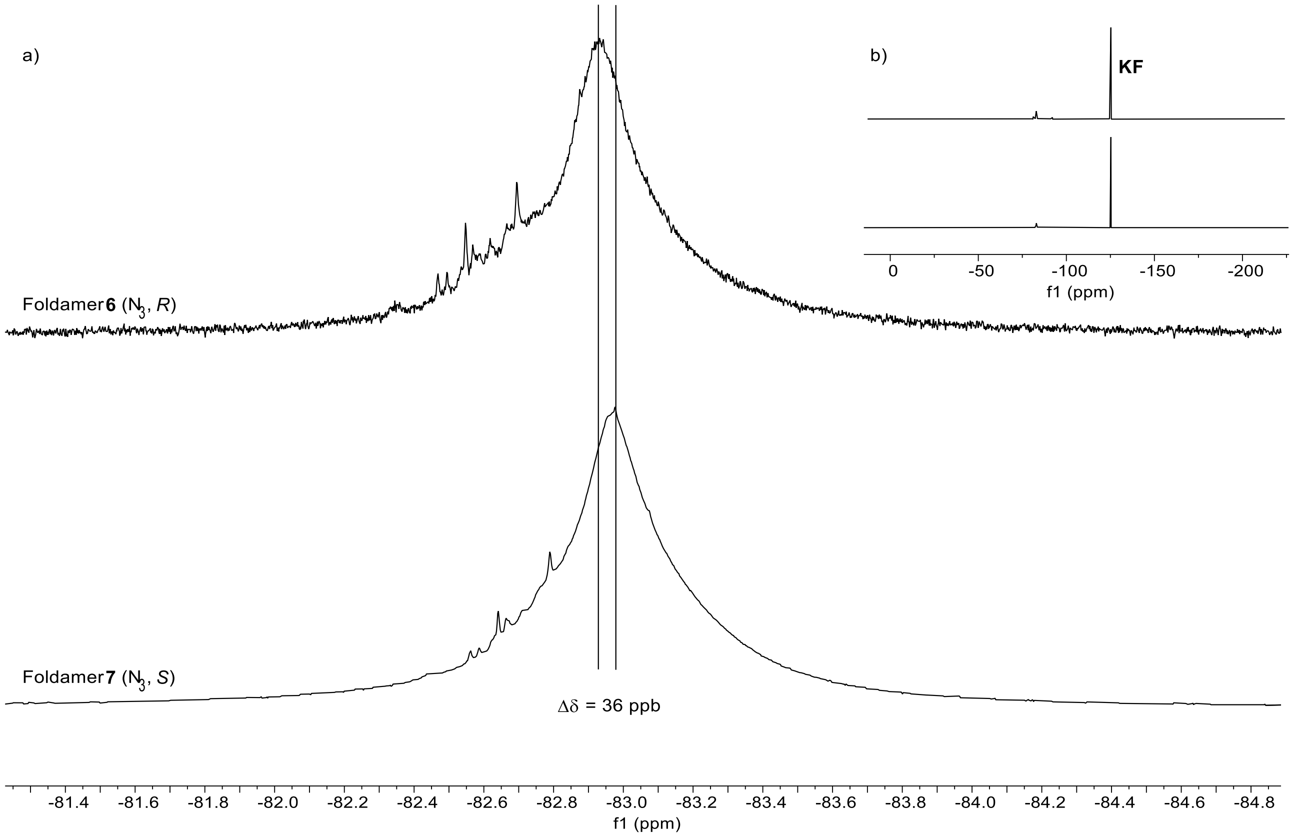

Figure S19 (a) Partial and (b) full sweep width ^{19}F NMR spectra (1:9 D_2O/H_2O, 470 MHz, 298 K, [NaCl] = 100 mM, [MOPS] = 20 mM, pH 7.4) of foldamers **6** and **7** in DOPC vesicles. Spectra referenced to KF at –125.300 ppm (shown in b)).

8. Changing foldamer loading in SUVs

SUV samples with different loadings of foldamer (2 mol%, 5 mol% and 11 mol% foldamer loading) were prepared as described in Section 7.1. Foldamer **5c** (D-Phe) was incorporated into DOPC vesicles (0.025 mmol) in increasing amounts (0.0006, 0.0014, and 0.003 mmol, respectively). The corresponding ^{19}F NMR spectra with different ratios (2 mol%, 6 mol% and 12 mol% foldamer loading, from top to bottom) do not show significant differences in ^{19}F NMR chemical shift (2 mol% loading −82.899 ppm; 5 mol% loading: −82.941 ppm; 11 mol% loading: −82.929 ppm).

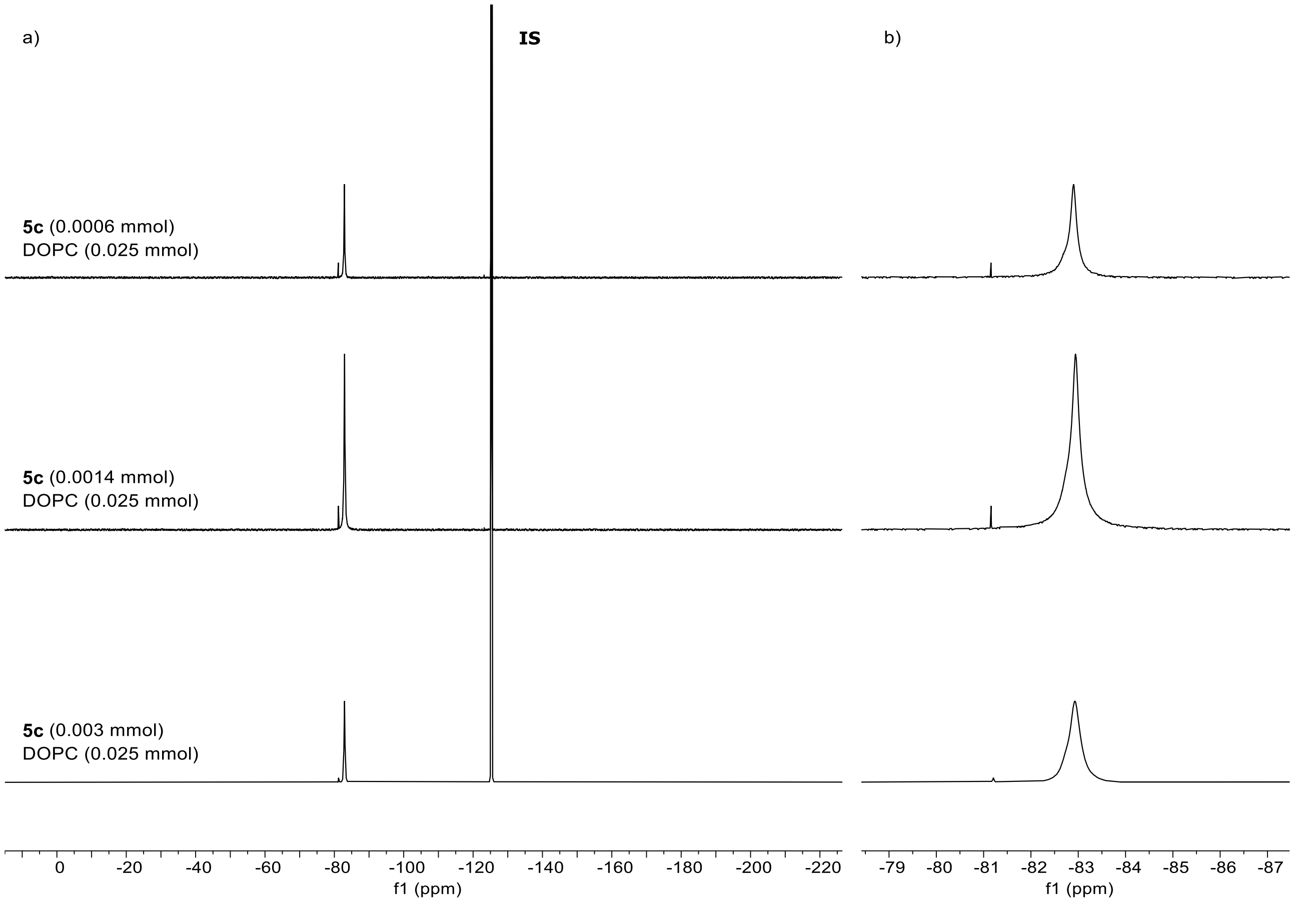

Figure S20 (a) Full sweep width and (b) partial ^{19}F NMR spectra (1:9 D_2O/H_2O, 470 MHz, 298 K, [NaCl] = 100 mM, [MOPS] = 20 mM, pH 7.4) of foldamer **5c** in DOPC vesicles (molar ratio, from top to bottom, 2 mol%, 5 mol% and 11 mol%). Spectra referenced to KF at –125.300 ppm.

9. Quantification of foldamer solubility and self-association in $CHCl_3$

9.1 Experimental procedure (dilution)

Compounds **5d** (L-αMeVal) and **5e** (D-αMeVal) were dissolved in $CDCl_3$ until saturated solutions were formed. The initial concentration of each sample was measured using a known concentration of CH_2Cl_2 as an internal reference.

The maximum solubility of **5d** in chloroform-d was 288 mM for whereas the same value for its diastereomer **5e** was 50 mM.

Additional volumes of $CDCl_3$ were added to the initial solution and the ^{1}H NMR spectrum at each concentration was recorded.

9.2 Theory

The binding isotherms were generated using an Excel spreadsheet macro developed by Sanderson.[S7a] The Solver add-in for Excel was used to calculate the best fit to the NMR data using the 'GRG Nonlinear Solving Method'.

The following equilibrium is used to calculate the binding isotherms:

$$\mathrm{nH} \underset{}{\overset{K_{dim}}{\rightleftharpoons}} \mathrm{H_n}$$

Where K_{dim} is the microscopic association constant and **n** = 2.

The following equation is used to calculate the binding isotherm:

$$\delta_{obs} = \left[\frac{nK[\mathrm{H}]^n(\delta_{bnd} - \delta_{free})}{[\mathrm{H}]_0}\right] + \delta_{free}$$

where:

δ_{free} is the free chemical shift of the host

δ_{obs} is the observed chemical shift

δ_{bnd} is the limiting complexation-induced chemical shift range of the host in the dimeric complex

$[H]_0$ is the initial concentration of the host

[H] is the concentration of the free (uncomplexed) host

The concentration of the dimeric complex $(H)_2$ is calculated iteratively by solving the following equations:

$$[H_n] = \frac{(1 + 2nK[\mathrm{H}]_0) - \sqrt{(1 + 2nK[\mathrm{H}]_0)^2 - 4K^2n^2{[\mathrm{H}]_0}^2}}{2n^2K}$$

The concentration of free host is calculated according to the concentration difference

$$[\mathrm{H}] = [\mathrm{H}]_0 - n[\mathrm{H}_n]$$

9.3 Fitting

The propensity of the L-αMeVal-capped foldamer **5d** and D-αMeVal-capped foldamer **5e** to self-associate was investigated in chloroform by performing a dilution study,[S7] which at the same time quantified the solubility of these compounds in this solvent. We found marked differences in solubility, with a maximum solubility of 288 mM for **5d** whereas the same value for its diastereomer **5e** was 50 mM. However iterative fitting of the ^{1}H NMR spectroscopy dilution data to a simple dimerization model gave the same self-association constants within error, K_{dim} = 2 M^{-1}. These values are similar to those reported for other Aib tetramers by Pike *et al.*[S7b]

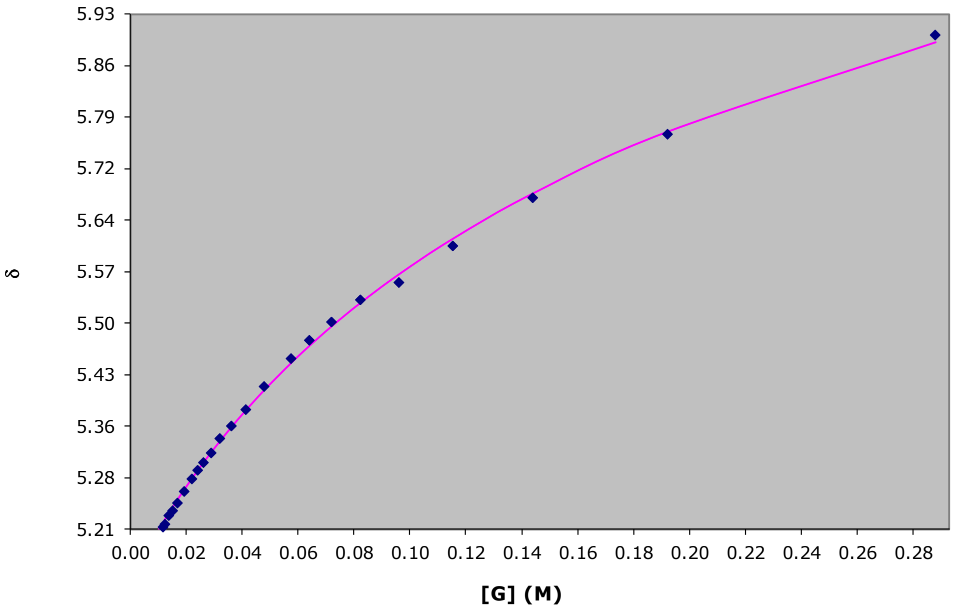

Figure S21 Plot of concentration (M) vs chemical shift (ppm) for an NH in foldamer **5d** (L-αMeVal) showing the observed data values (shown as blue diamonds) and the values calculated by the dimerization model programme (shown as a pink line) for K_{dim} = 2 M^{-1}.

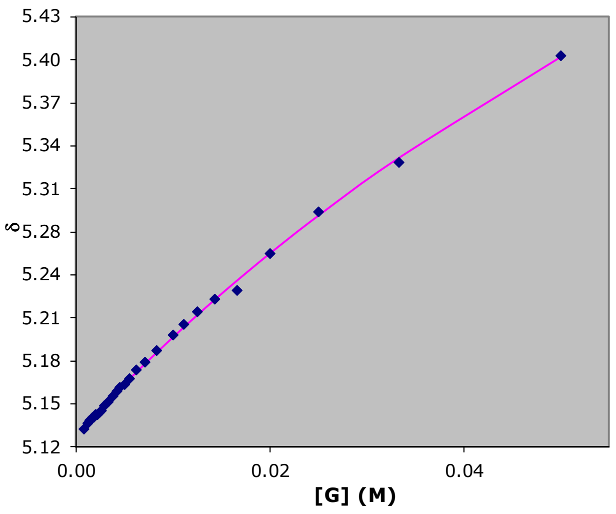

Figure S22 Plot of concentration (M) vs chemical shift (ppm) for an NH in foldamer **5e** (D-αMeVal) showing the observed data values (shown as blue diamonds) and the values calculated by the dimerization model programme (shown as a pink line) for K_{dim} = 2 M^{-1}.

10. Line shape analysis of VT NMR data

Line fitting was carried on the resonances between −78 and −82 ppm (small adjustments to the exact range for fitting were performed according to the temperature). The signals start to decoalesce at -30 °C. Fitting was performed using the DNMR module within TopSpin 4.1.1 (Bruker). The model had one spin system, which had two exchanging molecules (representing each screw-sense, *M* or *P*). The ratio was determined by fitting the spectrum at the lowest temperature (−40 °C). The line broadening was estimated by fitting the C_6F_6 reference peak at each temperature (LB typically from 8 to 30 Hz). The exchange constants (*k*) were initially estimated for each temperature then allowed to refine using the fitting programme (1000 steps or until convergence was reached). The final overlap values were acceptable, between 91.8% to 96.6% for fitting of the temperature data for Gly (**5a**) and were between 92.1% to 97.6% for fitting of the temperature data for L-Phe (**5b**).

10.1 Compound 5a (Gly)

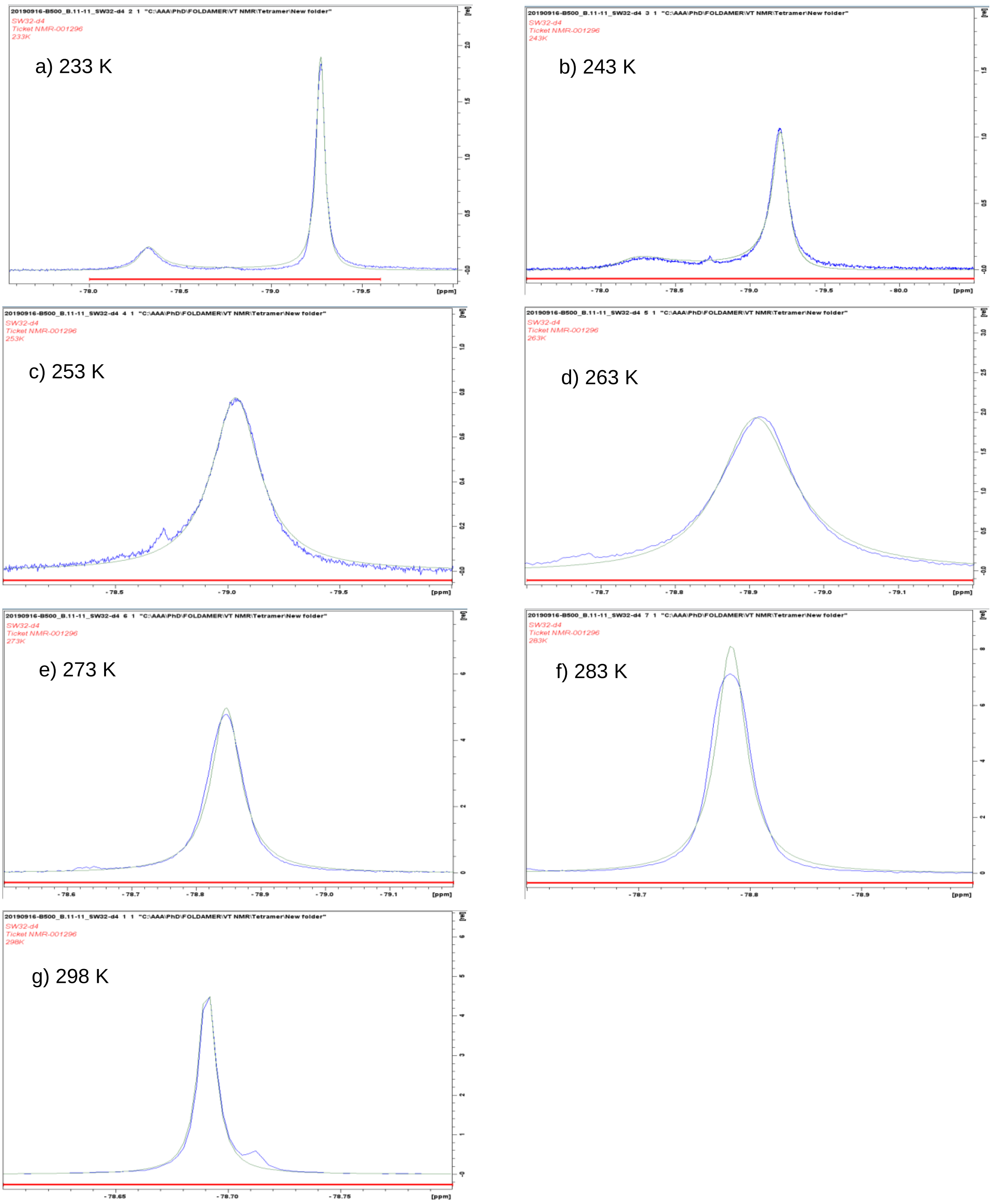

Figure S23 Fitted VT-NMR resonances for CF_3 of the reporter group on foldamer **5a** (Gly) from 233 to 298 K.

10.2 Compound 5b (L-Phe)

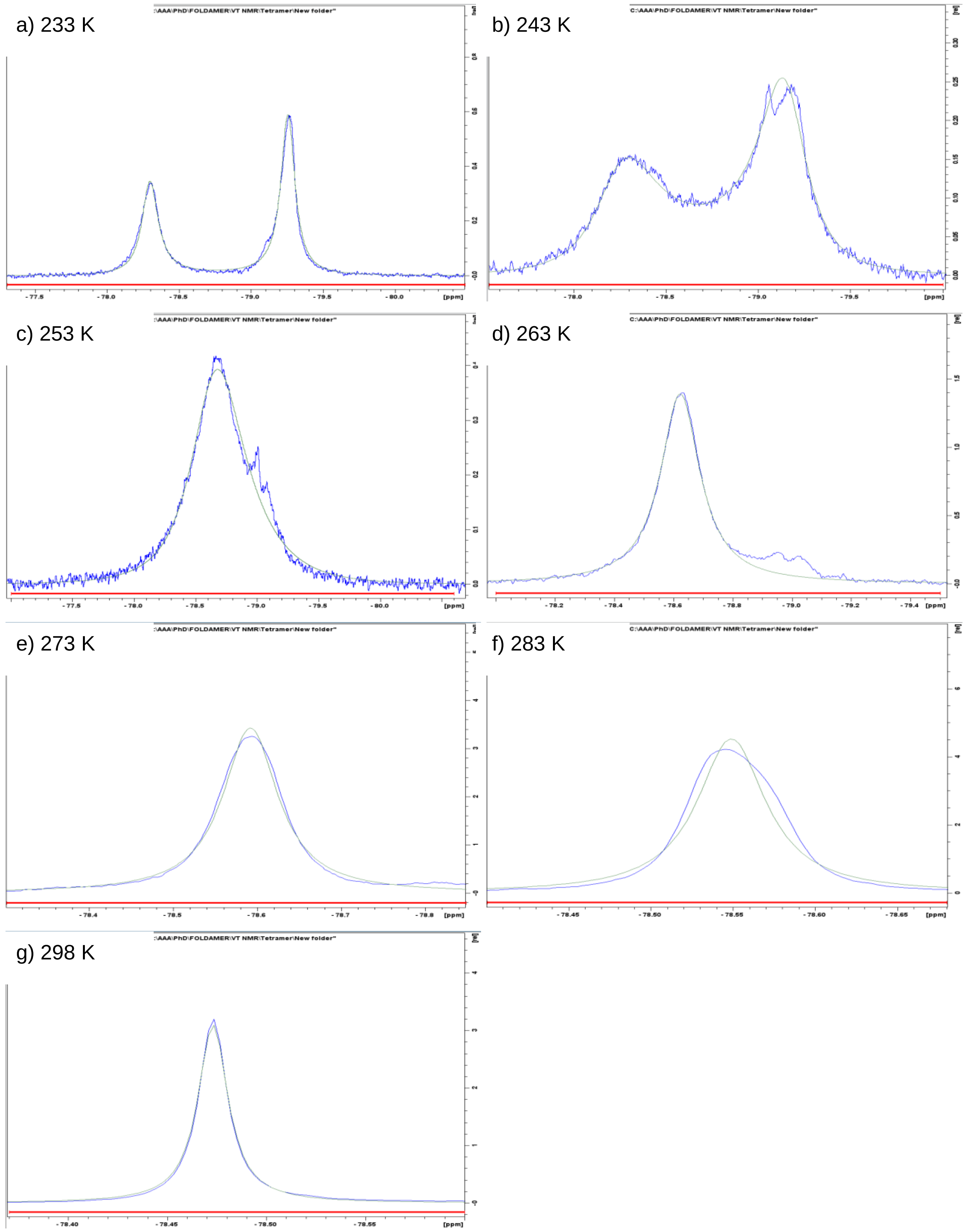

Figure S24 Fitted VT-NMR resonances for CF_3 of the reporter group on foldamer **5b** (L-Phe from 233 to 298 K).

11. Eyring plots

Line fitting data was performed using Topspin 4.1.4, which gave the *M*/*P* interchange rates (*k*) for **5a** and **5b**. The values of $\Delta H^{\ddagger}$, $\Delta G^{\ddagger}$ and $\Delta S^{\ddagger}$ for each foldamer were then calculated by solving the Eyring equation:

$$\ln\left(\frac{k}{T}\right) = -\frac{\Delta H^{\ddagger}}{R} \times \frac{1}{T} + \ln\frac{k_B}{h} + \frac{\Delta S^{\ddagger}}{R}$$

where: $\boldsymbol{k_B}$ is the Boltzmann constant; ***h*** is the Planck constant; ***T*** is the temperature; ***k*** is the *M*/*P* interchange rate constant; ***R*** is the gas constant.

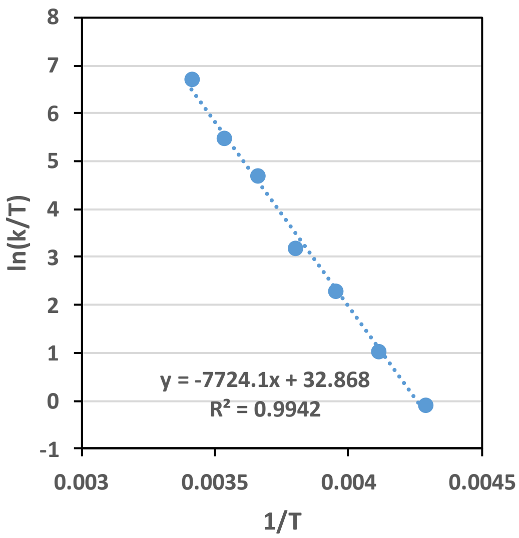

Figure S25 Eyring plot from the fitted VT-NMR data for foldamer **5a** (Gly).

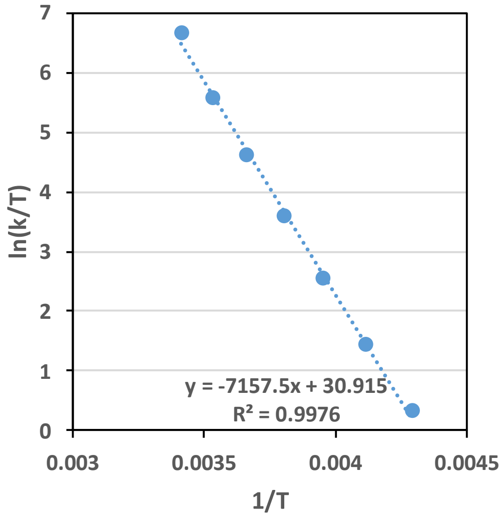

Figure S26 Eyring plot from the fitted VT-NMR data for foldamer **5b** (L-Phe).

The Eyring plots for foldamers **5a** and **5b** provided $\Delta H^{\ddagger}$, $\Delta S^{\ddagger}$ and $\Delta G^{\ddagger}$ for these compounds (all at 298 K), revealing that although there were some differences in $\Delta H^{\ddagger}$ and $\Delta S^{\ddagger}$ ($\Delta H^{\ddagger}$(**5a**) = +64 kJmol^{-1} and $\Delta H^{\ddagger}$(**5b**) = +60 kJmol^{-1}; $\Delta S^{\ddagger}$(**5a**) = +76 JK^{-1}mol^{-1} and $\Delta S^{\ddagger}$(**5b**) = +59 JK^{-1}mol^{-1}) the overall free energy change to reach the transition state, $\Delta G^{\ddagger}_{298}$ = +41 kJmol^{-1}, was identical within error. These data also show that helical

interconversion is enthalpically unfavourable but weakly entropically favoured ($T\Delta S^{‡}$ approx. −18 kJ mol^{-1}), a relationship also observed by Kubasik *et al* during their studies of *M*-*P* interconversion in Aib foldamers.[S14]

We suggest that the energy barrier for helical interconversion is similar across this family of tetrameric foldamers, as observed for a family of Aib foldamers Cbz(Xxx)Aib$_9$Ot-Bu (Xxx = amino acid residue) where $\Delta G^{‡}$ was in the range 35-39 kJ mol^{-1} for all members.[S15]

The measured $\Delta G^{‡}_{298}$ for foldamers **5a** and **5b** is very similar to that reported in the literature. Bodero *et al* estimated the energy barrier ($\Delta G^{‡}$) for the 3_{10}-helix interconversion of fluorinated foldamers Cbz-[(*R*)-TfmAla]Aib$_4$GlyNH$_2$ and Cbz-[(*S*)-TfmAla]Aib$_4$GlyNH$_2$ as ~ 46 kJmol^{-1} at 243 K.[S16] These foldamers have a similar length to the foldamers **5a** and **5b**, but show a slightly greater $\Delta G^{‡}$ as they have an extra hydrogen bond donor and acceptor. Similarly, longer foldamers FmocAib$_6$Ot-Bu and FmocAib$_8$Ot-Bu had $\Delta G^{‡}_{265}$ = 46 kJmol^{-1} and $\Delta G^{‡}_{265}$ = 48 kJmol^{-1} respectively.[S14]

As suggested by Kubasik *et al* we propose that interconversion occurs *via* the successive loss of a few hydrogen bonds rather than complete loss of helical structure, resulting in $\Delta G^{‡}$ being similar across **5a**-**5g** and **6**.[S14]

12. Crystal data and structure refinement

Data collection: X-ray diffraction data were collected for compounds **5b**, **5d**, **5e** and **6** on a dual source Rigaku FR-X rotating anode at 100 K with Cu-Kα (1.54184 Å) radiation, equipped with a Hypix000HE detector and Oxford cryosystem. X-ray data were collected using CrysAlisPro software.

Crystal structure determination and refinements: X-ray data were processed and reduced using CrysAlisPro. Absorption correction was performed using empirical methods (SCALE3 ABSPACK) based upon symmetry-equivalent reflections combined with measurements at different azimuthal angles. The crystal structure was solved and refined against all F^2 values using the SHELX and Olex2 suite of programmes.[S17-S19] All atoms were refined anisotropically. Hydrogen atoms were placed in calculated positions and refined using idealised geometries and assigned fixed isotropic displacement parameters.

Crystallographic data have been deposited with the CCDC (CCDC 2203280, 2203281, 2203282, 2203283).

12.1 Crystallographic information for compound 5b, 5d and 5e

Identification code	**5b**	**5d**	**5e**
Empirical formula	$C_{36}H_{49}F_3N_6O_7$	$C_{34}H_{53}Cl_2F_3N_6O_7$	$C_{33}H_{51}F_3N_6O_7$
Formula weight	734.81	785.72	700.79
Temperature/K	100.00(10)	99.98(10)	100.01(10)
Crystal system	orthorhombic	orthorhombic	orthorhombic
Space group	$P2_12_12_1$	$P2_12_12_1$	$P2_12_12$
a/Å	12.94029(15)	13.49460(10)	36.7966(4)
b/Å	13.58514(15)	13.61380(10)	11.14447(13)
c/Å	22.1112(3)	21.2661(2)	9.18889(12)
α/°	90	90	90
β/°	90	90	90
γ/°	90	90	90
Volume/Å^3	3887.05(8)	3906.85(5)	3768.17(8)
Z	4	4	4
ρ_{calc}g/cm^3	1.256	1.336	1.235
μ/mm^{-1}	0.816	2.071	0.813
F(000)	1560.0	1664.0	1496.0
Crystal size/mm^3	0.127 × 0.061 × 0.024	0.56 × 0.21 × 0.16	0.752 × 0.722 × 0.41
Radiation	CuKα (λ = 1.54184)	Cu Kα (λ = 1.54184)	Cu Kα (λ = 1.54184)
2Θ range for data collection/°	7.638 to 151.94	7.71 to 151.91	4.802 to 152.308
Index ranges	-16 ≤ h ≤ 16, -17 ≤ k ≤ 13, -19 ≤ l ≤ 27	-13 ≤ h ≤ 16, -17 ≤ k ≤ 13, -23 ≤ l ≤ 26	-35 ≤ h ≤ 43, -13 ≤ k ≤ 13, -11 ≤ l ≤ 11
Reflections collected	24766	21409	20598
Independent reflections	7887 [R_{int} = 0.0225, R_{sigma} = 0.0250]	7776 [R_{int} = 0.0223, R_{sigma} = 0.0228]	7568 [R_{int} = 0.0188, R_{sigma} = 0.0151]
Data/restraints/parameters	7887/0/502	7776/0/482	7568/0/473
Goodness-of-fit on F^2	1.056	1.025	1.066
Final R indexes [I>=2σ (I)]	R_1 = 0.0294, wR_2 = 0.0739	R_1 = 0.0271, wR_2 = 0.0692	R_1 = 0.0286, wR_2 = 0.0737
Final R indexes [all data]	R_1 = 0.0324, wR_2 = 0.0753	R_1 = 0.0275, wR_2 = 0.0695	R_1 = 0.0287, wR_2 = 0.0738
Largest diff. peak/hole / e Å^{-3}	0.16/-0.18	0.63/-0.34	0.19/-0.15
Flack parameter	-0.07(5)	0.007(4)	-0.03(4)

12.2 Crystallographic information for compound 6

Identification code	**6**
Empirical formula	$C_{19}H_{32}F_3N_7O_4$
Formula weight	479.51
Temperature/K	100.00(10)
Crystal system	triclinic
Space group	P1
a/Å	9.06078(17)
b/Å	10.2310(3)
c/Å	15.4583(2)
α/°	91.6204(16)
β/°	105.5466(15)
γ/°	115.414(2)
Volume/Å^3	1229.95(5)
Z	2
ρ_{calc}g/cm^3	1.295
μ/mm^{-1}	0.919
F(000)	508.0
Crystal size/mm^3	0.237 × 0.171 × 0.055
Radiation	Cu Kα (λ = 1.54184)
2Θ range for data collection/°	6.016 to 150.622
Index ranges	-11 ≤ h ≤ 11, -12 ≤ k ≤ 12, -19 ≤ l ≤ 19
Reflections collected	23874
Independent reflections	9156 [R_{int} = 0.0203, R_{sigma} = 0.0258]
Data/restraints/parameters	9156/3/641
Goodness-of-fit on F^2	1.040
Final R indexes [I>=2σ (I)]	R_1 = 0.0230, wR_2 = 0.0591
Final R indexes [all data]	R_1 = 0.0235, wR_2 = 0.0593
Largest diff. peak/hole / e Å^{-3}	0.17/-0.19
Flack parameter	-0.03(3)

13. NMR spectra of new compounds

13.1 Cbz(Gly)Aib$_4$(*R*-TFEA) (5a)

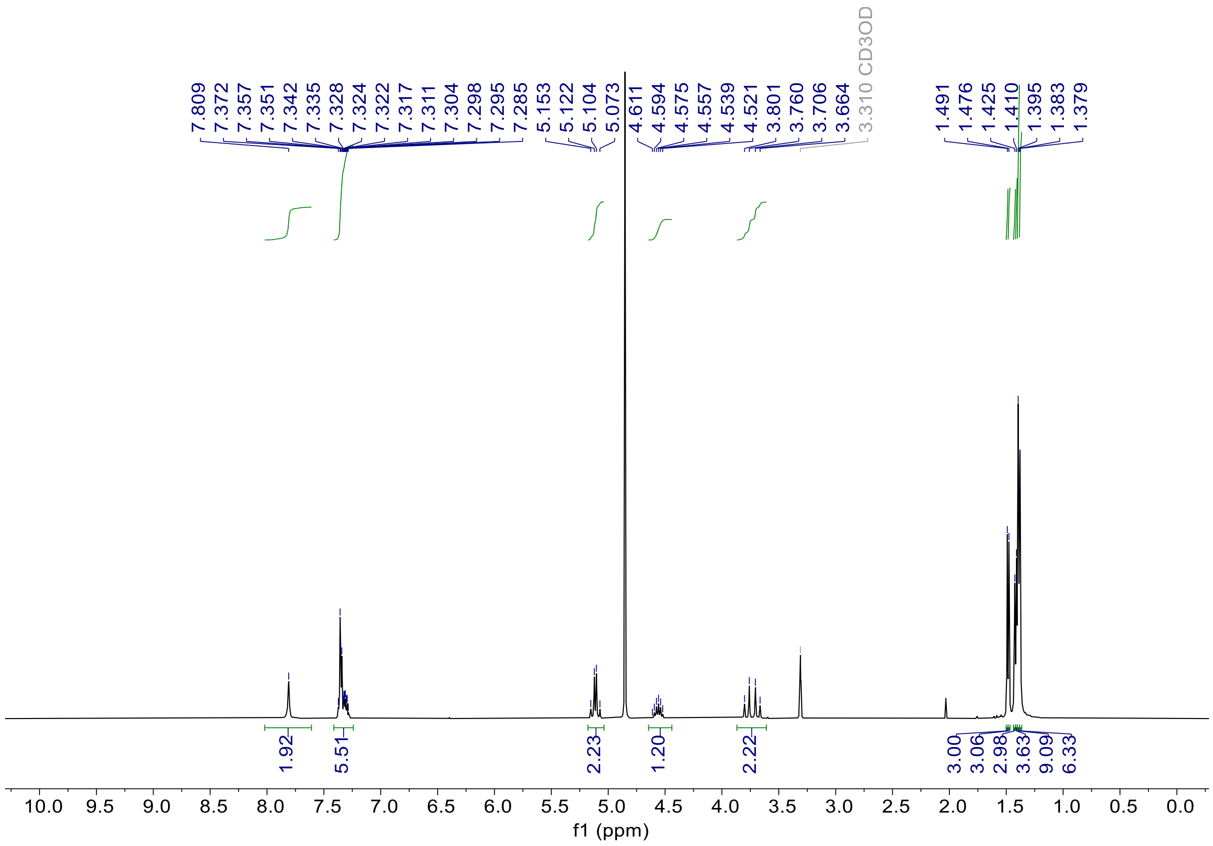

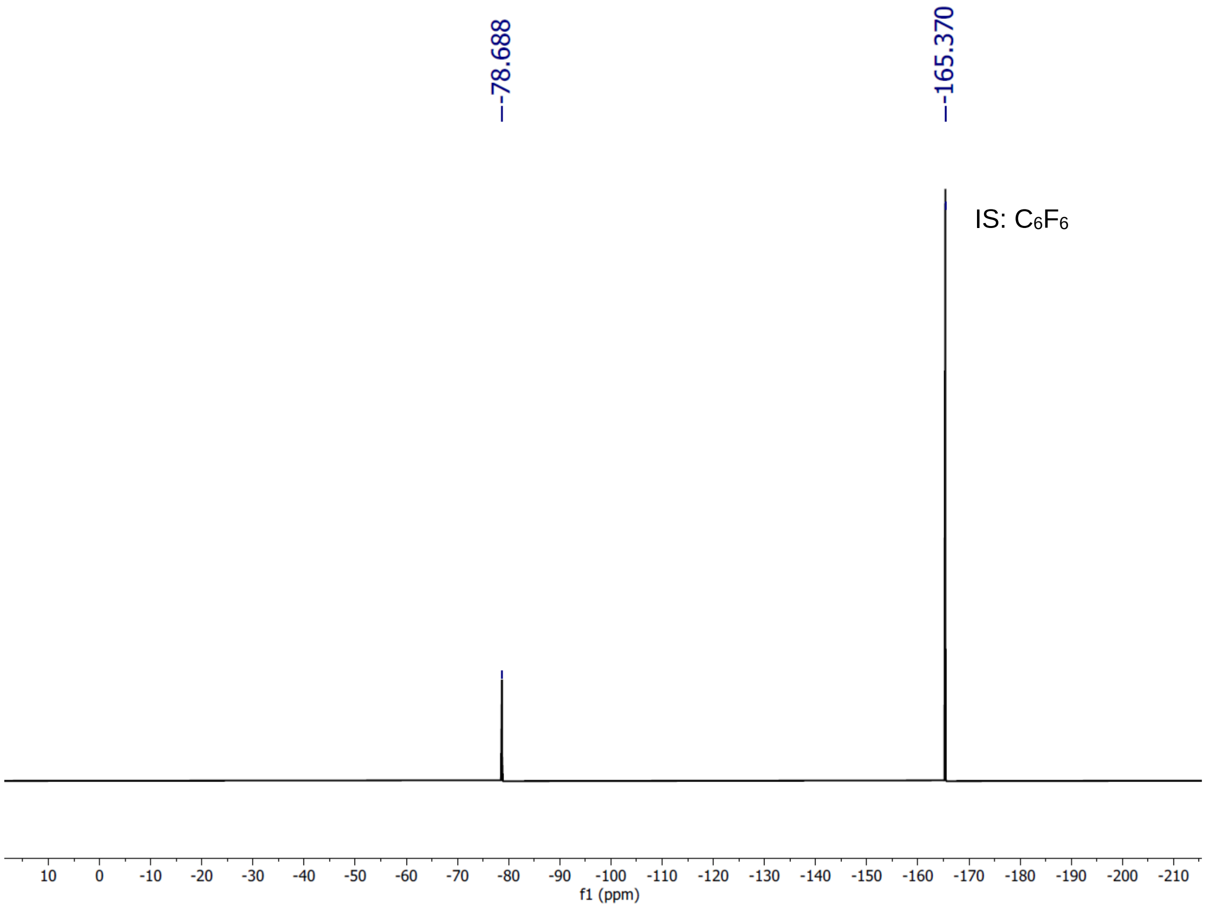

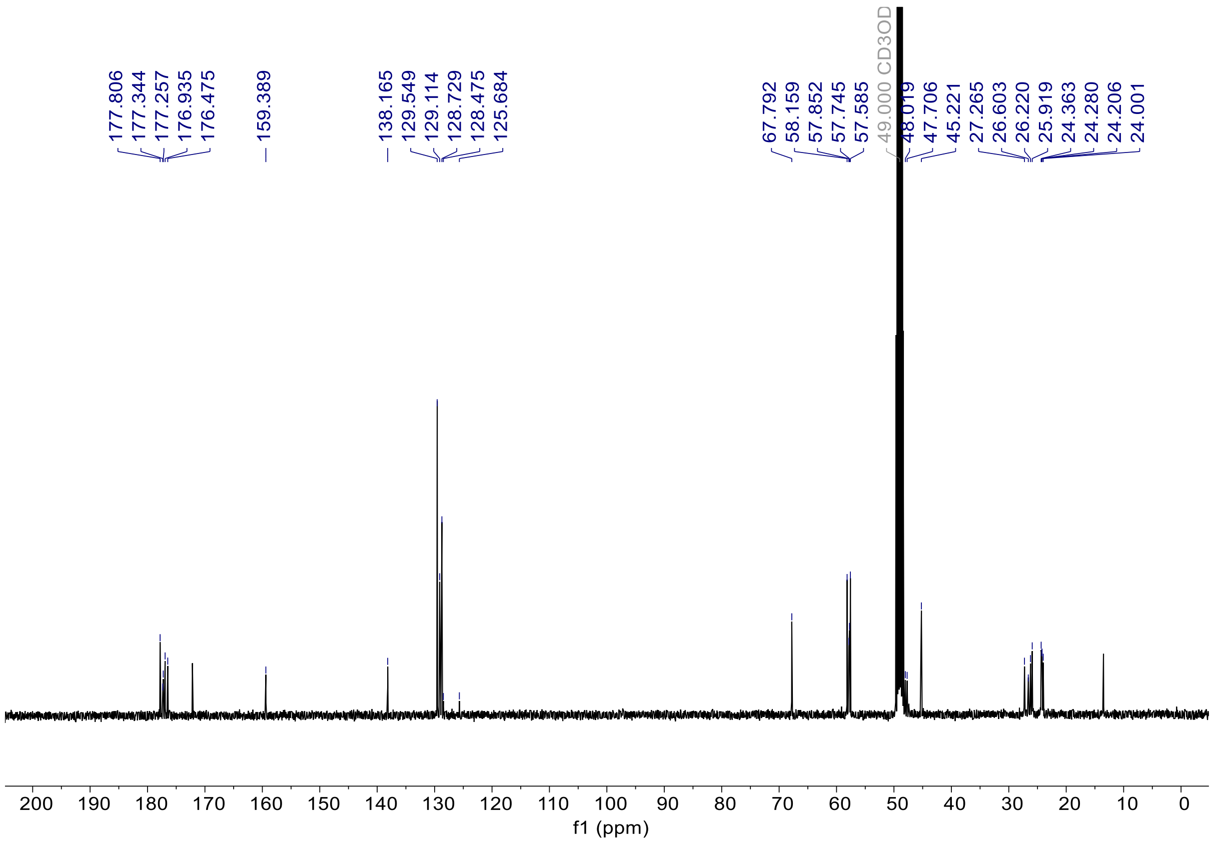

177.806
177.344
177.257
176.935
176.475
159.389
138.165
129.549
129.114
128.729
128.475
125.684
67.792
58.159
57.852
57.745
57.585
49.000 CD3OD
48.019
47.706
45.221
27.265
26.603
26.220
25.919
24.363
24.280
24.206
24.001
200
190
180
170
160
150
140
130
120
110
100
90
80
70
60
50
40
30
20
10
0
f1 (ppm)

13.2 Cbz(L-Phe)Aib$_4$(*R*-TFEA) (5b)

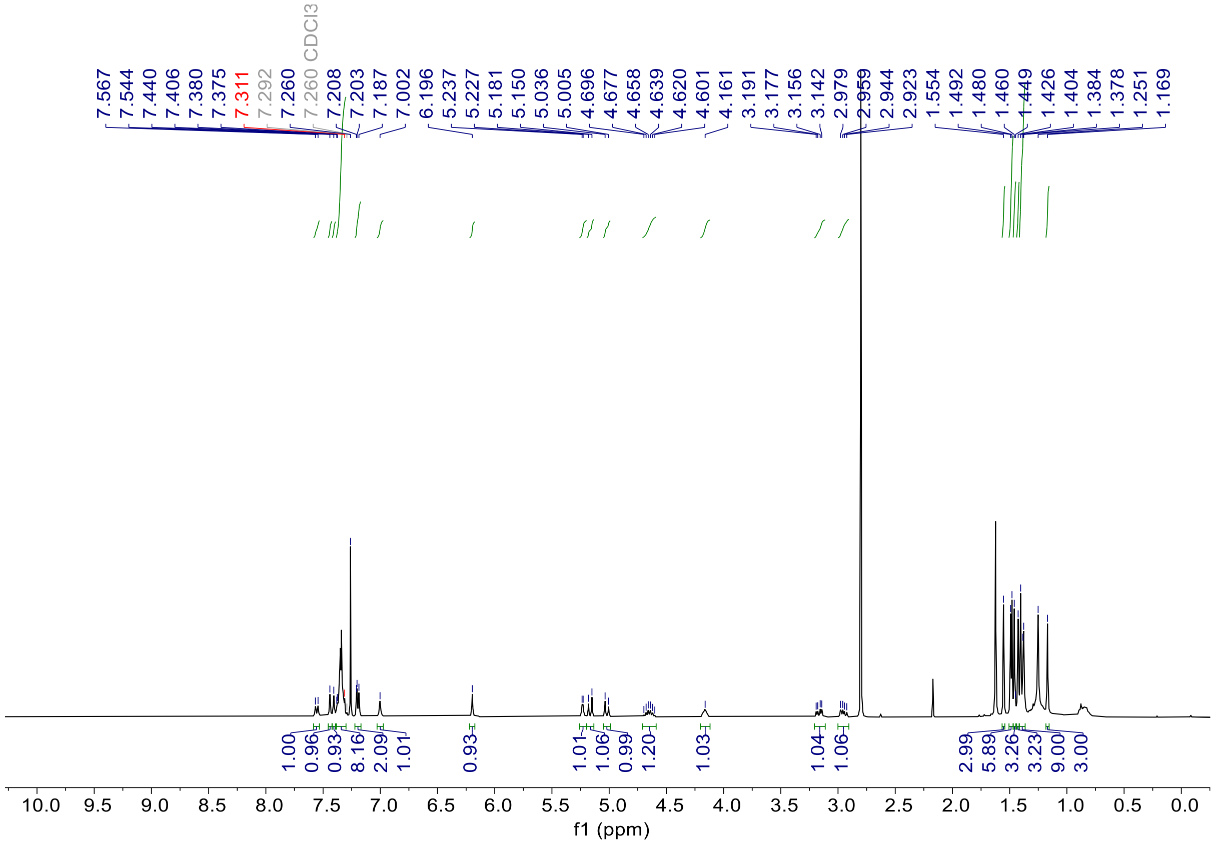

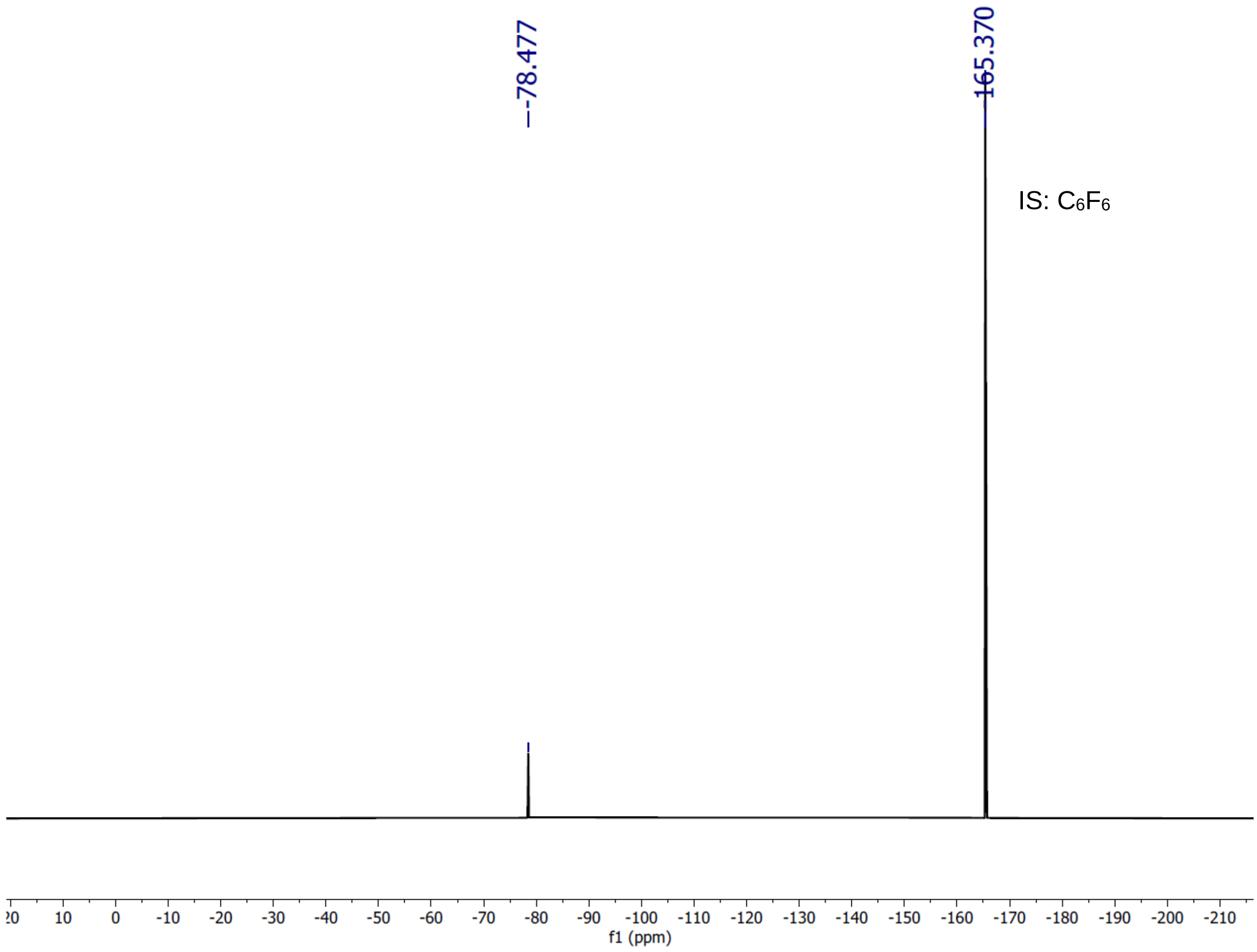

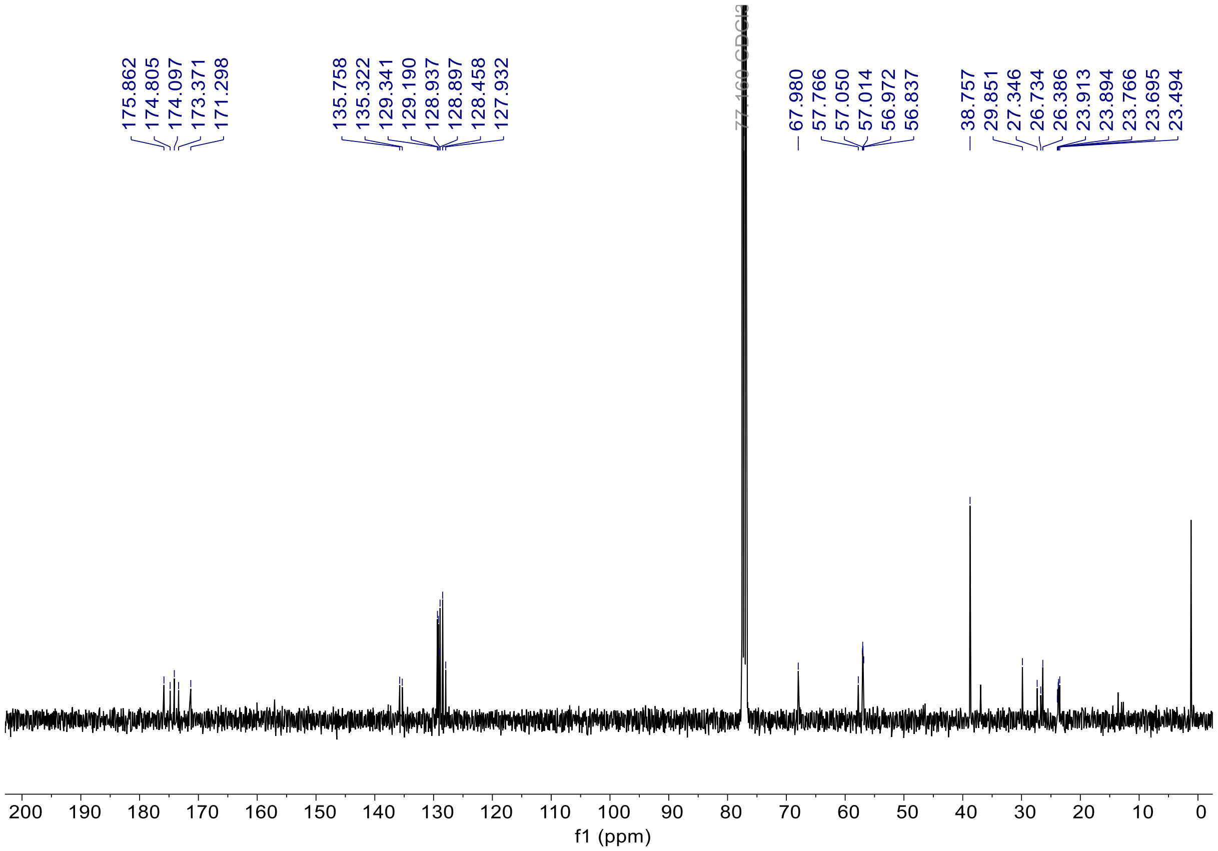
175.862
174.805
174.097
173.371
171.298
135.758
135.322
129.341
129.190
128.937
128.897
128.458
127.932
77.160 CDCl3
67.980
57.766
57.050
57.014
56.972
56.837
38.757
29.851
27.346
26.734
26.386
23.913
23.894
23.766
23.695
23.494
200 190 180 170 160 150 140 130 120 110 100 90 80 70 60 50 40 30 20 10 0
f1 (ppm)

13.3 Cbz(D-Phe)Aib$_4$(R-TFEA) (5c)

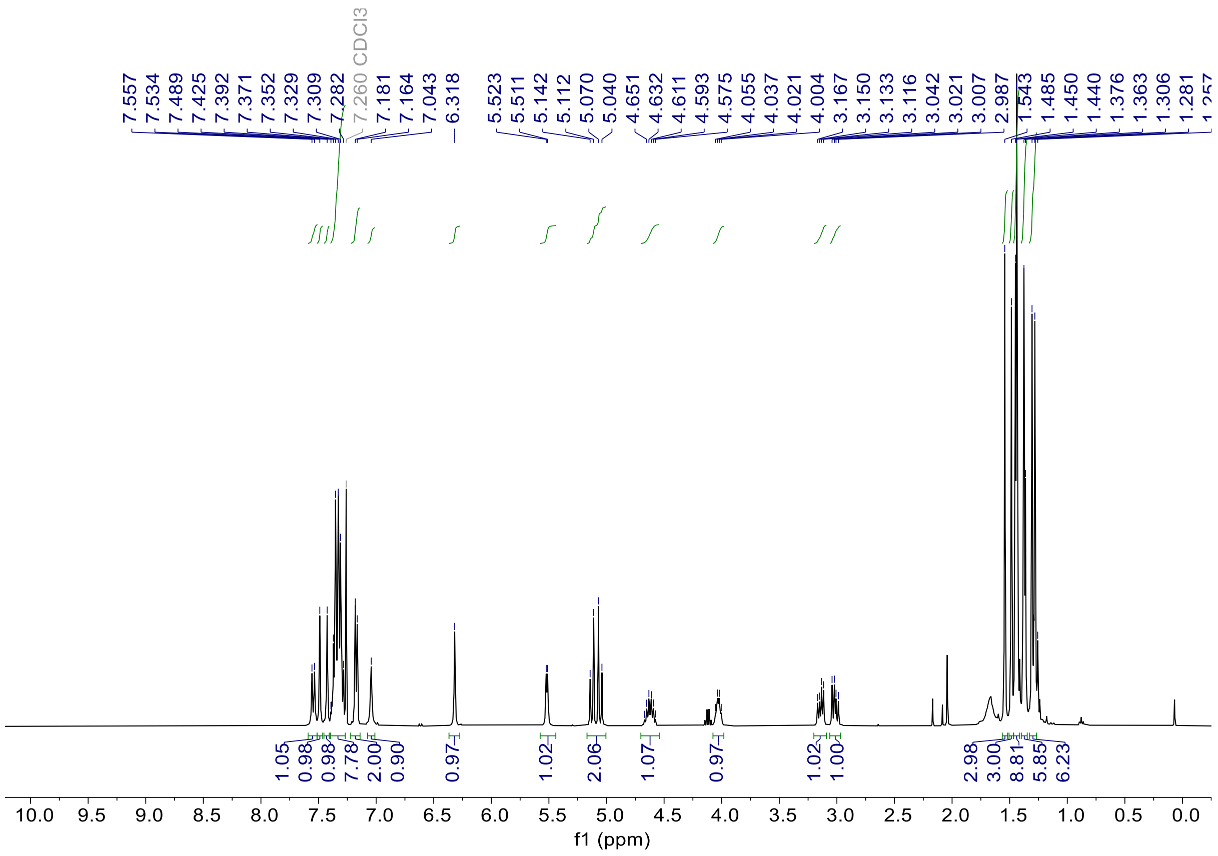

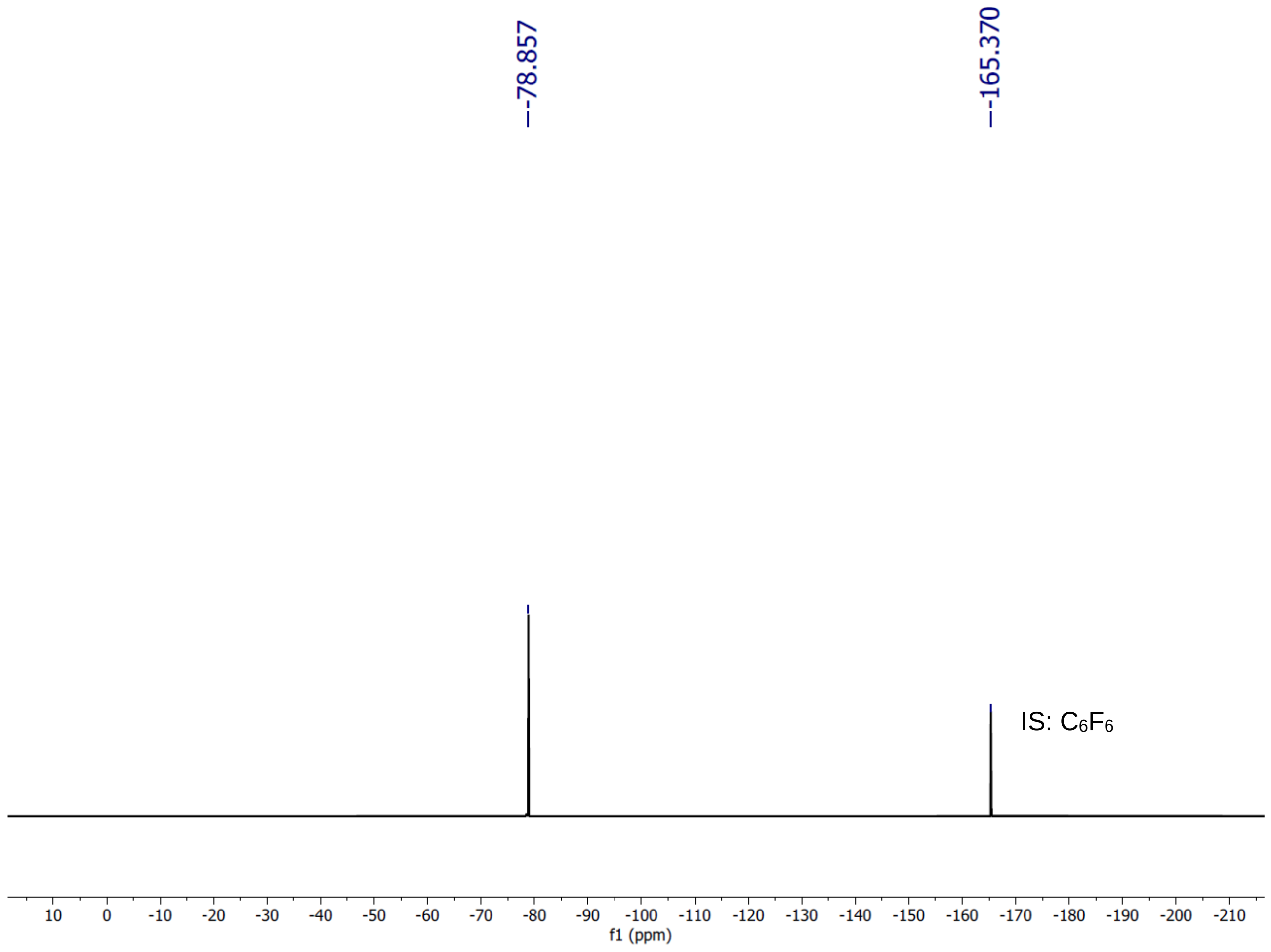

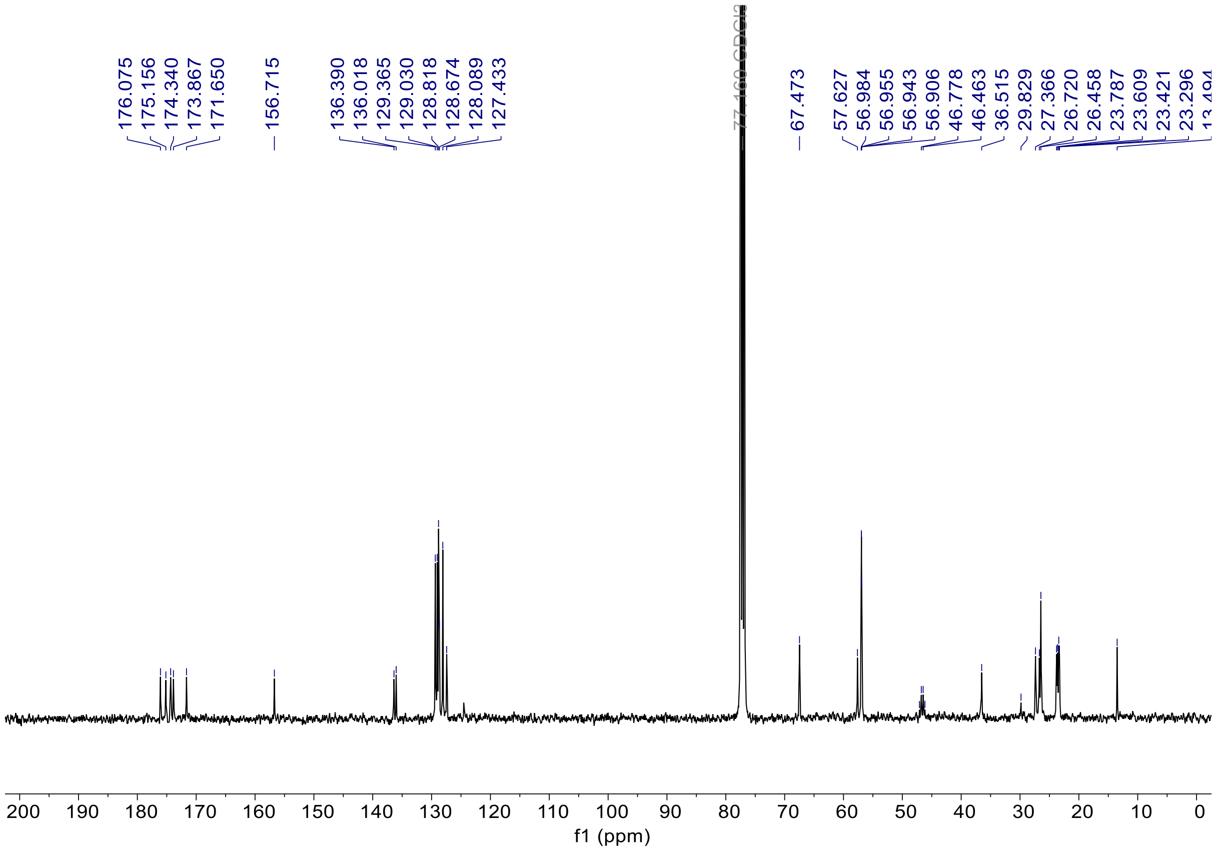
176.075
175.156
174.340
173.867
171.650
156.715
136.390
136.018
129.365
129.030
128.818
128.674
128.089
127.433
77.160 CDCl3
67.473
57.627
56.984
56.955
56.943
56.906
46.778
46.463
36.515
29.829
27.366
26.720
26.458
23.787
23.609
23.421
23.296
200 190 180 170 160 150 140 130 120 110 100 90 80 70 60 50 40 30 20 10 0
f1 (ppm)

13.4 Cbz(L-αMeVal)Aib$_4$(*R*-TFEA) (5d)

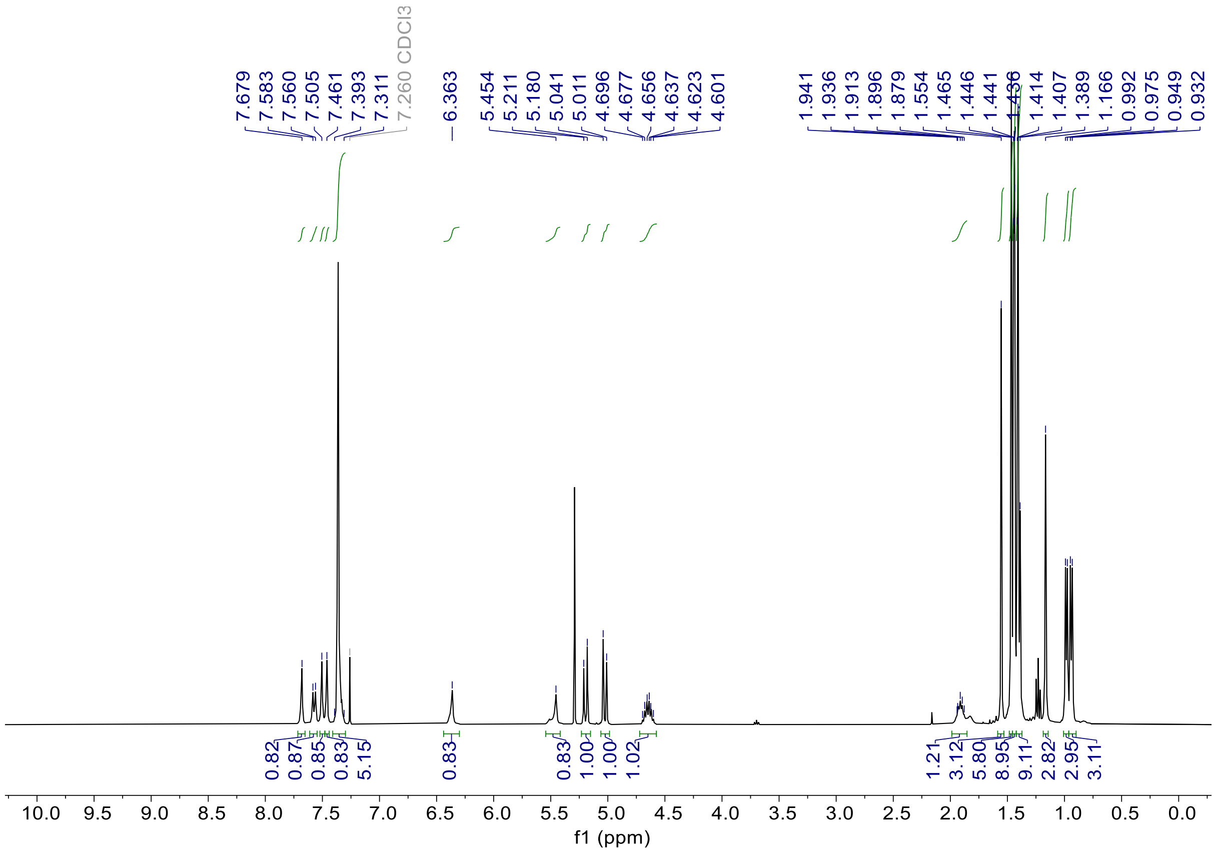

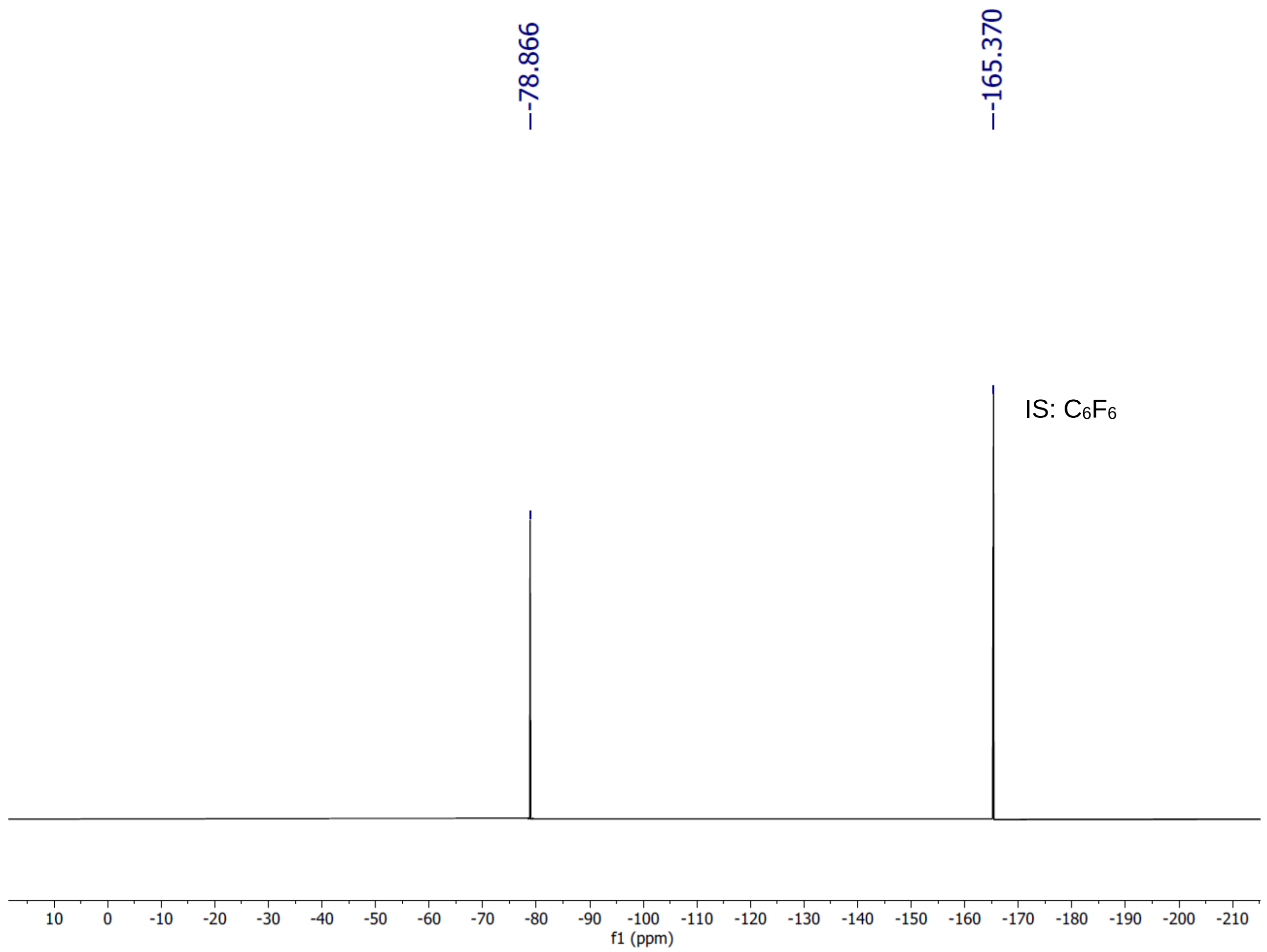

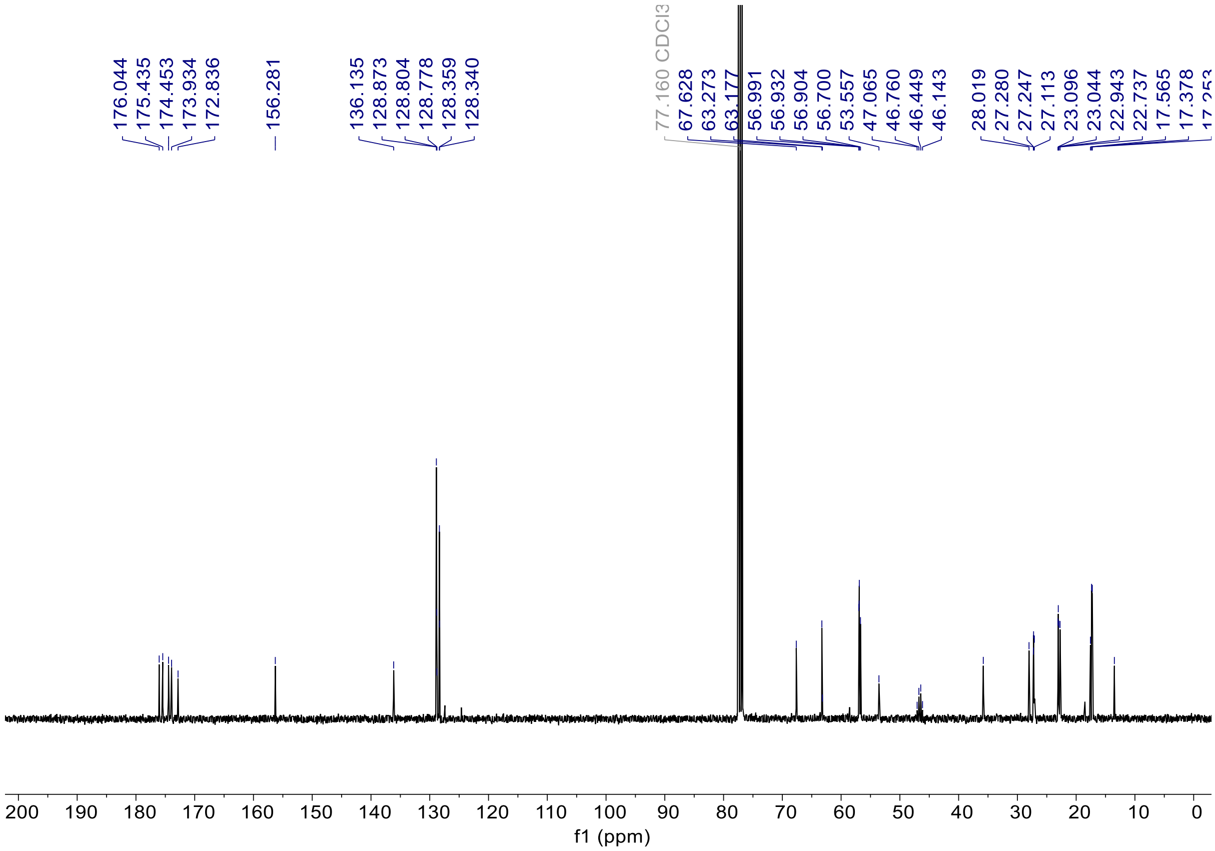
176.044
175.435
174.453
173.934
172.836
156.281
136.135
128.873
128.804
128.778
128.359
128.340
77.160 CDCl3
67.628
63.273
63.177
56.991
56.932
56.904
56.700
53.557
47.065
46.760
46.449
46.143
28.019
27.280
27.247
27.113
23.096
23.044
22.943
22.737
17.565
17.378
200 190 180 170 160 150 140 130 120 110 100 90 80 70 60 50 40 30 20 10 0
f1 (ppm)

13.5 Cbz(D-αMeVal)Aib$_4$(*R*-TFEA) (5e)

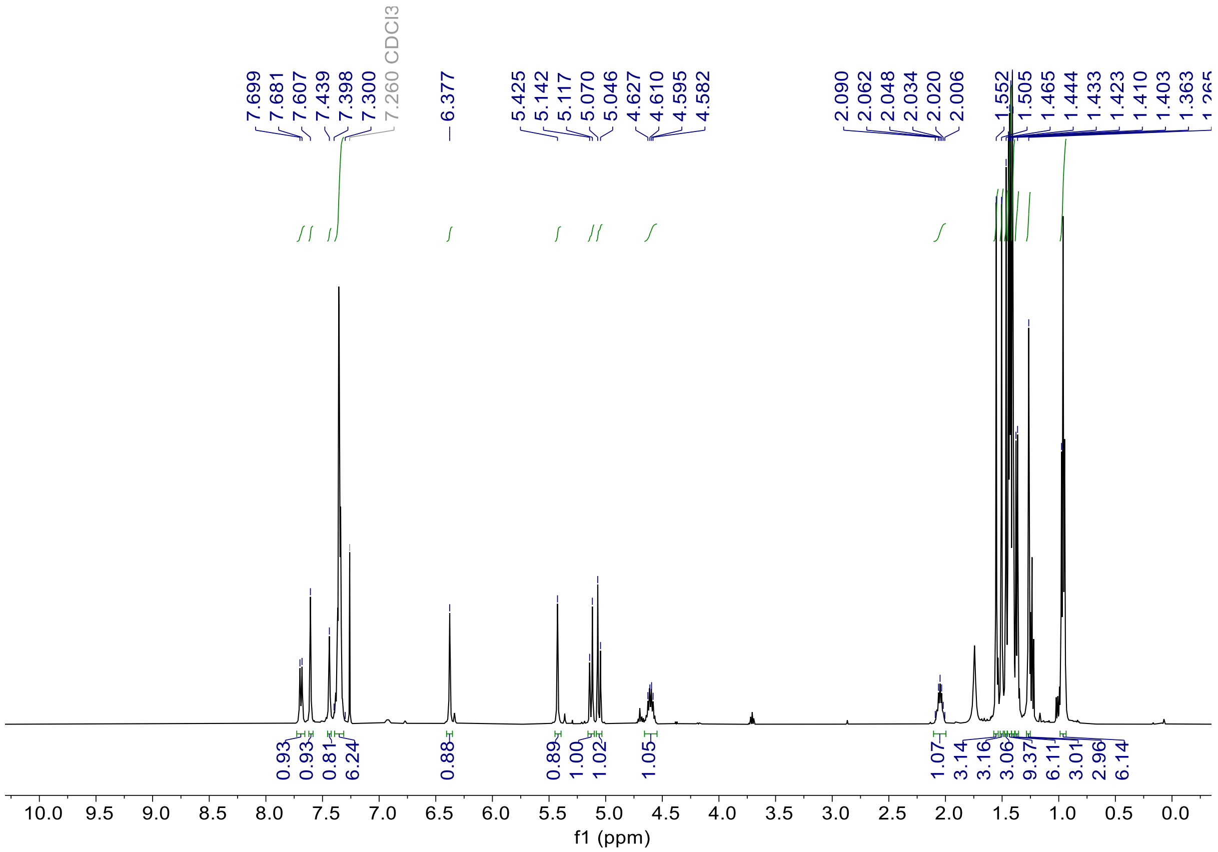

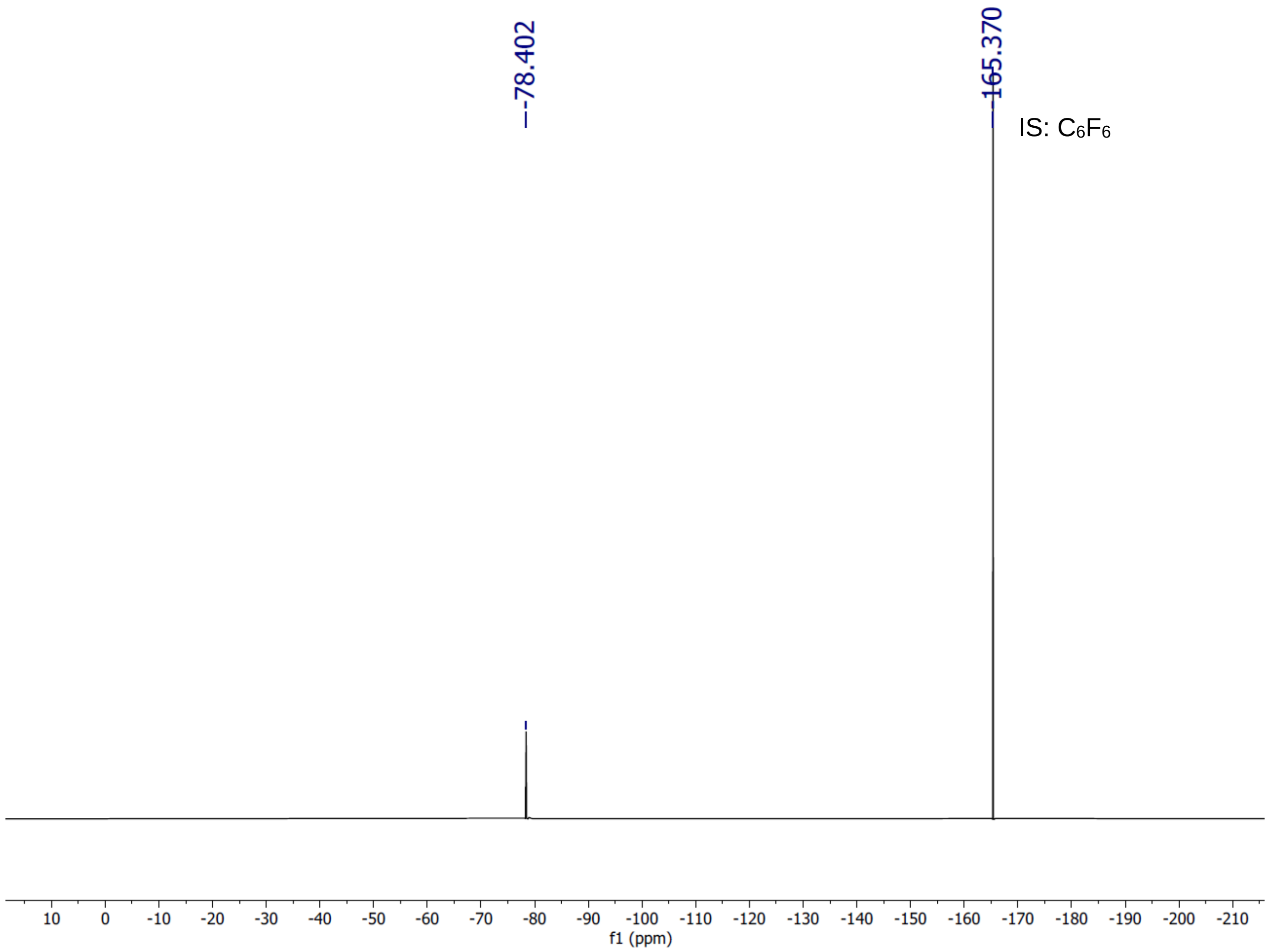

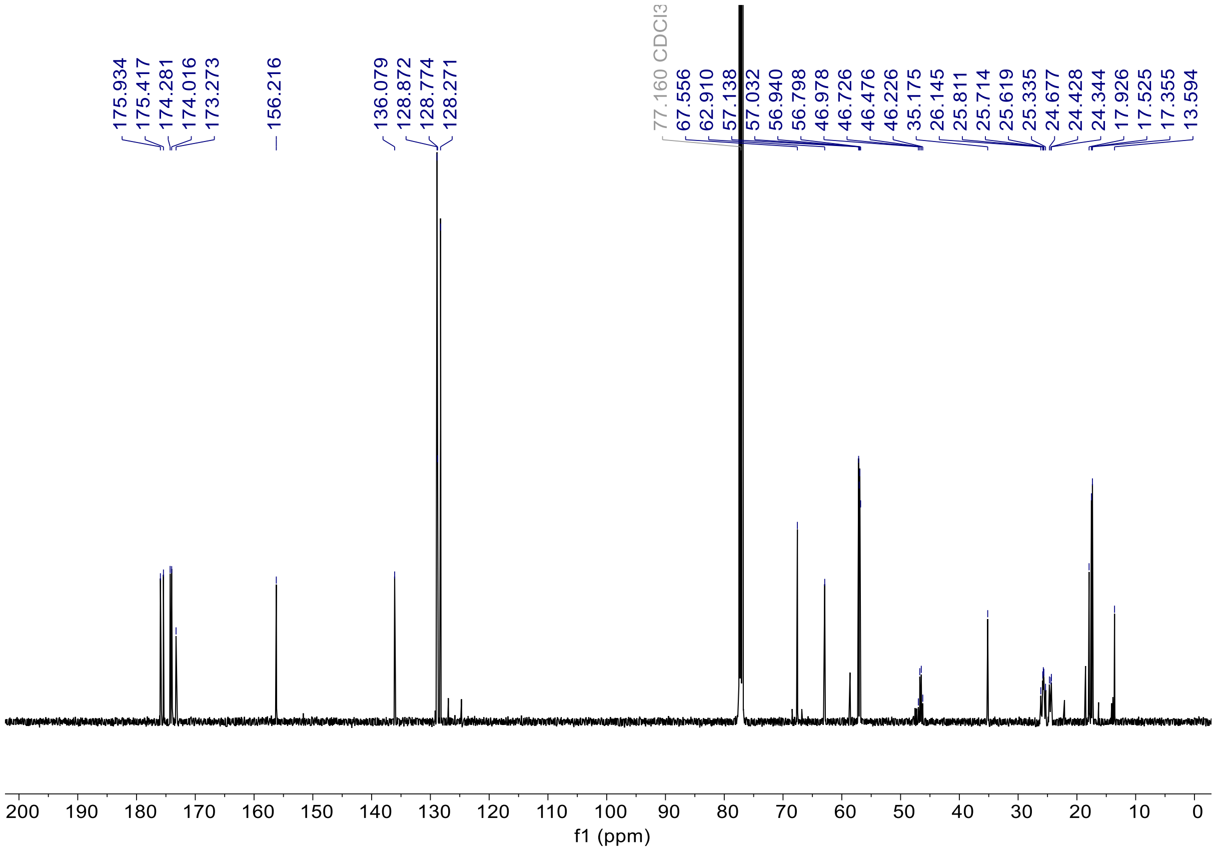
175.934
175.417
174.281
174.016
173.273
156.216
136.079
128.872
128.774
128.271
77.160 CDCl3
67.556
62.910
57.138
57.032
56.940
56.798
46.978
46.726
46.476
46.226
35.175
26.145
25.811
25.714
25.619
25.335
24.677
24.428
24.344
17.926
17.525
17.355
13.594
200 190 180 170 160 150 140 130 120 110 100 90 80 70 60 50 40 30 20 10 0
f1 (ppm)

13.6 Cbz(L-αMeVal)$_2$Aib$_4$(*R*-TFEA) (5f)

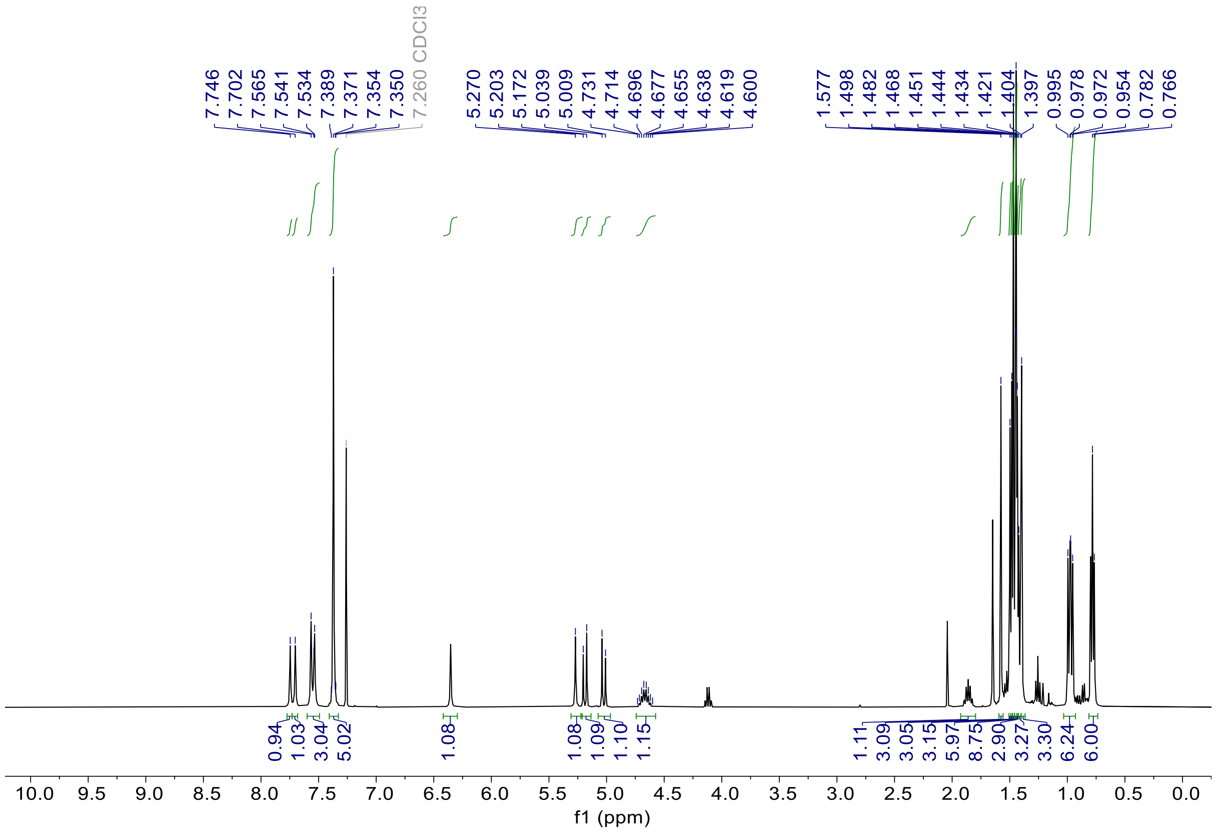

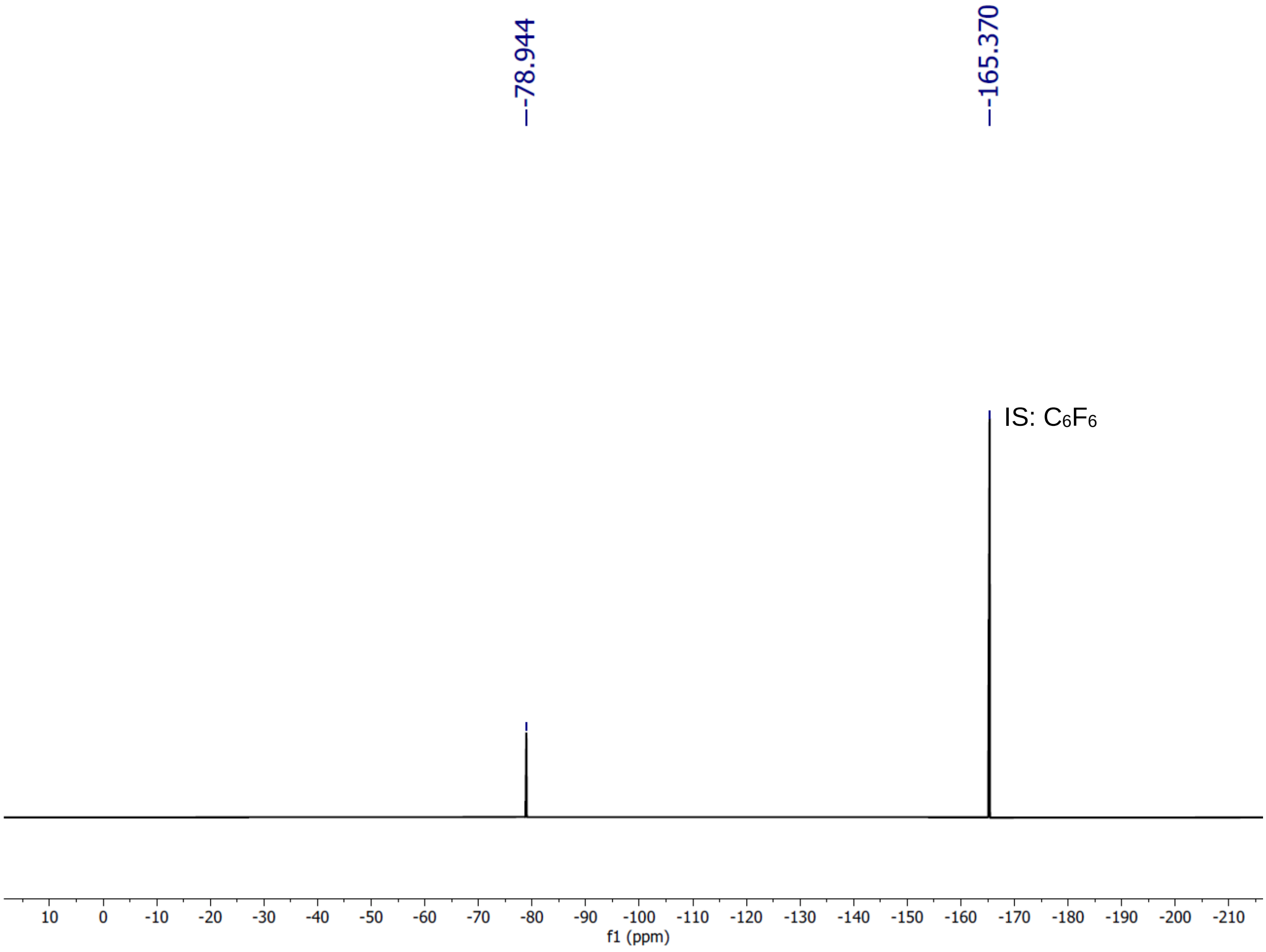

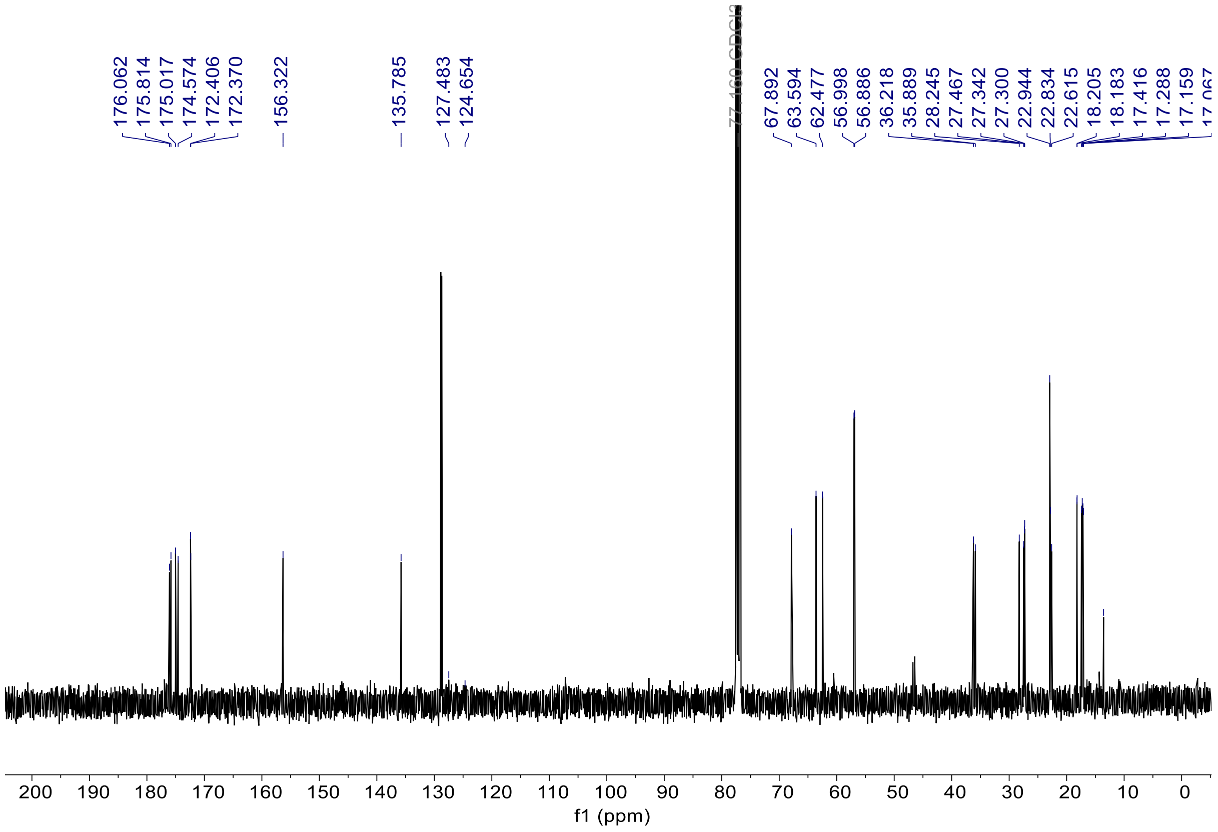
176.062
175.814
175.017
174.574
172.406
172.370
156.322
135.785
127.483
124.654
67.892
63.594
62.477
56.998
56.886
36.218
35.889
28.245
27.467
27.342
27.300
22.944
22.834
22.615
18.205
18.183
17.416
17.288
17.159
200 190 180 170 160 150 140 130 120 110 100 90 80 70 60 50 40 30 20 10 0
f1 (ppm)

13.7 Cbz(D-αMeVal)$_2$Aib$_4$(*R*-TFEA) (5g)

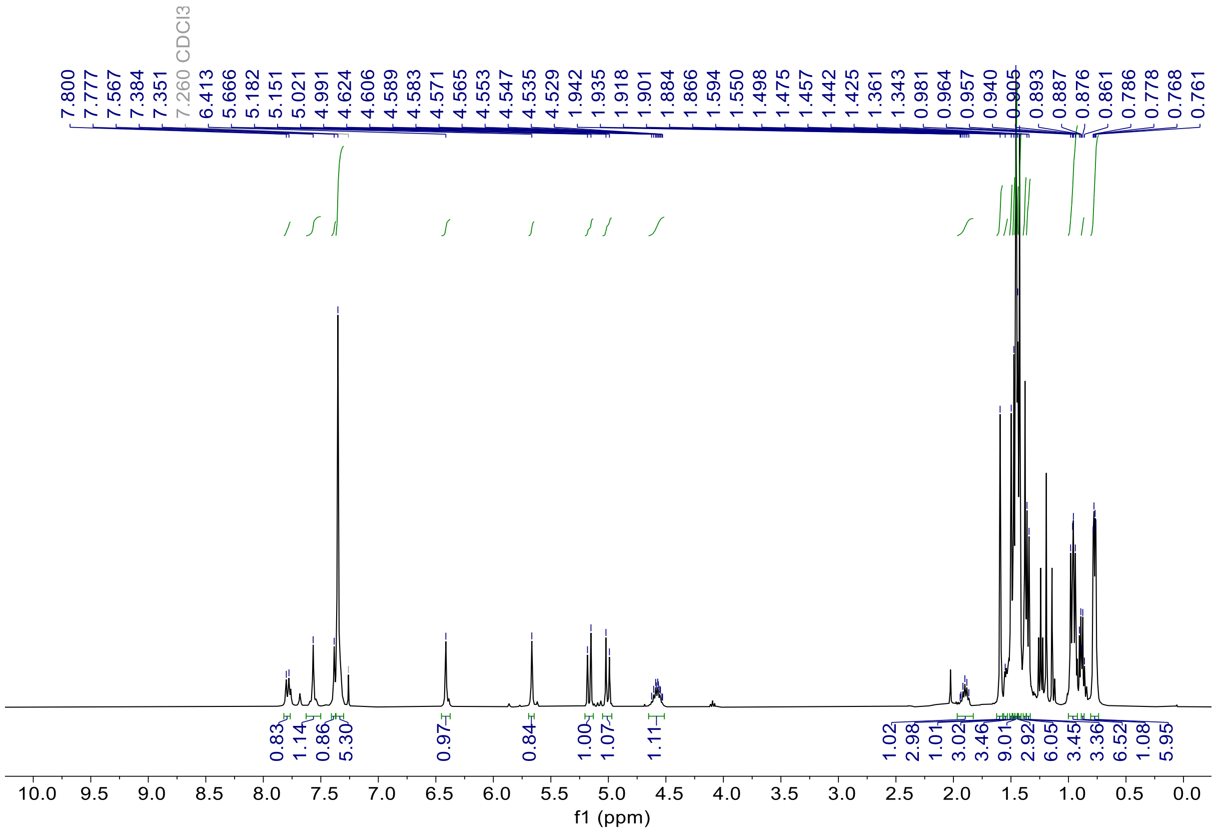

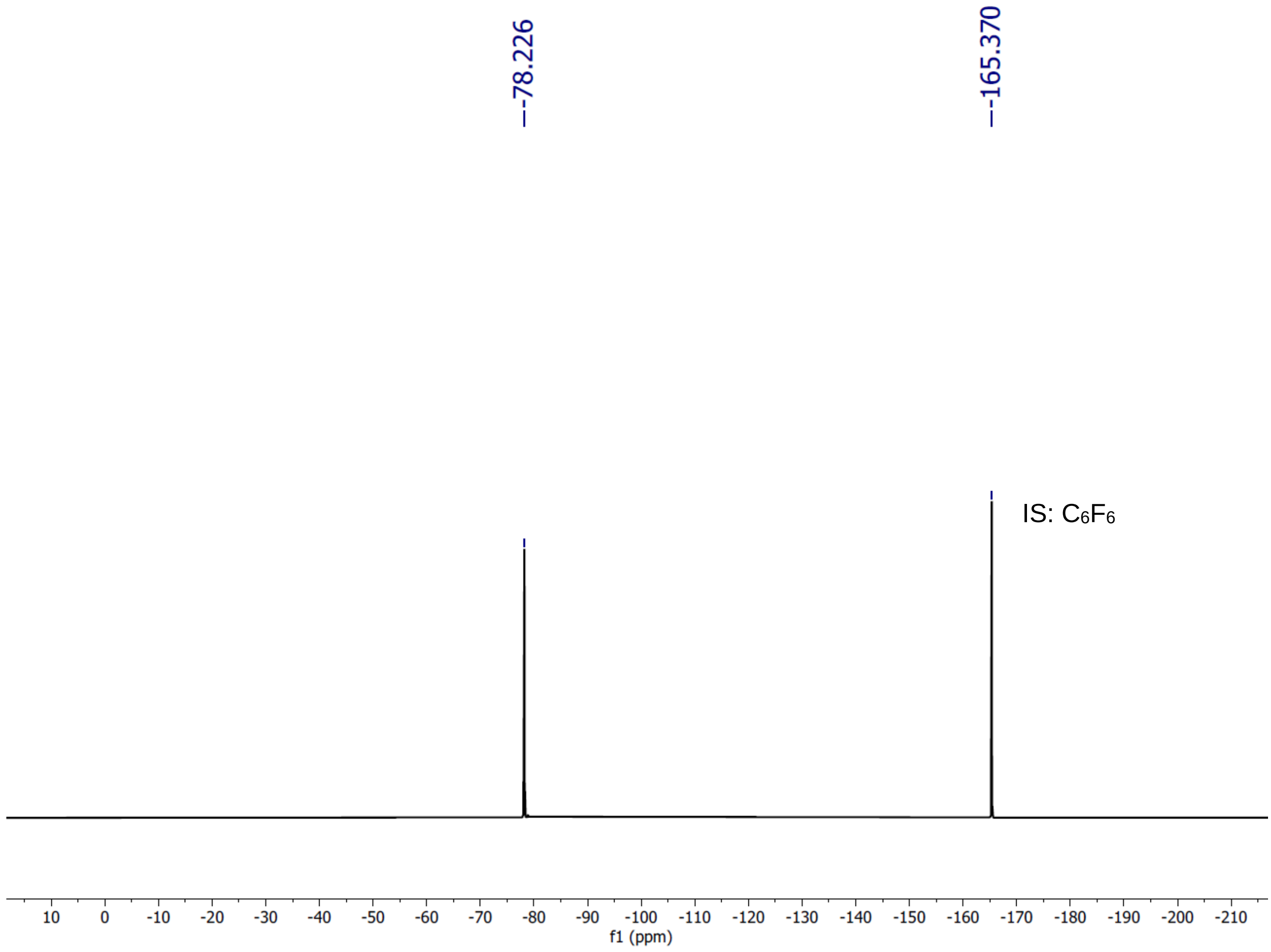

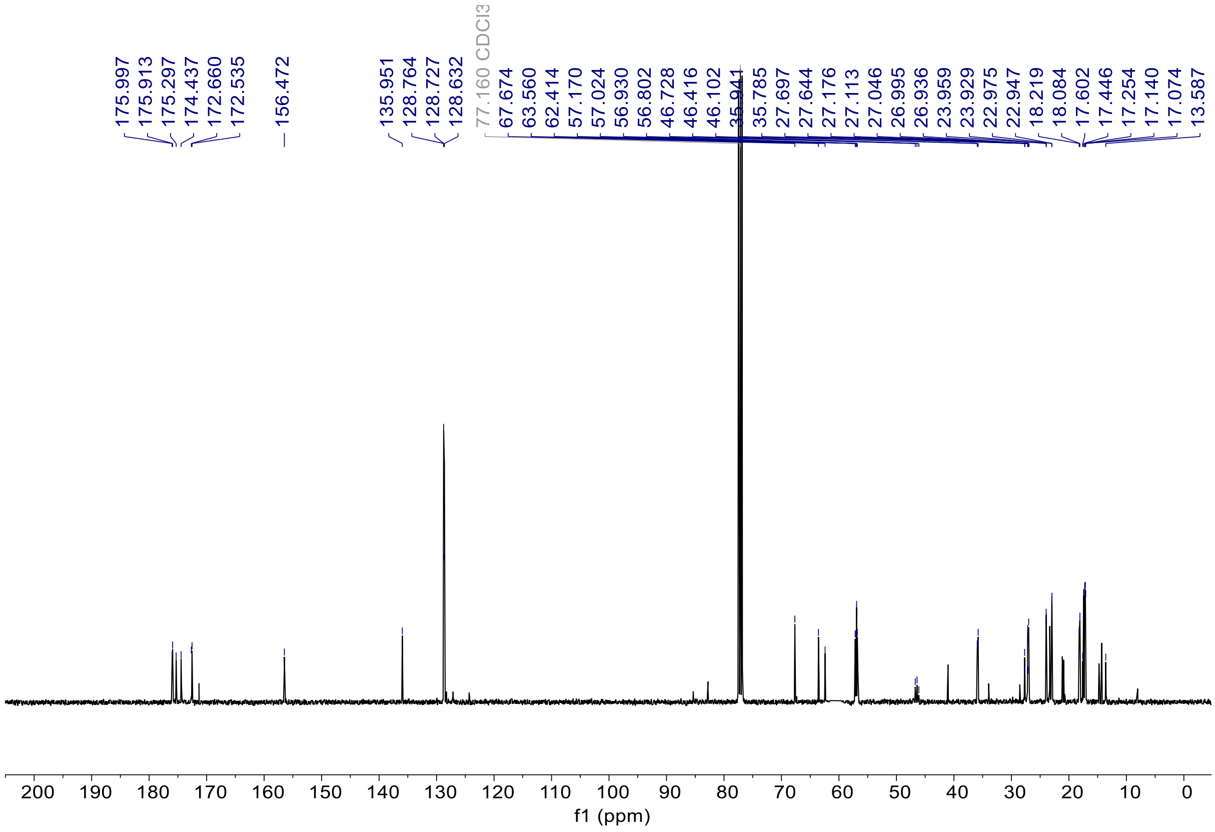
175.997
175.913
175.297
174.437
172.660
172.535
156.472
135.951
128.764
128.727
128.632
77.160 CDCl3
67.674
63.560
62.414
57.170
57.024
56.930
56.802
46.728
46.416
46.102
35.941
35.785
27.697
27.644
27.176
27.113
27.046
26.995
26.936
23.959
23.929
22.975
22.947
18.219
18.084
17.602
17.446
17.254
17.140
17.074
13.587
200 190 180 170 160 150 140 130 120 110 100 90 80 70 60 50 40 30 20 10 0
f1 (ppm)

13.8 N_3Aib_4(*R*-TFEA) (6)

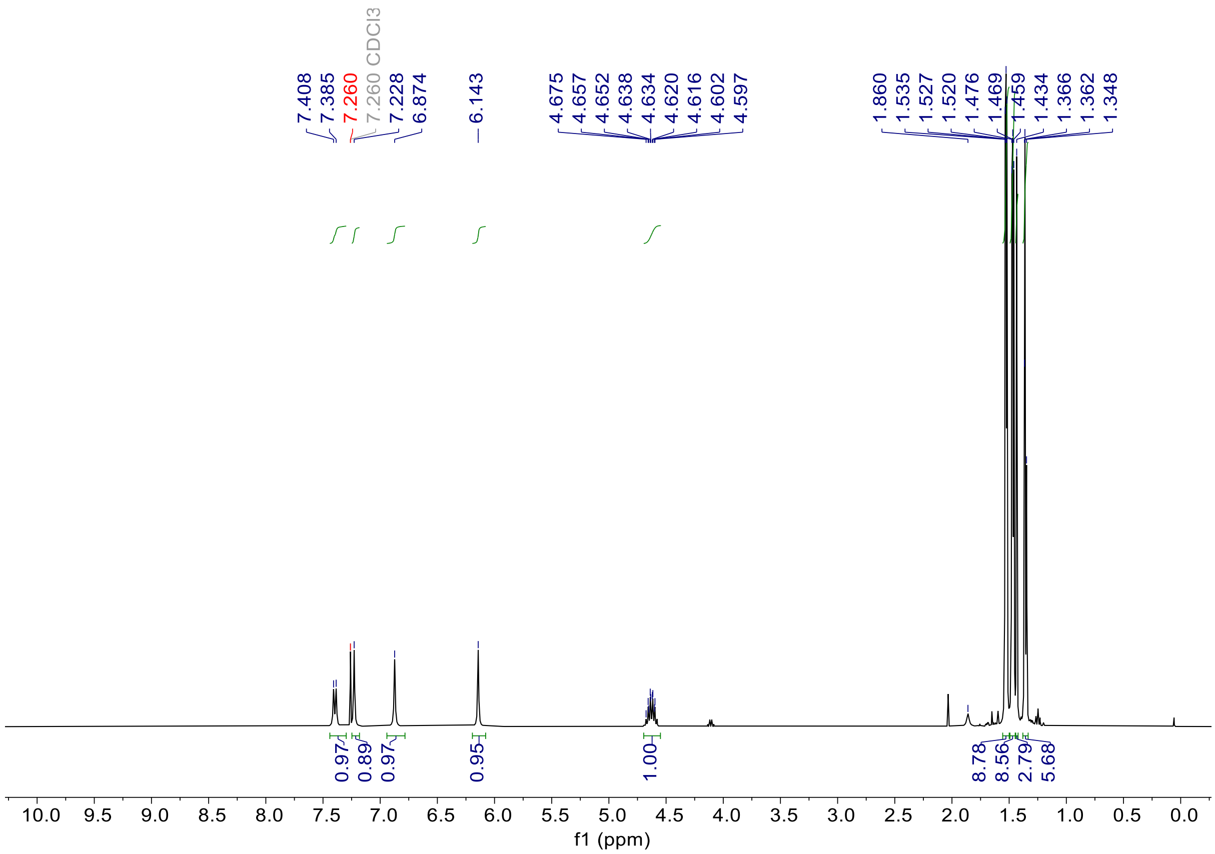

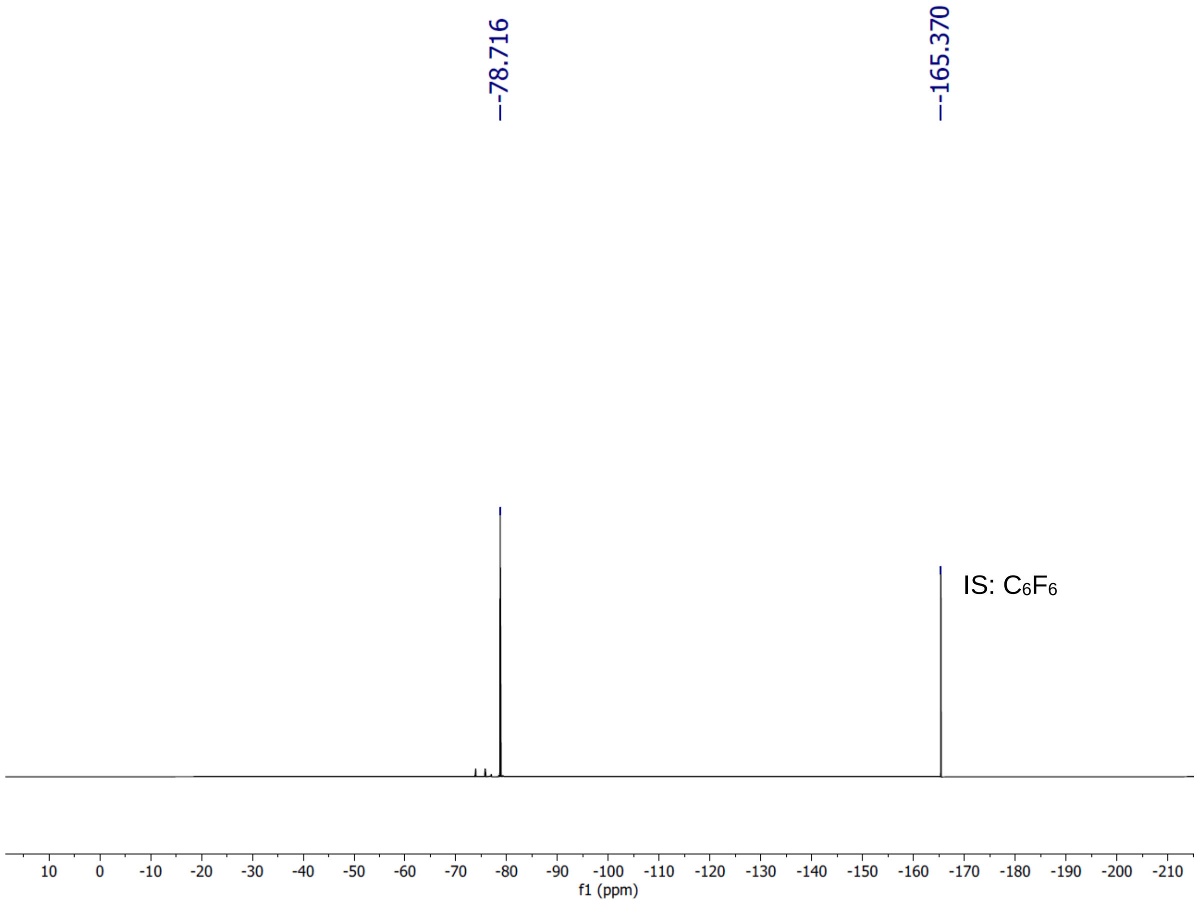

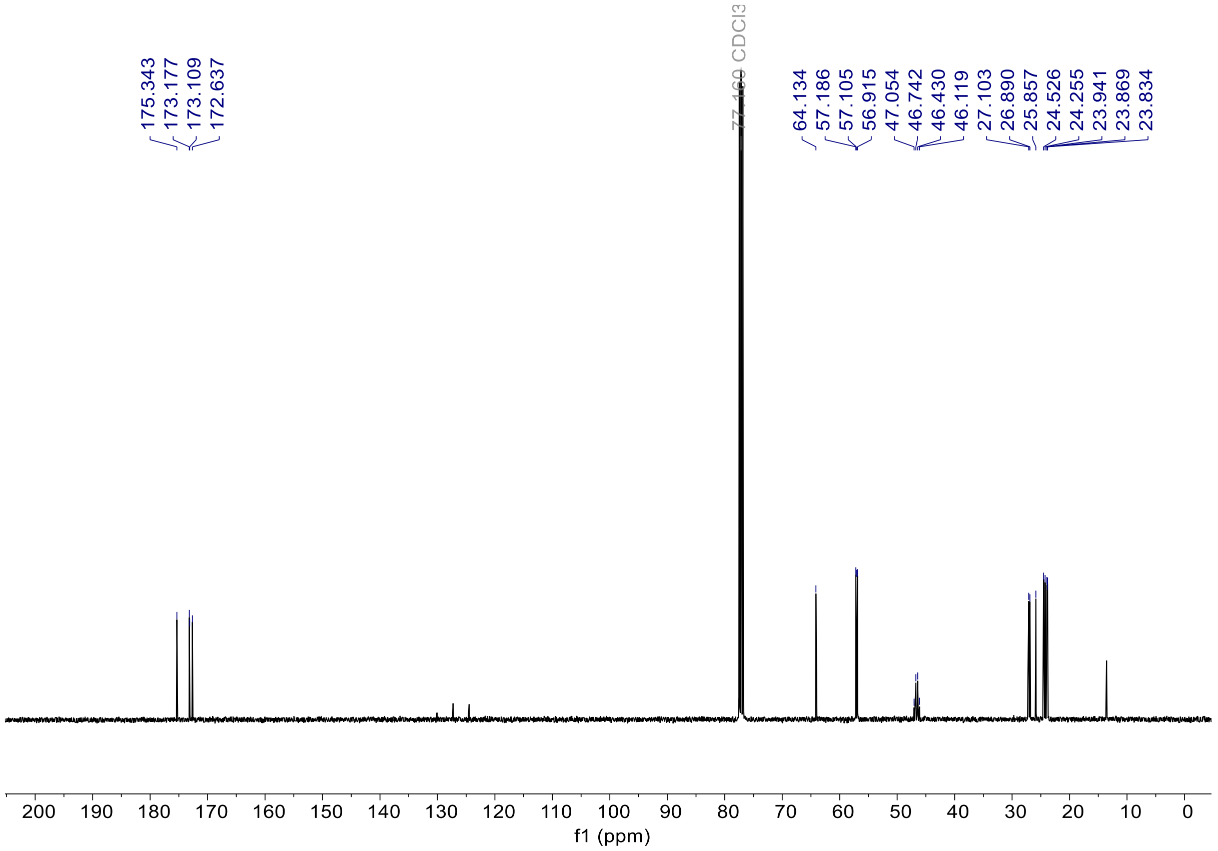
175.343
173.177
173.109
172.637
77.160 CDCl3
64.134
57.186
57.105
56.915
47.054
46.742
46.430
46.119
27.103
26.890
25.857
24.526
24.255
23.941
23.869
23.834
200 190 180 170 160 150 140 130 120 110 100 90 80 70 60 50 40 30 20 10 0
f1 (ppm)

13.9 N_3Aib_4(*S*-TFEA) (7)

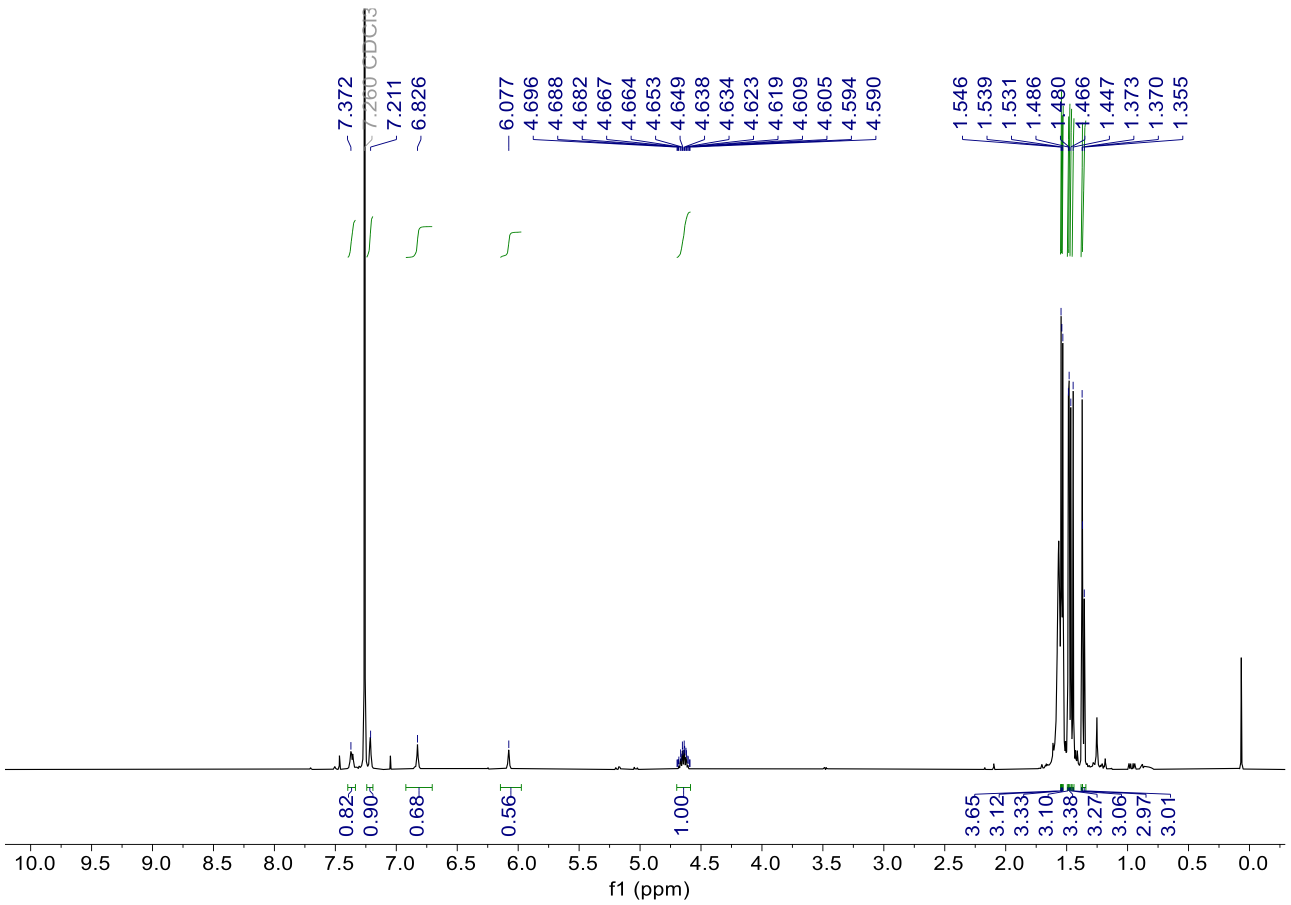

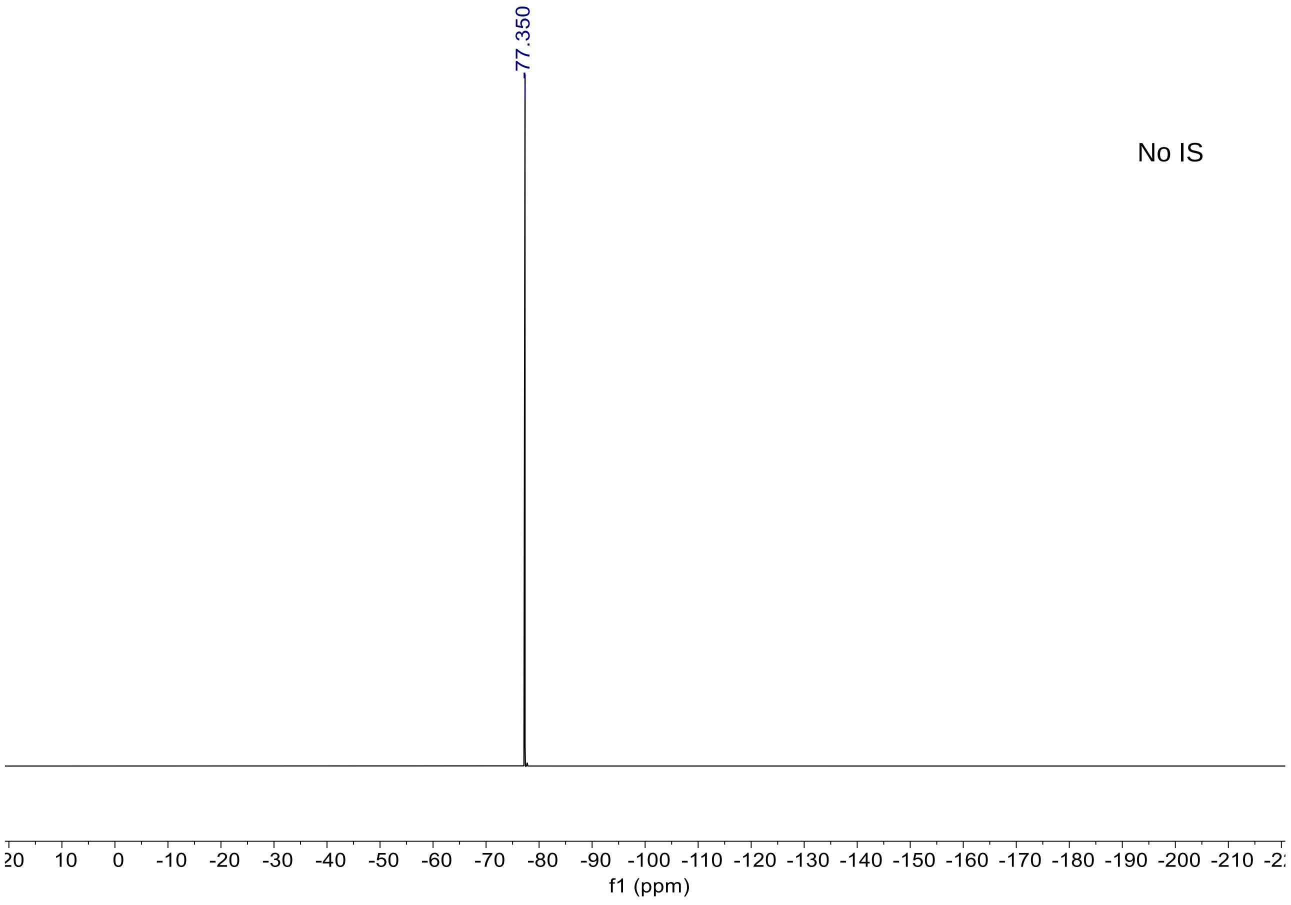

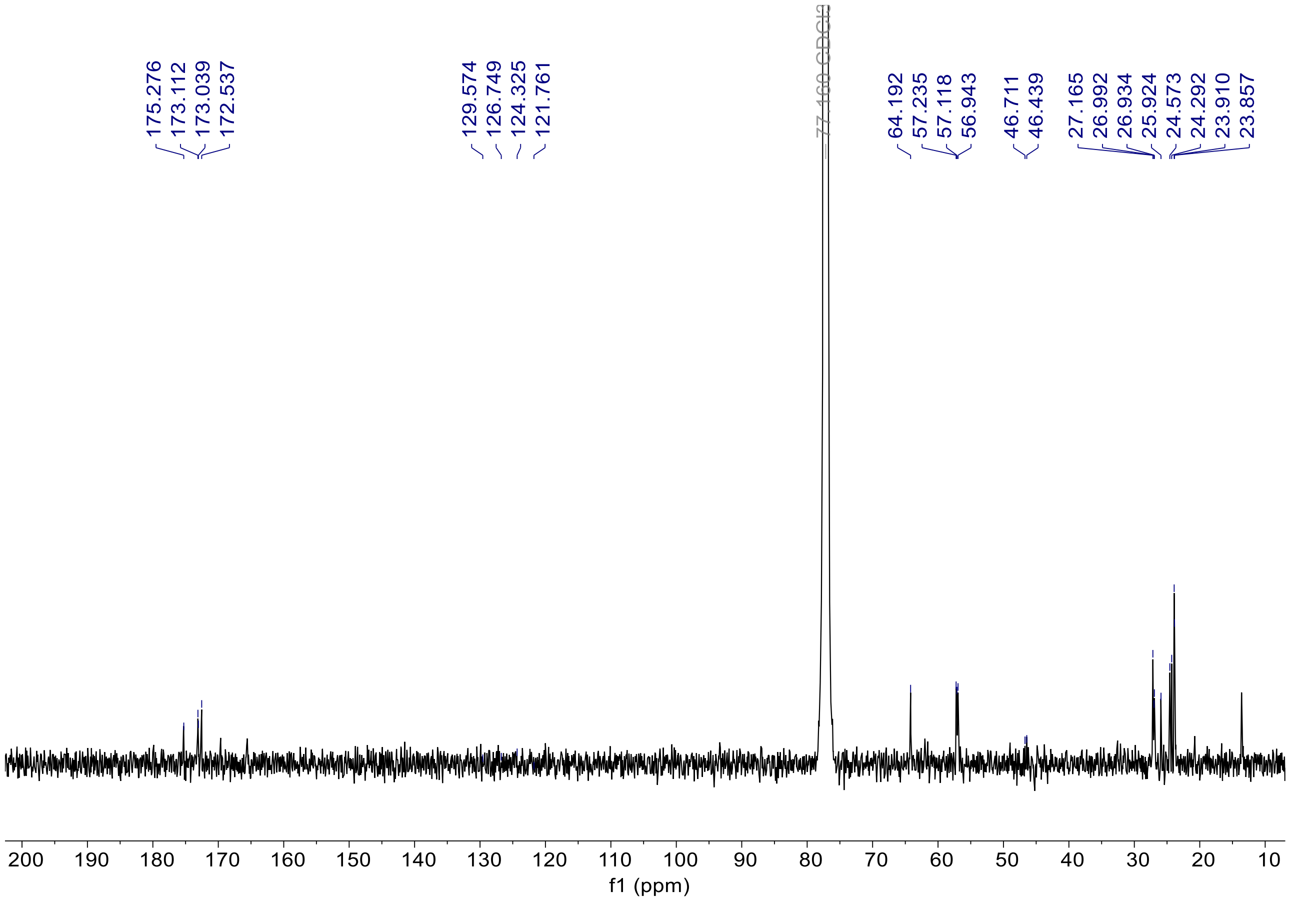
175.276
173.112
173.039
172.537
129.574
126.749
124.325
121.761
77.160 CDCl3
64.192
57.235
57.118
56.943
46.711
46.439
27.165
26.992
26.934
25.924
24.573
24.292
23.910
23.857
200 190 180 170 160 150 140 130 120 110 100 90 80 70 60 50 40 30 20 10
f1 (ppm)

14. References

(S1) Fulmer, G. R.; Miller, A. J. M.; Sherden, N. H.; Gottlieb, H. E.; Nudelman, A.; Stoltz, B. M.; Bercaw, J. E.; Goldberg, K. I. NMR Chemical Shifts of Trace Impurities: Common Laboratory Solvents, Organics, and Gases in Deuterated Solvents Relevant to the Organometallic Chemist. *Organometallics* **2010**, *29*, 2176–2179. https://doi.org/10.1021/om100106e.

(S2) Rosenau, C. P.; Jelier, B. J.; Gossert, A. D.; Togni, A. Exposing the Origins of Irreproducibility in Fluorine NMR Spectroscopy. *Angew. Chem. Int. Ed.* **2018**, *57*, 9528–9533. https://doi.org/10.1002/anie.201802620.

(S3) Hans Reich's Collection. NMR Spectroscopy. https://organicchemistrydata.org/hansreich/resources/nmr/?index=nmr_index%2F19F_shift#f-data00. Accessed on 21/08/2022.

(S4) Peters, A. D.; Borsley, S.; della Sala, F.; Cairns-Gibson, D. F.; Leonidou, M.; Clayden, J.; Whitehead, G. F. S.; Vitórica-Yrezábal, I. J.; Takano, E.; Burthem, J.; et al. Switchable Foldamer Ion Channels with Antibacterial Activity. *Chem. Sci.* **2020**, *11*, 7023–7030. https://doi.org/10.1039/d0sc02393k.

(S5) Byrne, L.; Solà, J.; Boddaert, T.; Marcelli, T.; Adams, R. W.; Morris, G. A.; Clayden, J. Foldamer-Mediated Remote Stereocontrol: > 1,60 Asymmetric Induction. *Angew. Chem. Int. Ed.* **2014**, *53*, 151–155. https://doi.org/10.1002/anie.201308264.

(S6) Clayden, J.; Castellanos, A.; Solà, J.; Morris, G. A. Quantifying End-to-End Conformational Communication of Chirality through an Achiral Peptide Chain. *Angew. Chem. Int. Ed.* **2009**, *48*, 5962–5965. https://doi.org/10.1002/anie.200901892.

(S7) (a) see http://community.dur.ac.uk/j.m.sanderson/science/downloads.html (b) Pike, S. J.; Diemer, V.; Raftery, J.; Webb, S. J.; Clayden, J. Designing Foldamer-Foldamer Interactions in Solution: The Roles of Helix Length and Terminus Functionality in Promoting the Self-Association of Aminoisobutyric Acid Oligomers. *Chem. Eur. J.* **2014**, *20*, 15981–15990. https://doi.org/10.1002/chem.201403626.

(S8) Lister, F. G. A.; Le Bailly, B. A. F.; Webb, S. J.; Clayden, J. Ligand-Modulated Conformational Switching in a Fully Synthetic Membrane-Bound Receptor. *Nat. Chem.* **2017**, *9*, 420–425. https://doi.org/10.1038/nchem.2736.

(S9) De Poli, M.; Byrne, L.; Brown, R. A.; Solà, J.; Castellanos, A.; Boddaert, T.; Wechsel, R.; Beadle, J. D.; Clayden, J. Engineering the Structure of an N-Terminal β-Turn To Maximize Screw-Sense Preference in Achiral Helical Peptide Chains. *J. Org. Chem.* **2014**, *79*, 4659–4675. https://doi.org/10.1021/jo500714b.

(S10) Reid, D. G.; Jackson, J.; Tew, D. G.; Gribble, A. D. A Fluorine-19 NMR Study of Interactions between the ATP-Citrate Lyase Inhibitor SB-200517 and Model Membranes. *Chem. Phys. Lipids* **1995**, *75*, 93–96. https://doi.org/10.1016/0009-3084(94)02396-M.

(S11) Joyce, R. E.; Williams, T. L.; Serpell, L. C.; Day, I. J. Monitoring Changes of Paramagnetically-Shifted 31P Signals in Phospholipid Vesicles. *Chem. Phys. Lett.* **2016**, *648*, 124–129. https://doi.org/10.1016/J.CPLETT.2016.02.007.

(S12) Stilbs, P.; Arvidson, G.; Lindblom, G. Vesicle Membrane-Water Partition Coefficients Determined from Fourier Transform Pulsed-Gradient Spin-Echo NMR Based Self-Diffusion Data. Application to Anesthetic Binding in Tetracaine-Phosphatidylcholine-Water Systems. *Chem. Phys. Lipids* **1984**, *35*, 309–314. https://doi.org/10.1016/0009-3084(84)90073-2.

(S13) Hinton, D. P.; Johnson, C. S. Diffusion Ordered 2D NMR Spectroscopy of Phospholipid Vesicles: Determination of Vesicle Size Distributions. *J. Phys. Chem.* **1993**, *97*, 9064–9072. https://doi.org/10.1021/j100137a038.

(S14) Kubasik, M.; Kotz, J.; Szabo, C.; Furlong, T.; Stace, J. Helix–helix interconversion rates of short ^{13}C-labeled helical peptides as measured by dynamic NMR spectroscopy. *Biopolymers* **2005**, *78*, 87–95. https://doi.org/10.1002/bip.20235.

(S15) Solà, J.; Morris, G. A.; Clayden, J. Measuring Screw-Sense Preference in a Helical Oligomer by Comparison of ^{13}C NMR Signal Separation at Slow and Fast Exchange. *J. Am. Chem. Soc*. **2011**, *133*, 3712–3715. https://doi.org/10.1021/ja1097034.

(S16) Bodero, L.; Guitot, K.; Lensen, N.; Lequin, O.; Brigaud, T.; Ongeri, S.; Chaume, G. Introducing the Chiral Constrained α-Trifluoromethylalanine in Aib Foldamers to Control, Quantify and Assign the Helical Screw-Sense. *Chem. Eur. J.* **2022**, *28*, e202103887. https://doi.org/10.1002/chem.202103887

(S17) Dolomanov, O. V.; Bourhis, L. J.; Gildea, R. J.; Howard, J. A. K.; Puschmann, H. OLEX2: A Complete Structure Solution, Refinement and Analysis Program. *J. Appl. Crystallogr.* **2009**, *42*, 339–341. https://doi.org/10.1107/S0021889808042726.

(S18) Sheldrick, G. M. SHELXT – Integrated Space-Group and Crystal-Structure Determination. *Acta Crystallogr.* **2015**, *A71*, 3–8. https://doi.org/10.1107/S2053273314026370.

(S19) Sheldrick, G. M. Crystal Structure Refinement with SHELXL. *Acta Crystallogr.* **2015**, *C71*, 3–8. https://doi.org/10.1107/S2053229614024218.
